# Arabinosylation of cell wall extensin is required for the directional response to salinity in roots

**DOI:** 10.1093/plcell/koae135

**Published:** 2024-05-01

**Authors:** Yutao Zou, Nora Gigli-Bisceglia, Eva van Zelm, Pinelopi Kokkinopoulou, Magdalena M Julkowska, Maarten Besten, Thu-Phuong Nguyen, Hongfei Li, Jasper Lamers, Thijs de Zeeuw, Joram A Dongus, Yuxiao Zeng, Yu Cheng, Iko T Koevoets, Bodil Jørgensen, Marcel Giesbers, Jelmer Vroom, Tijs Ketelaar, Bent Larsen Petersen, Timo Engelsdorf, Joris Sprakel, Yanxia Zhang, Christa Testerink

**Affiliations:** Laboratory of Plant Physiology, Wageningen University & Research, 6708 PB Wageningen, the Netherlands; Plant Cell Biology, Swammerdam Institute for Life Science, Universiteit van Amsterdam, 1090 GE Amsterdam, the Netherlands; Laboratory of Plant Physiology, Wageningen University & Research, 6708 PB Wageningen, the Netherlands; Plant Stress Resilience, Institute of Environmental Biology, Utrecht University, 3508 TB Utrecht, the Netherlands; Laboratory of Plant Physiology, Wageningen University & Research, 6708 PB Wageningen, the Netherlands; Laboratory of Plant Physiology, Wageningen University & Research, 6708 PB Wageningen, the Netherlands; Boyce Thompson Institute, Ithaca, NY 14853; Laboratory of Biochemistry, Wageningen University & Research, 6708 WE Wageningen, the Netherlands; Laboratory of Genetics, Wageningen University & Research, 6708 PB Wageningen, the Netherlands; Laboratory of Plant Physiology, Wageningen University & Research, 6708 PB Wageningen, the Netherlands; Laboratory of Plant Physiology, Wageningen University & Research, 6708 PB Wageningen, the Netherlands; Laboratory of Plant Physiology, Wageningen University & Research, 6708 PB Wageningen, the Netherlands; Laboratory of Plant Physiology, Wageningen University & Research, 6708 PB Wageningen, the Netherlands; Laboratory of Plant Physiology, Wageningen University & Research, 6708 PB Wageningen, the Netherlands; Laboratory of Plant Physiology, Wageningen University & Research, 6708 PB Wageningen, the Netherlands; Laboratory of Plant Physiology, Wageningen University & Research, 6708 PB Wageningen, the Netherlands; Plant Cell Biology, Swammerdam Institute for Life Science, Universiteit van Amsterdam, 1090 GE Amsterdam, the Netherlands; Department of Plant and Environmental Sciences, University of Copenhagen, Frederiksberg C 1871, Denmark; Wageningen Electron Microscopy Centre, Wageningen University & Research, 6708 PB Wageningen, the Netherlands; Wageningen Electron Microscopy Centre, Wageningen University & Research, 6708 PB Wageningen, the Netherlands; Laboratory of Cell Biology, Wageningen University & Research, 6708 PB Wageningen, the Netherlands; Department of Plant and Environmental Sciences, University of Copenhagen, Frederiksberg C 1871, Denmark; Molecular Plant Physiology, Philipps-Universität Marburg, 35043 Marburg, Germany; Laboratory of Biochemistry, Wageningen University & Research, 6708 WE Wageningen, the Netherlands; Laboratory of Plant Physiology, Wageningen University & Research, 6708 PB Wageningen, the Netherlands; College of Agriculture, South China Agricultural University, 510642 Guangzhou, China; Laboratory of Plant Physiology, Wageningen University & Research, 6708 PB Wageningen, the Netherlands

## Abstract

Soil salinity is a major contributor to crop yield losses. To improve our understanding of root responses to salinity, we developed and exploited a real-time salt-induced tilting assay. This assay follows root growth upon both gravitropic and salt challenges, revealing that root bending upon tilting is modulated by Na^+^ ions, but not by osmotic stress. Next, we measured this salt-specific response in 345 natural Arabidopsis (*Arabidopsis thaliana*) accessions and discovered a genetic locus, encoding the cell wall-modifying enzyme EXTENSIN ARABINOSE DEFICIENT TRANSFERASE (ExAD) that is associated with root bending in the presence of NaCl (hereafter salt). Extensins are a class of structural cell wall glycoproteins known as hydroxyproline (Hyp)-rich glycoproteins, which are posttranslationally modified by *O*-glycosylation, mostly involving Hyp-arabinosylation. We show that salt-induced ExAD-dependent Hyp-arabinosylation influences root bending responses and cell wall thickness. Roots of *exad1* mutant seedlings, which lack Hyp-arabinosylation of extensin, displayed increased thickness of root epidermal cell walls and greater cell wall porosity. They also showed altered gravitropic root bending in salt conditions and a reduced salt-avoidance response. Our results suggest that extensin modification via Hyp-arabinosylation is a unique salt-specific cellular process required for the directional response of roots exposed to salinity.

## Teaser

Salt induces EXTENSIN ARABINOSE DEFICIENT TRANSFERASE-dependent arabinosylation of hydroxyproline residues in extensin to modify cell wall structure and the gravitropism response of roots.

## Introduction

Because most crops are sensitive to salt stress, soil salinization is a major and increasing problem causing significant crop yield losses in agriculture, with more than 1 billion hectares of land already affected (Munns and Tester 2008; Zörb et al. 2019; Atta et al. 2023). Sodium chloride (NaCl) is the main factor causing soil salinity (Fao 2021), detrimentally affecting the plant on 2 levels: through osmotic stress, which inhibits water uptake by roots, and through excessive accumulation of ions in various plant tissues, leading to toxicity (Hasegawa et al. 2000; Julkowska and Testerink 2015). Excessive salt in soil obstructs a wide variety of cellular processes, such as impairing nutrient uptake, disrupting osmotic balance, and negatively impacting growth, development, and flowering, affecting crop yield (van Zelm et al. 2020). In the root, salt modifies root architecture and cell wall composition and inhibits gravitropism (reviewed in Zou et al. 2022). In response to salinity, cell wall modifications include increased lignin and suberin deposition and, in older tissues, the formation of secondary cell walls, all contributing to limiting Na^+^ entry (Voxeur et al. 2015; Barberon et al. 2016; Duan et al. 2020; Karlova et al. 2021). Moreover, the salt treatment also modulates several processes in cell wall biosynthesis, including cellulose synthesis, localization of cellulose microfibrils (Endler et al. 2015), galactan accumulation (Yan et al. 2021), and arabinose biosynthesis and metabolism (Zhao et al. 2019).

In this study, we performed a genome-wide association study (GWAS) of root growth parameters of 345 Arabidopsis (*Arabidopsis thaliana*) accessions by employing a novel dynamic salt-induced tilting assay (SITA) that we have developed. Similar to the halotropic response (Galvan-Ampudia et al. 2013; Deolu-Ajayi et al. 2019), in which roots typically move away from regions of high salinity (negative halotropism), the salt-induced modulation of root direction in SITA is a Na^+^-specific response that does not occur in response to osmotic stress or the presence of other monovalent cations, such as K^+^. We identified a region in the Arabidopsis genome that correlated with the ability of the root to change its direction of growth in response to salinity. Further investigation showed that this locus contains the gene encoding EXTENSIN ARABINOSE DEFICIENT TRANSFERASE (ExAD), an α-(1,3)-arabinosyltransferase that adds the fourth arabinose residue to a hydroxyproline (Hyp) residue of cell wall glycoproteins known as extensins. Extensins are Hyp-rich glycoproteins that are required to maintain the architecture of the primary cell wall (Lamport et al. 2011; Liu et al. 2016). Crosslinking of extensins helps to build up networks in the cell wall and contributes to the strength of the structural matrix (Cannon et al. 2008; Marzol et al. 2018). Extensins are posttranslationally modified first by hydroxylation and then subsequently by *O*-glycosylation, including serine-galactosylation and hydroxyproline (Hyp)-arabinosylation, which typically involves the addition of 4 arabinofuranose residues (Hyp-Araf_1-4_).

Several arabinosyltransferases are involved in forming the Hyp-Araf_1-4_ side chain at specific positions during Hyp-arabinosylation of extensins. HYDROXYPROLINE ARABINOSYLTRANSFERASES 1–3 (HPAT1-3) add the first arabinose (Hyp-Araf_1_) to Hyp residues (Ogawa-Ohnishi et al. 2013). Then, REDUCED RESIDUAL ARABINOSE 1–3 (RRA1–3), a *β*-(1,2)-arabinosyltransferase adds the second arabinose residue (Hyp-Araf_2_) (Egelund et al. 2007; Velasquez et al. 2011). Next, XYLOGLUCAN ENDOGLUCANASE 113 (XEG113), also a *β*-(1,2)-arabinosyltransferase, adds the third arabinose residue (Hyp-Araf_3_) (Gille et al. 2009), and finally, ExAD adds the fourth residue (Hyp-Araf_4_) (Møller et al. 2017). Recent studies suggest that extensin crosslinking requires the arabinosylation of extensin motifs (Cannon et al. 2008) and that the resulting crosslinked network promotes root defense against pathogen-derived elicitors and thus could limit colonization by pathogens (Castilleux et al. 2020). However, the role of arabinosylation in response to abiotic stress is yet unknown. Here, we reveal that ExAD is required both for the salt-specific modulation of the direction of root growth and for the enhanced thickness of root epidermal cell walls upon salt stress. We also demonstrate that the Hyp-Araf4-signal detected by a JIM11 antibody (Smallwood et al. 1994; Pattathil et al. 2010; Xie et al. 2011; Castilleux et al. 2020) is ExAD dependent and increases in salt-treated wilt-type seedlings, suggesting that cell wall extensin arabinosylation is a cellular response to salinity. Together, our study shows that Hyp-Araf_4_ of cell wall proteins is involved in root cell wall modifications under salt stress conditions and that ExAD is required for the modulation of root directional growth, revealing a crucial role for ExAD-mediated arabinosylation in root responses to salt stress.

## Results

### Root gravitropism is modified in a NaCl-specific manner

**Figure 1.**
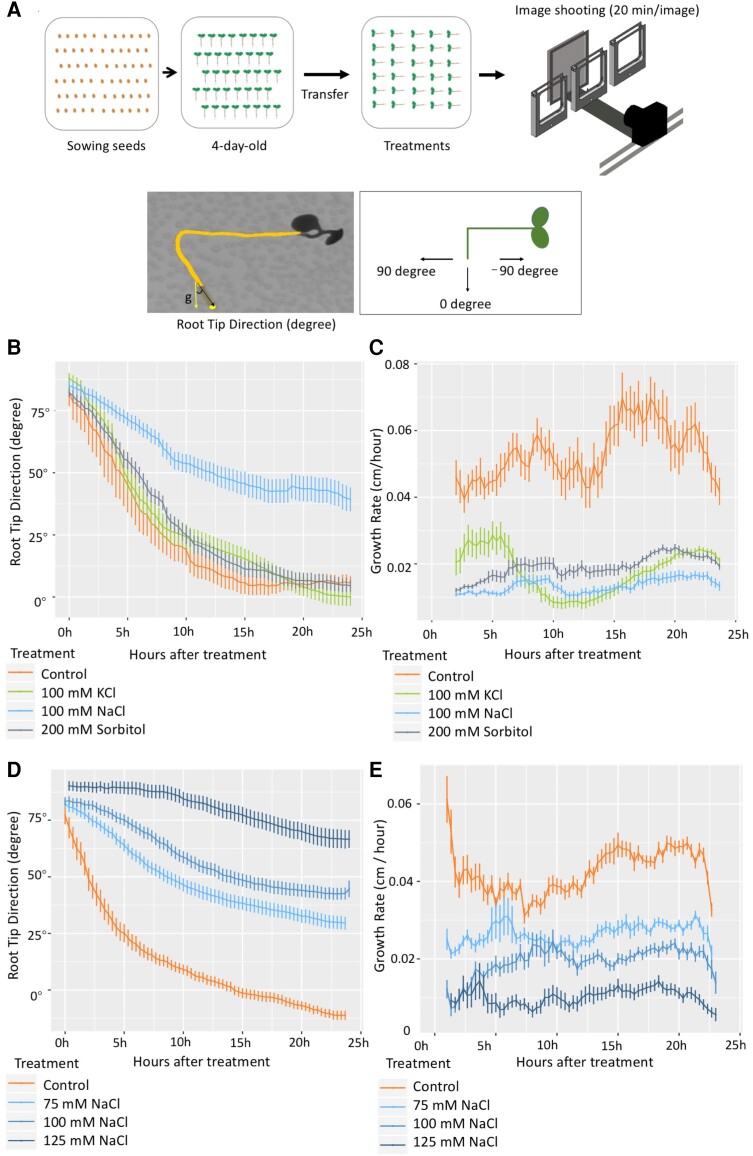
RTD in time-lapse SITA is specifically modulated by NaCl treatment in a dose-dependent manner. **A)** Experimental set-up of SITA. Four-day-old seedlings germinated on ½ MS medium were transferred to ½ MS agar plates containing different treatments, and at the same time, plates were rotated 90 degrees anticlockwise. The root dynamic growth response was traced in a time-lapse system with an imaging frequency of 1 image per 20 min per plate. **B)** RTD values expressed as degree angle (*y*-axis) were quantified every 20 min in time-lapse set-up in 4-d-old Col-0 seedlings under different treatments (control, 100 mm NaCl, 100 mm KCl, and 200 mm sorbitol). **C)** Growth rate of seedlings roots of Experiment B expressed in cm/h was analyzed over 24 h treatment. **D)** RTD values quantified in 4-d old treated Col-0 seedlings under different concentrations of NaCl (0, 75, 100, and 125 mm) over 24 h. **E)** The growth rates of roots treated as in **D)**. RTD angle values and root growth rates were obtained with SmartRoot. Values represent means ± SE from 30 seedlings, error bars represent SE of the mean. Data are representative of 3 independent experiments.

To understand the dynamics of root directional growth responses to salinity and investigate the sodium specificity of salt-induced inhibition of gravitropism, we devised a SITA setup in which different salts and osmotic treatments were applied, and dynamic root bending was recorded by a time-lapse imaging system. After 4-d-old Arabidopsis Col-0 seedlings were transferred to ½x MS agar plates containing different treatments (NaCl, KCl, sorbitol, or a control), the plates were rotated 90 degrees clockwise to apply a gravistimulus, immediately followed by tracking of root growth and direction in a time-lapse system. Images were taken every 20 min for at least 24 h (Fig. 1A, upper panel). During image analysis, we quantified the root tip direction (RTD) angle as the angle between the vector parallel to the direction of root tip growth and the vertical gravity vector, representing the amount of root tip bending (Fig. 1A, lower panel). The RTD angle was recorded every 20 min from the time after the seedlings were transferred to plates containing salt and calculated using only the direction of the root tip (the last 10% of the total root length). This decision was based on the observation that salt-treated root tips exhibit altered gravitropic responses (Dinneny et al. 2008; Sun et al. 2008). Angles of 0 to −90 degrees are typical of the roots of control-treated seedlings, whereas angles between 0 and 90 degrees are typical of salt-stressed roots. Consistent with previous studies (Dinneny et al. 2008; Sun et al. 2008), seedlings in our analysis using the SITA set-up displayed altered root tip directional growth, consistent with an altered root gravitropic response.

Changes in root bending, as measured by the RTD, were already detected at early time points (Fig. 1B). Interestingly, although a 100 mm NaCl treatment significantly affected the RTD (angles between 0 and 90 degrees), the RTDs of seedlings transplanted to media containing 200 mm sorbitol (equal osmotic pressure as medium with 100 mm of each of 2 ions) or 100 mm KCl were more similar to those in the control condition (Fig. 1B; Supplementary Movie S1), even though NaCl, KCl, and sorbitol treatments all resulted in a reduced root growth rate when compared with control conditions (Fig. 1C). This indicated that the difference in RTD angle was specific to NaCl and not likely caused by growth rate inhibition. NaNO_3_ treatment had a similar effect as NaCl, confirming that it was the presence of Na^+^ ions that triggered the altered root directionality in SITA (Supplementary Fig. S1A). To assess a possible effect of the root developmental stage, we repeated the experiments using 3-d-old Col-0 seedlings, and similar results were observed (Supplementary Fig. S1, B and C). We also tested the effects on 3-d-old seedlings of a 90-degree re-orientation of the plates either clockwise or counterclockwise direction. The response to a 90-degree re-orientation of the plates showed that after the initial phase, NaCl specifically modified root directional growth in both cases at a later stage, resulting in a consistent re-orientation to the right (Supplementary Fig. S1, D and E). Furthermore, we found a dose-dependent effect on these salt-dependent changes in root direction when testing a range of concentrations: 0, 75, 100, and 125 mm in SITA (Fig. 1, D and E), and we selected the concentration of 100 mm NaCl for subsequent analysis of a panel of natural Arabidopsis accessions.

### GWAS analysis of natural Arabidopsis accessions through SITA revealed candidate loci associated with alteration in salt-induced root directionality

Given the salt specificity of the SITA response, we used it as a phenotypic marker to identify new factors that alter the salt-dependent root bending response during gravistimulation. SITA root growth parameters were collected from 345 Arabidopsis HapMap accessions (Supplementary Table S1). From an initial survey performed on 20 of these Arabidopsis accessions, the overall root tip directional growth response observed in these accessions revealed a multi-phasic response pattern (Supplementary Fig. S2), characterized by 3 distinct phases (Phases I, II, and III) during the 30 h of treatment. Phase I (0 to 10 h) describes the acceleration stage in which the RTD of the tested accessions starts to respond to gravitropism. For most of the accessions, a strong effect of 100 mm NaCl on RTD was already observed during this phase, suggesting that the inhibition of gravitropism by salt occurs immediately. During Phase II (10 to 20 h), most of the accessions exhibited a similar rate of change in root growth direction under both control and NaCl conditions. In Phase III (after 20 h), root growth tended to stabilize in a final direction following the initial effects of both gravitropism and salt stress (Supplementary Fig. S2).

Based on the positive results obtained during this initial screen, we expanded our analysis to include the full HapMap diversity panel. The root phenotypic data obtained were subsequently analyzed for possible correlations with individual single nucleotide polymorphism (SNP) markers from the genome sequence of the Arabidopsis accessions (Alonso-Blanco et al. 2016) using a previously published GWAS R script based on EMMAX and the ASReml R package (v.3.5.0) (Korte et al. 2012). In parallel, the phenotypic data were analyzed with the online GWAPP web application (Seren et al. 2012). To map novel genetic loci associated with the salt-induced root-bending response, we selected two different traits to be used as input for GWAS. The SNPs that were identified for the early response to salt stress are shown in Supplementary Tables S2 and S3. The temporal traits K_RTD_^N/C^ (response in fitted rate of exponential decay) and root tip direction RTD^N-C^ (RTD with 100 mm NaCl minus RTD with 0 mm NaCl) were analyzed at 5 h (Supplementary Fig. S3, A, C, and D and Supplementary Table S3). Together, the natural variation data and SNPs that were identified for these temporal traits provide a useful resource for future investigation of early responses of roots to salt.

**Figure 2.**
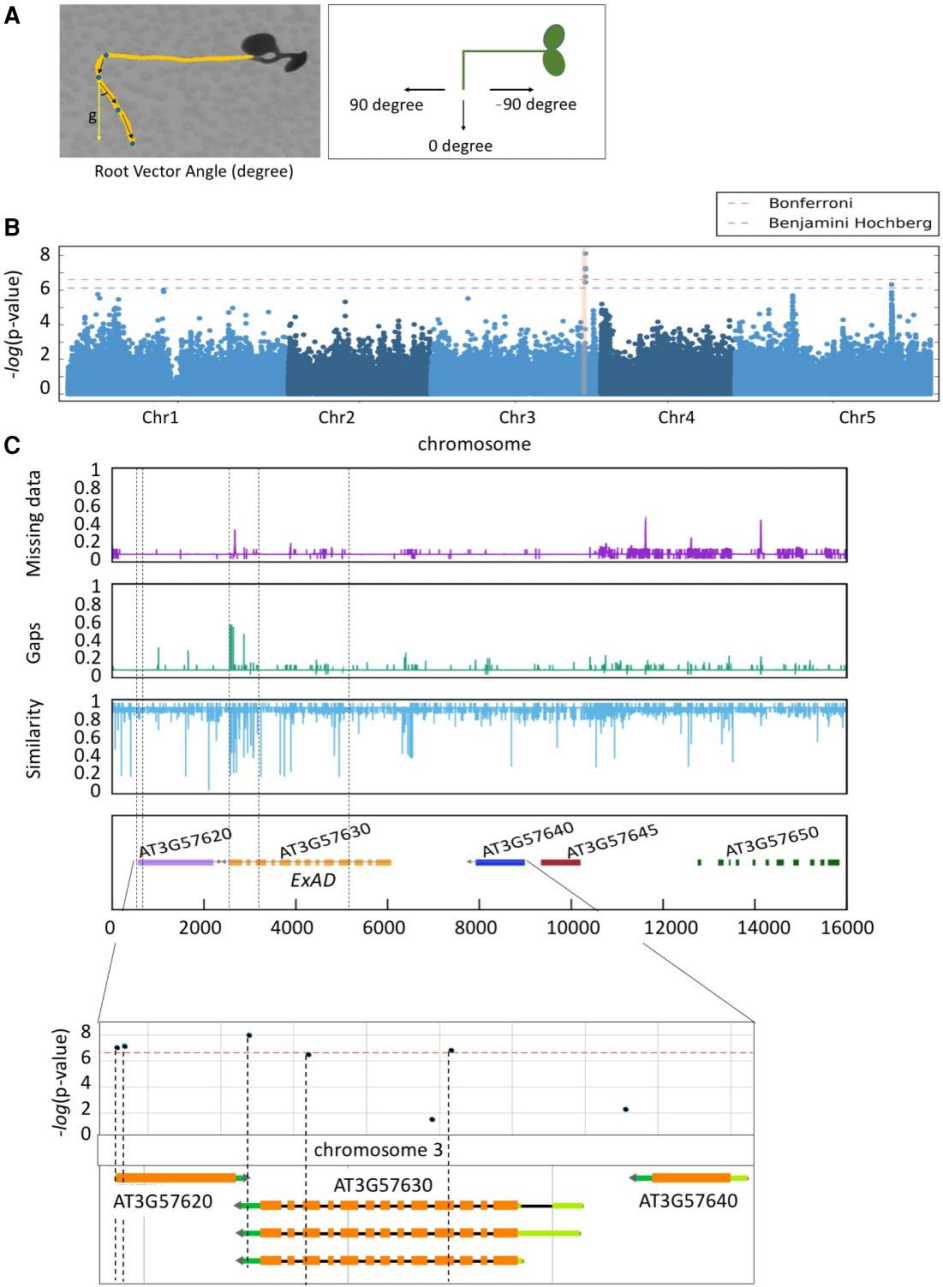
Natural variation in salt-modified root gravitropic response represented by RVA in SITA is associated with ExAD. **A)** RVA values, expressed as degree angle (*y*-axis) were quantified every 23 h on daily scans. Dots were marked at 0, 1, 2, and 3 d separately and arrows indicated the vector angle. **B)** Manhattan plots for the SNPs associated with response RVA (RVAN-C) at 23 h under salt condition subtracted by RVA under control condition. In the phenotyping experiment for the GWAS, 5 replicates (seedlings) on each plate were used to calculate the average per accession, and Col-0 was repeated in each round of time-lapse scanning as the standard control. QQ-plots are shown in Supplementary Fig. S4. **C)** Divergence plot showing the genetic variation surrounding the significant SNPs on Chr3 of all accessions in the 1001 genome database. The top purple graph represents the missing data, the middle green graph represents the gaps, and the bottom blue graph represents the similarity of SNPs compared with Col-0. Genes underlying this locus are listed in the lower panel and the location of SNPs is indicated with black dashed lines. The bottom graph zooms in the locus containing ExAD, and the location and corresponding −log10(*P*-value) score of the SNPs are shown accordingly using the GWAPP web application (Seren et al. 2012; Alonso-Blanco et al. 2016). The associations above the Bonferroni-corrected threshold are indicated by the red line.

Next, we focused on the overall root bending response, which we expressed as the root vector angle (or RVA), defined as the angle between the vertical gravity vector and the vector following the direction of the root tip from the moment of the tilting upward until 23 h (Fig. 2A). We measured RVA^N-C^ and observed that it was a highly consistent and robust trait displaying natural variation (Supplementary Fig. S3B). Hence, with the GWAPP web tool, we used the RVA^N-C^ at 23 h trait to identify a genetic locus on chromosome 3 (Chr3) that was associated with 5 significant SNPs at or above the Bonferroni-corrected threshold (Fig. 2B and Supplementary Table S2). Consistently, when using the ASReml model (Korte et al. 2012) and a panel of 4,285,827 SNPs, 5 SNPs above the limit of detection (LOD) 5.5 at the same positions were also found to be associated with the RVA^N-C^ trait (Supplementary Table S3 and Supplementary Figs. S3B and S4). The quantile–quantile plots indicate that the data from the 345 Arabidopsis accessions for both RVA^N-C^ and RAV^C^ at 23 h traits exhibit conformity and show a standard normal distribution.

To study the genetic variation in the selected locus, we focused on a region that was in linkage disequilibrium with the identified SNPs, spanning approximately 16,000 bp and containing all the identified SNPs in the candidate locus. Subsequently, we conducted a sequence alignment analysis using the Arabidopsis HapMap accessions sourced from the 1001 genome database (Weigel and Mott 2009) (Fig. 2C). Several regions were pinpointed as exhibiting missing data or having gaps, along with reduced overall similarity, and this correlated nicely with elevated levels of natural variation as deduced from sequence alignments across all accessions when compared with Col-0 (Fig. 2C, top three panels). Most of the low similarity peaks and significant SNPs with LOD scores above the Bonferroni-corrected threshold in Chr3 were found in the *ExAD* (*AT3G57630*) genomic region (Fig. 2C, lower panels). We analyzed natural variation in *ExAD* by using 3 significant SNPs (at positions 21339391, 21340210, and 21342174) among the 345 Arabidopsis HapMap accessions (Supplementary Fig. S5A and Supplementary Data Set 1) and 2 major haplotypes (Haplotype1 and Haplotype2) for the SNPs within *ExAD* were classified. The geographic distribution based on the original sampling position of the accessions belonging to Haplotype1 or Haplotype2 is shown in Supplementary Fig. S5B. Accessions with Haplotype1 displayed significantly higher RVA values after a 23-h salt treatment in SITA than Haplotype2 accessions (Supplementary Fig. S5C and Supplementary Data Set 1). We selected 3 accessions from each haplotype to test whether the expression of *ExAD* was altered due to the salt treatment. Overall, we found that the expression of *ExAD* in the accessions had already varied under control conditions (Supplementary Fig. S5D). However, the 48-h salt treatment did not cause changes in *ExAD* expression related to a specific haplotype. Two of the 3 SNPs for *ExAD* are located at exons (Exons 4 and 11) (Supplementary Fig. S5A), implying that the effect of the SNPs might not affect transcription in response to salt stress.

### Extensin Hyp-Araf_1-4_ arabinosyltransferase activity is required for SITA and halotropism

**Figure 3.**
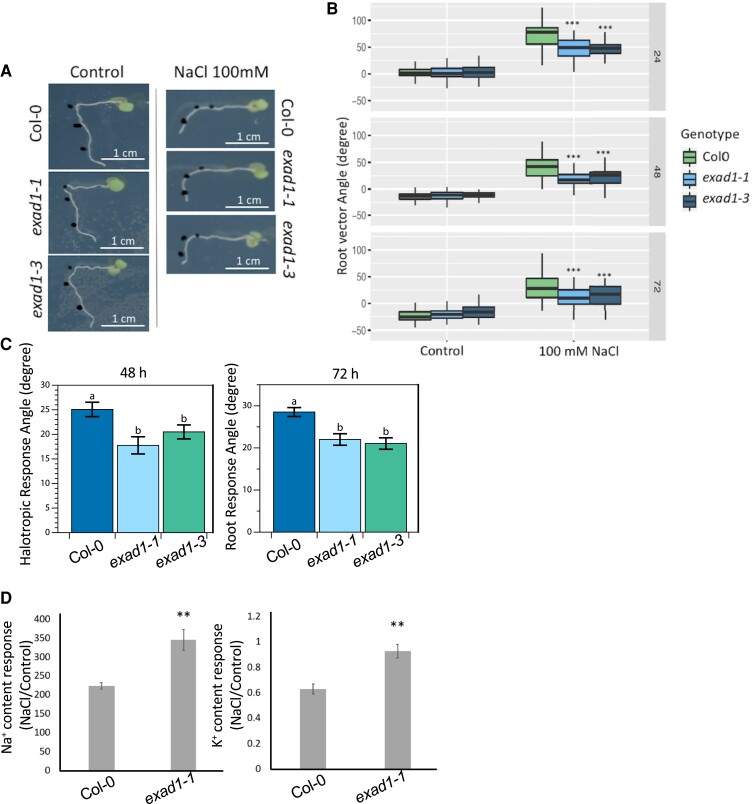
Extensin hyp-Araf1-4 arabinosyltransferase ExAD are involved in modifying RVA in salt stress. **A)** Representative image of Col-0, *exad1-1*, and *exad1-3* in SITA. The black dots indicate the location of root tips at 0, 24 and 48 h after transferring seedlings to the treatments (with or without 100 mm NaCl). **B)** Quantification of RVA of Col-0, *exad1-1*, and *exad1-3* mutants in SITA. Four-day-old seedlings were transferred to plates with or without 100 mm NaCl for 24, 48, and 72 h. Values represent means ± SEs from 10 biological replicates (plates) and 5 technical replicates (each plate containing 5 seedlings). Error bars represent SE of the mean. Statistical analysis was done using 2-way ANOVA with contrasts post-hoc. Asterisks indicate statistically significant differences compared with Col-0 (*P*-values ***P < 0.001; **P < 0.01; *P < 0.05). **C)** Quantification of relative root angles on root halotropic response, analyzed in 5-d-old seedlings treated with/without NaCl after 48 and 72 h on a gradient, show a reduced response of *exad1* mutant lines. Statistical analysis (*n* = 48) was performed with Shapiro–Wilk test (*P* < 0.05) followed by non-parametric Kruskal–Wallis test to determine group differences. Letters denote statistically significant differences according to Dunn’s test (*P* < 0.05). **D)** Na^+^ content response (NaCl treatment/control treatment) and K^+^ content response were measured in shoots of Col-0 and *exad1-1* mutant plants. Three-week-old plants were grown in hydroponic systems and were transferred to NaCl (150 mm) or control (0 mm) solutions for 4 d. The shoots were then harvested for ion measurement, and the data were normalized by fresh weight. Values represent means ± SEs of 4 biological replicates, each containing at least 2 shoots from different plants. Statistical analysis was performed using Student's *t*-test. Asterisks indicate statistically significant differences compared with Col-0 (**P < 0.01).

To further characterize the function of ExAD in regulating root directionality in the presence of salt, we used SITA to assess both the RVA and the root growth rates in two previously characterized Arabidopsis T-DNA knockout mutant lines [*exad1-1* (SAIL_843_G12) and *exad1-3* (SALK_204414C)] (Supplementary Fig. S6A) (Møller et al. 2017). Seedlings were transferred to agar plates with or without 100 mm NaCl and then gravistimulated. Root growth and bending were quantified after 24, 48, and 72 h. In both *exad1-1* and *exad1-3* mutant seedlings, we observed that the roots followed the direction of the gravity vector more closely in response to salt stress, showing less pronounced inhibition of root bending (Fig. 3A) during salt treatment compared to Col-0 seedlings (lower RVA values). No difference in root direction of the mutant seedlings was found under the control condition when compared with Col-0 seedlings (Fig. 3B). Consistently, when Col-0 and *exad1* mutant seedlings were exposed to a salt gradient, the *exad1* mutants exhibited a significantly reduced negative halotropic (salt avoidance) response (Fig. 3C and Supplementary Fig. S6B). Moreover, in a hydroponic system, adult *exad1-1* mutant shoots displayed enhanced Na^+^ and K^+^ accumulation in salt conditions compared to the wildtype (Fig. 3D and Supplementary Fig. S6, C and D).

Because similar growth rates were observed among the lines at the different time points analyzed (Supplementary Fig. S6, E and F), it is likely that the root bending differences detected in salt-treated seedlings were specifically linked to the functionality of ExAD rather than being influenced by alterations in growth patterns. To test this hypothesis, we examined mutants deficient in XEG113, an enzyme that catalyzes the addition of the third arabinose residue (Hyp-Araf_3_), just preceding the action of ExAD on extensins (Gille et al. 2009) (Supplementary Fig. S6G and Supplementary Table S4). Both *xeg113-1* and *xeg113-2* mutant roots showed a slightly less pronounced bending (lower RVA values) compared with the corresponding control roots, but the effect was only visible at 24 h following the initiation of salt treatment (Supplementary Fig. S6H), and it was accompanied by a slight change in root growth rate (Supplementary Fig. S6I). While *β*-arabinosyltransferases such as HPAT, RRA, and XEG113 exhibit a broad range of substrates (Matsubayashi 2014), ExAD stands out as the sole arabinosyltransferase responsible for mediating the addition of α-Araf to Hyp residues. To date, ExAD is considered to be specific to the regulation of extensins (Møller et al. 2017; Petersen et al. 2021). This specificity could potentially explain the observed difference between XEG113 and ExAD in controlling root bending responses under salt stress, the latter being more specific for the salt-dependent responses mediated by extensin modification.

### Salt increases ExAD-dependent arabinosylation in Arabidopsis seedlings

**Figure 4.**
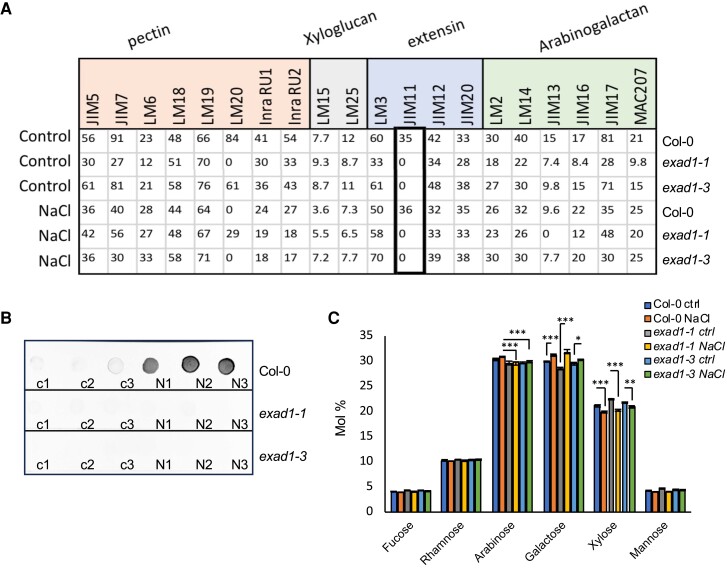
Anti-extensin monoclonal antibody JIM11 recognizes the ExAD-mediated arabinosylation. **A)** CoMPP analysis quantified mean spot intensities values (*y*-axis) showing the cell-wall glycans recognized by a selection of antibodies and CBMs within the pectin-enriched fraction (CDTA extraction). Five-day-old Arabidopsis seedlings of Col-0, *exad1-1*, and *exad1-3* mutants were transferred to the liquid treatment medium containing control (0 mm NaCl) or salt (100 mm NaCl) for 48 h and then harvested for cell wall AIR extractions. The list of the antibodies used can be found in **Supplementary Table S5**. Values represent means ± SEs from 3 biological replicates that were isolated. Full dataset of CoMPP heatmap for all extractions (CDTA and NaOH) are shown in Supplementary Fig. S8. **B)** Five-day-old seedlings of Col-0, *exad1-1*, and *exad1-3* were treated with or without 100 mm NaCl for 48 h before harvesting. Total protein was extracted and 25 *μ*g of total protein spotted on nitrocellulose membrane. JIM11 antibody was used to detect the specific Hyp-Ara_4_ signal. Dot-blot images are representative of 2 independent experiments performed each containing 3 biological replicates per treatment [Control (c1, c2, c3), or NaCl (N1, N2, N3)] per genotype. **C)** Five-day-old seedlings of Col-0, *exad1-1*, and *exad1-3* mutants were treated with 100 mm NaCl or without salt (Ctrl) for 48 h. Neutral cell wall matrix components were extracted from AIRs derived from 3 biological replicates per treatment/genotype and expressed as Molar Percentage (Mol%). Error bars represent SD of the mean values analyzed for each biological replicate (*n* = 3). Asterisks indicate statistically significant differences according to Student's *t*-test compared with Col-0 NaCl (for Arabinose) or compared with the corresponding controls (for galactose and xylose) (*P < 0.05; **P < 0.01 ***P < 0.001).

To further explore the effects of salt treatment on the extensin side chains (Hyp-Araf_1-4_) and detect the overall changes of cell wall polysaccharides, a qualitative survey of cell wall modifications was performed by using the Comprehensive Microarray Polymer Profiling (CoMPP) analysis as described in Moller et al. (2007). Col-0, *exad1-1* and *exad1-3* seedlings were treated with salt (100 mm NaCl) or left untreated as controls (0 mm NaCl) for 48 h before harvesting samples to assay for the alcohol insoluble residues (AIRs). 1,2-Cyclohexylenedinitrilotetraacetic acid (CDTA) and NaOH were used to perform sequential extractions to obtain CDTA (pectin-enriched) or NaOH (hemicellulose-enriched) fractions (Fangel et al. 2021). We quantified signal intensities derived from the recognition of cell wall epitopes using selected monoclonal antibodies (Supplementary Table S5). Overall, we found that in Col-0 and in both *exad1-1* and *exad1-3* seedlings, salt affected the signal intensities of antibodies involved in the recognition of pectin moieties, extensins, and arabinogalactans (Supplementary Fig. S7 and Supplementary Table S6). A previous study suggested that the LM1, JIM11, and JIM20 antibodies might be specific to the third Hyp-Araf_3_ or to higher order arabinosylation of extensin repeat side chains (Castilleux et al. 2020). However, to date, the exact targets of JIM11 are not known. Here, we report that JIM11 yielded no signal in the *exad1-1* and *exad1-3* mutant seedlings in either salt or control conditions, suggesting that JIM11 is likely involved in recognizing the ExAD-dependent Hyp-Araf_4_ (Fig. 4A, Supplementary Fig. S7, and Supplementary Table S6).

To make sure that JIM11 recognition signals were caused by an actual difference in extensin Hyp-Araf_4_ arabinosylation independent of the way the AIR samples were prepared, we extracted total proteins using a method adapted from Xu et al. (2011) and Leszczuk et al. (2020). A total of 25 *μ*g of total protein extracts from Col-0, *exad1-1*, and *exad1-3* seedlings treated for 48 h with salt (100 mm NaCl) or control were spotted onto nitrocellulose membranes and probed with an anti-JIM11 antibody (Fig. 4B). Interestingly, almost no signal was detected for seedlings containing the *exad1* mutant alleles. A similar signal was detected for the *xeg113-1* and *xeg113-2* mutant seedlings (Supplementary Fig. S8A) likely suggesting that both the ExAD- and the XEG-mediated arabinosylations, altering the overall Hyp-Araf_4_ content, are targets of anti-JIM11 recognition. Moreover, we observed a significant increase in the signal intensity in salt-treated Col-0 seedlings compared with control conditions (Fig. 4B and Supplementary Fig. S8A), suggesting that salt treatment induces an increase in the anti-JIM11 targets.

To quantify the arabinose levels in the cell wall matrix, we analyzed the monosaccharide composition of neutral sugars in AIRs extracted from seedlings of Col-0, *exad1-1*, and *exad1-3* mutants that were either treated for 48 h with NaCl or untreated controls (Fig. 4C). Our data show a clear arabinose-related phenotype in both the *exad1-1* and *exad1-3* mutants characterized by a mild reduction (likely being associated to the Hyp-Araf_4_ deficiency). We also detected a more pronounced reduction in arabinose content in the *xeg113-1* and *xeg113-2* mutant seedlings, likely dependent on an impairment in addition of both Hyp-Araf_3_ and Hyp-Araf_4_ (Supplementary Fig. S8B). Also, although we could confirm that salt triggered both an increase in galactose content and a reduction in xylose content (Fig. 4C and Supplementary Fig. S8B), a phenotype that seemed consistent in all the lines analyzed, we could not detect the significant salt-triggered reduction in cellulose content (Supplementary Fig. S8C) that has been proposed to be mechanistically linked to changes in galactose content (Yan et al. 2021). Cellulose levels appeared to be comparable in the mock-treated *exad1-1*, *exad1-3*, *xeg113-1*, *xeg113-2*, and Col-0 seedlings and hence do not seem to correlate with a change in the JIM11-dependent signal level and/or root bending phenotypes in controls (Supplementary Fig. S8C).

### ExAD is required for maintaining root cell wall thickness under salt treatment

**Figure 5.**
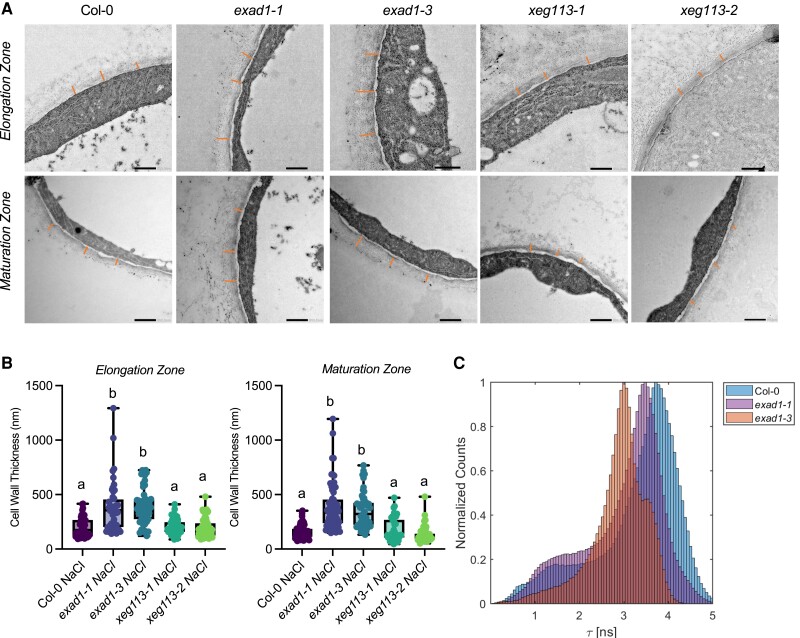
ExAD regulates root cell wall thickness under salt treatment. **A)** Cell wall structure of cells in epidermal layers in the root elongation zone (upper panel) and in the root maturation zone lower panel. Using the SITA system, 4-d-old Arabidopsis seedlings of Col-0, *exad1-1*, *exad1-3, xeg113-1*, and *xeg113-2* mutants were transferred 100 mm NaCl for 48 h before sampling for TEM analysis. Representative images are shown. **B)** Cell wall thickness of epidermal cells or maturation zone cells was measured in 3 biological replicates, each consisting of 16 to 20 independent cells. Cell walls were measured at 3 randomized positions on each of the analyzed cells per biological replicate. Letters indicate statistically significant differences between genotypes according to one-way ANOVA and Tukey's HSD test (*α* = 0.05). **C)** Effects of salt treatment changes cell wall mechano-chemical properties in an ExAD-dependent manner. Col-0, *exad1-1*, and *exad1-3* mutant seedlings were treated with 100 mm NaCl for 48 h. The intensity of the mechano-sensing probe described in Michels et al. (2022) displays altered cell wall porosity in the *exad1* mutant lines detected through FLIM analysis. The seedlings were stained with Cell Wall binding-Peptide-BODIPY (CWP-BDP in short) and fluorescence emission of elongation zone epidermal cells was captured on a Leica TCS SP8 inverted confocal microscope coupled to a Becker & Hickl TCSPC lifetime module (SPC830). Samples were excited with a 514-nm pulsed laser source (pulse duration < 1 ps) with a repetition rate of 40 MHz and plotted as an average of 3 independent experiments each containing 5 seedlings per genotype (*n* = 15).

To investigate whether the ExAD-dependent Hyp-arabinosylation induced by salt would alter cell wall structure, we imaged and quantified cell wall thicknesses in root epidermal cells by transmission electron microscopy (TEM). In our analyses, we also included xeg*113-1*, *xeg113-2* mutant seedlings in addition to *exad1-1*, *exad1-3*, and Col-0 control seedlings. Because *ExAD* is highly expressed in the root maturation zone (Møller et al. 2017), we examined cell wall thickness in both the root elongation and the maturation zones. When seedlings were treated with NaCl, a significant change in cell wall thickness was detected in the roots of both Col-0 seedlings and in the *exad1* loss-of-function mutant seedlings (Supplementary Fig. S9 and Fig. 5, A and B). Interestingly, the increase in cell wall thickness in response to salt was greater in the *exad1-1* and *exad1-3* mutant seedlings compared with wild-type seedlings and was seen in both the elongation and the maturation zones of the root (Fig. 5). In contrast, *xeg113* mutant seedlings did not show any statistically significant difference in cell wall thickness compared with Col-0 seedlings (Fig. 5B). Also no significant differences between *exad1-1* and Col-0 seedlings were detected in control conditions (Supplementary Fig. S9). Our results suggest a change in cell wall thickness in wild-type seedlings in response to salt stress that seems to be regulated by ExAD in a salt-dependent manner.

To delve more deeply into the structural changes of cell wall mechanics and properties in response to salt stress, we utilized a previously described fluorescent mechano-probe (Michels et al. 2022). The lifetime of the mechano-probe, which serves as an indicator of changes in cell wall density and strength, was measured using Fluorescence Lifetime Imaging Microscopy (FLIM). We collected emission spectra of the probe in the elongation zone of root epidermal cells of Col-0, *exad1-1*, and *exad1-3* seedlings that had been treated with 100 mm NaCl for 48 h (Fig. 5C, Supplementary Fig. S9E). Our analysis revealed that, in the absence of *ExAD*, the mechano-probe exhibited a significantly shorter lifetime under salt conditions. This observation likely indicates higher cell wall porosity associated with less dense cell walls and reduced cell wall strength. This finding aligns nicely with the TEM analysis, suggesting that in the absence of ExaD, the extensin-dependent crosslinks, which appear to be altered in the presence of salt, affect the properties of the cell walls, resulting in decreased rigidity and swollen cell walls.

## Discussion

Salt stress negatively affects the growth and development of roots in most plant species (Julkowska et al. 2014; van Zelm et al. 2020), and this has a significant negative effect on crop yields. Roots can change their growth direction dynamically to rapidly avoid a saline environment, a response that can be used as a phenotypic marker to study salt-signaling pathways (Deolu-Ajayi et al. 2019). In response to salt gradients, roots bend away from areas of high salt, a process called halotropism. The mechanisms of how root halotropic responses are accomplished by redistribution of auxin have been studied in recent years (Galvan-Ampudia et al. 2013; Korver et al. 2020). On the other hand, in uniform salt conditions, little is known about the regulatory pathways and genetic components that are required for salt-induced directional growth (Sun et al. 2008). In this study, we have developed and performed a SITA to investigate root directional growth upon gravitropic challenge in response to salt. We found responses of roots in the SITA could be modulated by salt, specifically by Na^+^ ions, but not by an equivalent level of osmotic stress (Fig. 1, B and D and Supplementary Fig. S1A). This finding is akin to the halotropic response, which is also specific to Na^+^ ions as demonstrated by previous studies (Galvan-Ampudia et al. 2013; Deolu-Ajayi et al. 2019).

Using GWAS, we identified genetic components that appear to control root directional growth by screening a collection of 345 natural Arabidopsis accessions. Both phenotypic data and associations presented here are useful resources for follow-up approaches to understand Na^+^-specific responses in roots. All raw phenotyping data are available via the public repository (https://github.com/YutaoYutao/S-root). Here, we focused on the characterization of one of these newly identified genes, which encodes ExAD, an arabinosyltransferase that adds the fourth arabinofuranose (Hyp-Araf_4_) on the continuous stretches of adjacent Hyp residues found in cell wall-localized glycoproteins, such as the plant extensins. Extensins are repetitive Hyp-rich *O*-linked glycoproteins that have been suggested to modulate plant primary cell wall architecture (Hijazi et al. 2014). In our study, the *exad1-1* and *exad1-3* loss-of-function mutant seedlings showed reduced salt-induced modulation of root gravitropic bending and halotropic responses (Fig. 3), and a significantly thicker cell wall under salt conditions (Fig. 5, B and C), phenotypes that may be linked to defects in extensin crosslinking in the *exad1* mutants.

Crosslinked extensins play crucial roles in supporting and maintaining cell wall architecture and cell wall formation, regulating plant growth and development, and contributing to disease and wounding resistance (Cannon et al. 2008; Lamport et al. 2011; Lamport and Várnai 2013). The various types of crosslinks that are composed of different cell wall polymers can be classified based on their homopolymeric and heteropolymeric crosslinks (Mishler-Elmore et al. 2021). Heteropolymeric crosslinks are found between two different cell wall polymers, including pectin and cellulose (Pérez García et al. 2011), pectin and extensin, pectin and hemicellulose, Arabinogalactan-protein and extensin, and extensin and lignin, as summarized by Mishler-Elmore et al. (2021). Extensins can also form homopolymeric crosslinks, which require correct Hyp-arabinosylation. The extensin peroxidases PEROXIDASE9 (PRX9) and PEROXIDASE40 (PRX40) were proposed to contribute to extensin crosslinking during pollen development in Arabidopsis (Jacobowitz et al. 2019). Interestingly, arabinosylation, in particular, the ExAD-dependent Hyp-α-arabinosylation to form Hyp-Araf_4_, may also be crucial for extensin crosslinking in vitro (Chen et al. 2015). A biotic stress study suggested that extensin arabinosylation is involved in cell wall formation and maintenance and can help to protect against infection by the oomycete *Phytophthora parasitica* (Castilleux et al. 2020). Most recently, it has been hypothesized that defects in extensin arabinosylation in the Hyp-Araf mutants cause deficiency in extensin crosslinking that can lead to cell wall architecture changes and eventually decrease plant defenses (Castilleux et al. 2020; Tan and Mort 2020).

Here, using TEM analysis, we showed the involvement of ExAD-dependent Hyp-α-arabinosylation in regulating root cell wall structure under salt treatment (Fig. 5B). This seems to be linked both to alterations of root bending in response to salinity stress and the ability of roots to avoid high salinity by changing their direction of growth (halotropism) (Fig. 3). Shoots of *exad* mutant plants that were exposed to salinity had higher levels of Na^+^ and K^+^, suggesting that the uptake of anions, in general, was increased in the mutants. Moreover, by using a cell wall mechano-probe (Michels et al. 2022), we found that cell wall density and porosity depended on the function of ExAD and was thus likely linked to the ExAD-mediated Hyp-α-arabinosylation that was triggered by exposure to salt. Salt stress induces several cell wall-localized responses, such as changes in polysaccharide deposition, changes in pectin properties and microfibril orientation, all of which can compromise cell wall function (Byrt et al. 2018). Reduction of cellulose content has been reported before (Endler et al. 2015; Yan et al. 2021), but under our experimental conditions, we did not detect changes in cellulose content. It is possible that at the time point analyzed here (48 h) following induction of salt stress, a reduction in cellulose content was not yet evident, although the extensin-mediated phenotype could already be quantified.

Recently, the cell wall has been hypothesized to be crucial for salt tolerance and salt-signaling pathways (Feng et al. 2018; Gigli-Bisceglia et al. 2022). The *Catharanthus roseus* receptor-like kinase 1-like (CrRLK1L) protein kinase subfamily member FERONIA (FER) and the HERKULES 1/THESEUS 1 combination have been suggested to be responsible for triggering several salt response signaling pathways that depend on the detection of salt-induced pectin modifications (Feng et al. 2018; Gigli-Bisceglia et al. 2022). As a chimeric class of extensins, leucine-rich repeat extensins (LRXs) members LRX1 and LRX2 were suggested to be involved in root hair formation and cell morphogenesis via mediation of cell wall development (Baumberger et al. 2001, 2003). LRX3, 4, and 5 were shown to be required for plant salt tolerance via binding of Rapid Alkalinization Factor (RALF) peptides and their direct interaction with FER to transduce the cell wall signals in response to salt stress (Zhao et al. 2018). The *exad1* loss of function mutants were reported to have shorter root hairs (Møller et al. 2017), similar to other extensin Hyp-β-arabinosyltransferase mutants, such as the *rra* (responsible for Hyp-Araf_2_) and *xeg113* (responsible for Hyp-Araf_3_ formation) mutants (Egelund et al. 2007; Gille et al. 2009; Velasquez et al. 2011). In addition, *hpat* mutants (responsible for Hyp-Araf_1_) were reported to have defects both in cell wall thickness and root hair elongation (Ogawa-Ohnishi et al. 2013; Velasquez et al. 2015). Whether the salt-triggered ExAD-dependent phenotypes are linked to reduced root surface area due to shorter hairs, as previously suggested to alter salt sensitivity (Robin et al. 2016), is yet unclear.

Here we show that changes in root bending response in salt stress seem to be likely dependent on changes in cell wall width and resulting mechanical changes. Based on this work, we now suggest that extensin arabinosylation constitutes another type of cell wall modification, one that is not necessary for root growth but is crucial for the directional response to salt stress. Extensins, as structural cell wall glycoproteins, appear to play a vital role in maintaining the normal physical structure of the cell wall under stress conditions. We hypothesize that salt-induced cell wall modifications and the associated changes in turgor pressure activated the ExAD enzyme, which is involved in increasing extensin arabinosylation to enhance cell wall strength under challenging conditions. In contrast, in physiological conditions when the cell wall is unchallenged, enhanced crosslinking is not required, making ExAD dispensable. However, in situations where cell walls are under stress, the function of ExAD becomes essential to counteract changes in the structural integrity of the cell wall. Hence, in its absence, the cell wall might become weaker and more porous, more hydrated, and thicker (Fig. 5). We propose that salt-induced extensin arabinosylation is a critical mechanism for reinforcing the cell wall and mitigating the damage caused by Na^+^ ions, enabling roots to respond effectively to salinity stress.

In summary, we found that salt stress affected cell wall composition and structure in an ExAD-dependent manner, demonstrating that ExAD α-arabinosylation of extensin to yield Hyp-Araf_4_ was required to increase extensin crosslinking and modify cell wall structure during salt stress. Our SITA time-lapse evaluation of root growth of a set of natural accessions in salt revealed that cell wall modification processes/changes are a crucial aspect of root responses to salinity, similar to their importance for biotic stress perception (Gigli-Bisceglia and Testerink 2021). How this process integrates with other reported salt-induced cell wall modifications and how they together function in root responses and plant performance in saline conditions remains to be established.

## Materials and methods

### Plant materials and growth conditions

All seeds used in this study (listed in Supplementary Tables S1 and S4) were propagated under greenhouse conditions. *Arabidopsis thaliana* (Arabidopsis) seedlings for all of the phenotypic assays in this study were grown under long-day conditions [16 h under white 125 *μ*mol m^−2^ s^−1^ LED light (Lumeco 4000 K)/8 h dark)], 21°C, 70% humidity. For SITA, seeds were surface sterilized in a desiccator and were placed next to a beaker filled with 40 mL bleach and 1.2 mL 37% HCl for 3 h, followed by at least 3 h in a laminar flow hood to remove the extra surface bleach. For other experiments, seeds were surface sterilized in 30% (v/v) bleach with Triton X-100 (10 *µ*L per 50 mL) for 10 min, followed by washing with sterilized Milli-Q water at least 6 times. Disinfected seeds were stratified at 4°C for 3 d in darkness before being sown on agar plates. Seeds of mutant plants *exad1-1* (SAIL_843_G12), *exad1-3* (SALK_204414C) were previously characterized (Møller et al. 2017). The *xeg113-1* (SALK_151754) and *xeg113-2* (SALK_066991) mutants were ordered from NASC. Detailed information for seeds is listed in Supplementary Table S4. For SITA, ½x MS (including B5 vitamins, Duchefa Biochemie B.V.) medium containing 0.1% 2(*N*-morpholino) ethanesulphonic acid (MES) buffer (Duchefa) and 0.5% sucrose (Duchefa), pH 5.8 adjusted with KOH, 1% Daishin agar (Duchefa) was used. For salt treatment, NaCl was added after adjusting the pH to 5.8. In the salt specificity experiment, treatments of 0 mm NaCl (Control), 100 mm NaCl, 100 mm NaNO_3_ 200 mm sorbitol, and 100 mm KCl were added to the medium. As a control to maintain the same osmotic pressure, the sorbitol concentration was twice that of the salt solutions, which have 100 mm of each of the two ions. In the salt dose–response experiment, treatments were 0 (Control), 75, 100, and 125 mm NaCl as indicated in Figure legends. In all other experiments, treatments were 0 and 100 mm NaCl. For gene expression analysis of ExAD1 in T-DNA mutants (*exad1-1* and *exad1-3*), seeds were germinated and grown for 8 d before harvesting on ½x MS (including B5 vitamins, Duchefa Biochemie B.V.) plates containing 0.5% sucrose, 0.1% MES (Duchefa), 1% Daishin agar (Duchefa), and pH 5.8 adjusted with KOH. For gene expression analysis of ExAD in tested accessions from two haplotypes, seeds were germinated and grown in a flask of liquid ½x MS (including B5 vitamins, Duchefa Biochemie B.V.) medium, 0.1% MES (pH 5.8), 0.5% sucrose, with shaking at 130 rpm under long-day conditions. Following germination, 4-d-old seedlings were transferred to a medium containing 0 or 100 mm NaCl for 48 h before harvesting. For dot-blot assays, seeds were germinated and grown in a flask of liquid ½x MS (including B5 vitamins, Duchefa Biochemie B.V.) medium containing 0.5% sucrose and 0.1% MES (pH 5.8), with shaking at 130 rpm under long-day conditions. Four-day-old seedlings were transferred to 0 or 100 mm NaCl for 48 h before harvesting. For CoMPP analysis, seeds were germinated and grown in a flask of liquid ½x MS (including B5 vitamins, Duchefa Biochemie B.V.) medium containing 0.5% sucrose and 0.1% MES (pH 5.8), with shaking at 130 rpm under long-day conditions. Four-day-old seedlings were transferred to a medium containing 0 or 100 mm NaCl for 48 h before harvesting. For ion content analysis in the hydroponic system (https://www.araponics.com/), 3-wk-old Arabidopsis Col-0 and *exad1-1* mutant plants were grown hydroponically and treated with 0 or 150 mm NaCl for 4 d. Liquid medium was used [½x MS (including B5 vitamins, Duchefa Biochemie B.V.), 0.1% MES, pH 5.8]. Shoots were harvested and approximately 150 mg of fresh plant material was harvested and rinsed 3 times with Milli-Q water. Next, the shoots were submerged in a solution of 10 mm CaCl_2_ for 5 min and washed with Milli-Q water; submerged again in 10 mm EDTA for 5 min and washed with Milli-Q water (a total of 3 times). All samples were dried at 100°C for 48 h prior to ion measurement. Ion measurements were performed by ICP-MS as described in Danku et al. (2013) at the Ionomics Facility, University of Nottingham, UK.

### Salt-induced tilting assay

Arabidopsis seeds were surface sterilized in a desiccator and were placed next to a beaker filled with 40 mL bleach and 1.2 mL 37% HCl for 3 h, followed by at least 3 h in a laminar flow hood to remove the extra surface bleach. The seeds were then stratified in a 0.1% Daishin agar solution at 4°C in the dark for 3 d before sowing. After stratification, seeds were germinated on ½x MS plates containing 1% agar that were placed in 70-degree angle racks for 4 d (80 seeds per plate). Four-day-old Arabidopsis Col-0 seedlings were transferred to ½x MS agar plates containing different amounts of solutes (NaCl, KCl, and sorbitol). After transferring seedlings to these plates, all plates were rotated 90 degrees clockwise simultaneously to apply a gravistimulus and placed in vertical (90 degree) racks. In the time-lapse SITA system, all plates were imaged every 20 min by infrared photography for at least 24 h. Alternatively, root tips were marked at 24, 48, and 72 h as indicated in figure legends.

### Halotropic response analysis

Halotropism assays on salt gradients were performed as described previously (Galvan-Ampudia et al. 2013; Deolu-Ajayi et al. 2019). Arabidopsis seeds (Col-0, *exad1-1*, and *exad1-3*) were surface sterilized as described above and stratified in a 0.1% Daishin agar solution at 4 °C in the dark for 3 d before sowing. After stratification, seeds were germinated on plates containing ½x MS medium, 1% agar, and 0.5% sucrose in 90-degree angle racks for 5 d. After 5 d, a 45° cut-out of the medium was made at 0.2 cm below the root tips and replaced with medium supplemented with/without 200 mm NaCl. Plates were scanned with an Epson Perfection V800 Scanner at 48 and 72 h after the treatment. The images were analyzed with Smartroot plugin (Lobet et al. 2011).

### Root phenotypic quantification and data processing

In the SITA system, images of plates were captured every 20 min in jpg format, at 690 dpi. Alternatively, images of plates were scanned with an Epson Perfection v800 Photo scanner at 400 dpi in jpg format every day. All images were converted to black/white in tiff format, and each root was traced and quantified in ImageJ, SmartRoot plugin. Two root traits were used for quantifications: the RVA was measured from the beginning of the root-to-root tip as shown by the white arrow; the RTD was determined from the last 10% of the root tracing length (Fig. 1A). The temporal trait K^N/C^ (response in fitted rate of exponential decay) was obtained by fitting a model of exponential decay $\left(\;y=y0+\frac{L}{1+e^{-kt}}\;\right)$ to the changes in RTD (*y*) over time (*t*) for every accession in both salt and control conditions. In this model, *y*₀ represents the lower asymptote, *L* is the initial decrease in angle from the starting value to the lower asymptote, and *k* is the rate of exponential decay. The parameter *k* was used in the GWAS as a response variable differing between salt and control conditions. The time-lapse system can hold a maximum of 8 plates vertically in a row and has the camera (Canon 1200D) on a rail in front of plates that can move and take photographs. For each plate, the images were first traced manually with SmartRoot for the last time-point image, and then a SmartRoot plugin—automatic tracing—was used to trace images back at earlier time points. All the nodes during tracing were copied from the previous images to the next image, with simultaneous detection of the pixel intensity to remove the extra nodes from the root tip. After automatic tracing, nodes in each image were inspected and adjusted manually if needed. SmartRoot was used in both time-lapse and scanning system tracing and output data into a .csv file, available at https://smartroot.github.io/ (Lobet et al. 2011). R was used for data and graph processing (available at https://github.com/YutaoYutao/S-root).

### Genome-wide association study

Root SITA response traits of the HapMap accessions were collected using a time-lapse system. In the phenotyping experiment for GWAS, 5 replicates (seedlings) for each accession on each plate were used to calculate the average used as input. Col-0 seedlings were included in each round of time-lapse scanning as the control. The phenotypic data were used in concert with SNP markers from the 1001 genome project, and a GWAS was performed using two association mapping tools: GWAPP (Seren et al. 2012; Alonso-Blanco et al. 2016) and R using the EMMAX and the ASReml R package (version 4.2) (Butler et al. 2023). The GWAS script included terms for correcting population structure and the kinship matrix (Korte et al. 2012). To determine population structure, principal component analysis was performed using the factoextra R package (Kassambara and Mundt 2020). Although the total number of SNPs was 4,285,827, only the SNPs with minor allele frequency >0.05 were considered for validation. Therefore, the Bonferroni threshold was determined by the −log_10_(*P*-value/# SNPs) for SNPs with minor allele frequency >0.05. This corresponded to −log_10_ (0.05/1,753,576) = 7.55. The broad-sense heritability was calculated using MVApp (Julkowska et al. 2019). The GWAPP web application is available at http://gwapp.gmi.oeaw.ac.at/ (Supplementary Table S2). The data followed a normal distribution and the threshold for significant SNPs was determined by using *P* < 0.1 with Bonferroni correction. The total number of SNPs was 204,741. Haplotype analysis was performed for *ExAD* using 3 significant SNPs found in the GWAS (Supplementary Data Set 1). The genotypes of the HapMap accessions were sorted according to the allele of each SNP, revealing 2 haplotype groups.

### Sequence comparisons among accessions

Sequence information for HapMap accessions was obtained from the 1001 Genome Project (Weigel and Mott 2009), available at http://signal.salk.edu/atg1001/3.0/gebrowser.php. Sequences were aligned with ClustalO (Supplementary Data Set 2), and the comparisons are presented in 3 plots including missing data, gaps, and similarity by gnuplot software package (Julkowska et al. 2016).

### T-DNA insertion line genotyping

Leaf material from 2-wk-old plants was collected for DNA isolation. Tissue was ground in liquid N_2_, incubated with Lysis buffer (2% SDS, 100 mm Tris pH 7.5, 10 mm EDTA) at 65°C for at least 20 min, and precipitated with ammonium acetate (3 m NH_4_Ac). Supernatants were collected and incubated with 2-propanol and spun at a max speed of 21,130 × *g* at room temperature. The DNA pellets were resuspended in water for PCR analysis. Primers and SALK identification numbers are listed in Supplementary Table S4.

### Gene expression analysis

For *ExAD* expression analysis in T-DNA mutants (*exad1-1* and *exad1-3*), seeds were germinated and grown on ½x MS, 0.5% sucrose, 0.1% MES (pH 5.8), and 1% Daishin (Duchefa) agar plates. Eight days after germination, seedlings were harvested, flash-frozen, lyophilized, and ball-milled in a mixer mill for further RNA extractions. Total RNA was extracted and purified from tissue samples homogenized in TRIPure (Sigma), followed by incubation with chloroform and spun at a max speed of 21,130 × *g*. The aqueous phase was transferred to a fresh RNase-free tube, followed by isopropanol purification and spun again at a max speed 21,130 × *g* for 10 min to collect the RNA pellet. The RNA pellet was washed by 70% ethanol and followed by DNase (Ambion) treatment, 1 unit of 10× DNase was used for 1–2 *µ*g RNA. cDNA synthesis was performed using the iScript cDNA synthesis kit (Bio-Rad) according to the manufacturer's instructions. Reverse transcription qPCR (RT-qPCR) was carried out using Bio-Rad CFX96 system. Relative expression was calculated using the reference gene *AT2G43770* (*MAC17*) and *AT2G28390* (*MON1*) individually, and the average was used. Values represent means ± SEs from 9 tubes from 3 plates, each tube containing 2 seedlings.

For *ExAD* expression analysis in the accessions of the 2 haplotypes, seeds were germinated and grown in ½x MS, 0.5% sucrose, and 0.1% MES (pH 5.8) in a flask, with shaking at 130 rpm under long-day conditions. Four days after germination, seedlings were transferred to a medium containing 0 or 100 mm NaCl for 48 h before harvesting. Seedlings were harvested, flash frozen, lyophilized, and ball-milled in a mixer mill. Approximately, 8 seedlings were pooled as 1 biological replicate and 5 biological replicates for each treatment, and each genotype was used. Total RNA was extracted and purified using a NZY Total RNA Isolation kit (NZYTech). cDNA synthesis kit (Bio-Rad) was performed using an iScript cDNA synthesis kit (Bio-Rad). qRT-PCR was carried out as described in https://academic.oup.com/plcell/advance-article/doi/10.1093/plcell/koad317/7492837 using a CFX Opus 384 Real-Time PCR System (Bio-Rad). Relative expression was calculated using the reference gene *AT2G43770*. Primers used for gene expression analysis are listed in Supplementary Table S4.

### Sequential extraction and CoMPP analysis

The 5-d-old Col-0, *exad1-1*, and *exad1-3* mutant seedlings were transferred to the treatments containing 0 mm NaCl (as control) or 100 mm NaCl for 48 h before sampling. Seedling samples were harvested, flash frozen, and lyophilized. The dried material was lyophilized and ball-milled in a mixer mill. The initial alcohol insoluble residue (AIR1) was removed by incubating the material first for 30 min in 80% ethanol at 70°C, and then for 30 min in 70% ethanol at 95°C, and finally in chloroform:methanol (1:1) for 5 min at room temperature before washing with acetone. Starch (AIR2) was then removed according to Møller et al. (2017). AIR was extracted sequentially first with 50 mm CDTA (Merck Darmstadt, Germany) followed by 4 m NaOH and 26.5 mm NaBH_4_ (Merck Darmstadt). Aliquots of the resulting fractions (primarily pectin and hemicelluloses) were dotted onto nitrocellulose (Amersham Cytiva, Protn 0.45 *µ*m NC Nitrocellulose Blotting Membrane 300 mm × 4 m) using a piezoelectric array printer (Marathon, Arrayjet, Edinburgh, United Kingdom) resulting in microarrays with extractions from all the samples. The microarrays were then probed with a selection of antibodies and carbohydrate-binding modules (CBMs). A list of the antibodies used can be found in Supplementary Table S5. The microarrays were blocked with skimmed milk (Applichem, A0830.1000. 5% skimmed milk), probed with CBMs or monoclonal antibodies specific to glycan epitopes, and then visualized using the appropriate secondary antibodies as described in Møller et al. (2017). Spot intensities were quantified from 256-step gray-scale scans of the microarrays utilizing array detection software (Array-Pro Analyzer v 6.3, Media Cybernetics, Rockville, Maryland, United States), and data are presented on a relative scale with 100 assigned to the most intense spot. The extracted CoMPP fractions were dialyzed against demineralized water using Visking 12–14 kDa dialysis tubing (45 mm) prior to the determination of the monosaccharide composition.

### JIM11 dot-blot assay

The 5-d-old seedlings were transferred to treatments of 0 or 100 mm NaCl for 48 h before harvesting. Samples were flash-frozen, lyophilized, and ball-milled in a mixer mill. At least 3 biological replicates were used for each treatment and for each genotype used, including 3 replicates for the 0 mm NaCl treatment and 3 replicates for the 100 mm NaCl. Total proteins were extracted in 0.5 m Tris–HCl (pH 6.8), 0.037 g/L Na_2_ EDTA•2H_2_O, 2% SDS. Samples were heated to 98°C for 3 min. After a short centrifugation (15 min, 13,000 × *g*), supernatants were collected, and total proteins were quantified using the Bradford assay (Bio-Rad #5000205). A 25 *µ*g of total proteins were spotted onto a nitrocellulose membrane (0.45 *μ*m Bio-Rad). Membranes were dried overnight at room temperature. Blocking [5% BSA in 1× phosphate-buffered saline (PBS; Bio-Rad #1610780)] was performed for 1 h. Membranes were incubated for 1 h with primary antibody anti-JIM11 (Agrisera, AS21 4692) (1:500, 5% BSA in PBS). Membranes were incubated with secondary antibodies (rat anti-IgG) (1:6000, 5% BSA in PBS) and after 3 washing steps (1× PBS, 0.1% Tween), chemiluminescence was induced using the Clarity Western ECL Substrate (Bio-Rad) and detected with a ChemiDoc imaging system (Bio-Rad).

### Transmission electron microscopy

The 4-d-old seedlings were transferred to agar plates supplemented with 100 mm NaCl or 0 mm (as control) for 48 h before analysis using the SITA system. Specimens were cut into small pieces of about 1 mm^3^ at the selected zones (elongation zone and maturation zone) before fixation. Root pieces were fixed by submerging and incubating in 2.5% glutaralehyde + 2% paraformaldehyde in 0.1 m phosphate/citrate buffer (pH 7.2) for at least 1 h at room temperature. Specimens were washed by removing the present reagent and adding the 0.1 m phosphate/citrate buffer again for at least 6 times for 10 min. 1% osmium tetroxide in 0.1 m phosphate/citrate (pH 7.2) buffer was added and samples were incubated for 1 h at room temperature. Specimens were washed at least 3 times for 10 min each in Milli-Q water. Next, specimens were dehydrated in a graded ethanol series: 30% for 5 min, 50% for 5 min, 70% for 5 min, 80% for 5 min, 90% for 5 min, 96% for 5 min, 100% for 10 min, and then infiltrated with resin (Spurr's resin) 1:2 resin:ethanol for 30 min, 1:1 resin:ethanol for 30 min, 2:1 resin:ethanol for 30 min, and 100% resin for 60 min to overnight at room temperature (Spurr’s kit Catalog number 14300). A Leica EM RAPID microtome was used for trimming (pre-sectioning) of the sample and a Leica Ultramicrotome U7 was used for sectioning and attaining the sections. Sections were viewed using a JEOL 1400 TEM operating at 120 kV.

### Cell wall analysis

Five-day-old Col-0, *exad1-1*, and *exad1-3* and Col-0, *xeg113-1*, and *xeg113-2* seedlings were treated with 0 or 100 mm NaCl and harvested after 48 h of salt treatment. Arabidopsis seedlings were lyophilized and ball-milled in a Retsch mixer mill. All samples were extracted 3 times with 70% ethanol and 3 times with 1:1 (v:v) chloroform:methanol in the Retsch mill, washed with acetone, and dried in a vacuum concentrator. The AIR was weighed out in 2 mL screw cap tubes and used for extraction of neutral cell wall sugars and cellulose as described in Yeats et al. (2016). High-performance anion-exchange chromatography with pulsed amperometric detection was performed on a biocompatible Knauer Azura HPLC system, equipped with an Antec Decade Elite SenCell detector. Monosaccharides were separated on a Thermo Fisher Dionex CarboPac PA20 column with solvents (A) water, (B) 10 mm NaOH, and (C) 700 mm NaOH at a flow rate of 0.4 mL/min as follows: 0 to 25 min: 20% B, 25 to 28 min: 20% to 0% B, 0% to 70% C, 28 to 33 min: 70% C, 33 to 35 min: 70% to 100% C, 35 to 38 min: 100% C, 38 to 42 min: 0% to 20% B, 100% to 0% C, and 42 to 60 min: 20% B.

### Synthesis of the cell wall mechano-probe

The cell wall mechano-probe for FLIM analysis, as detailed in Michels et al. (2022), was employed in this study. The chemical synthesis method differed from the previous publication and its procedure is outlined below. Step 1: Synthesis of 4-(azidomethyl)benzaldehyde. 4-(bromomethyl)benzaldehyde (5 g, 25 mmol) and NaN_3_ (2.5 g, 38 mmol) were added to 50 mL of dimethylformamide (DMF) in a round bottom flask. The solution was left stirring at 60°C for 1.5 h. After cooling, the solution was diluted with 250 mL of ethyl acetate and washed twice with 250 mL of water. The resulting organic layer was dried with MgSO_4_, filtered, concentrated, and dried under vacuum to yield 4-(azidomethyl)benzaldehyde (3.76 g, 93% yield). 1H NMR (400 mHz, CDCl_3_) δ 10.02 (s, 1H), 7.90 (d, J = 8.1 Hz, 2H), 7.49 (d, J = 8.5 Hz, 2H), 4.45 (s, 2H).

Step 2: Synthesis of N_3_-BODIPY mechano-probe. To a 1-L triple-neck round bottom flask 4-(azidomethyl)benzaldehyde (2.0 g, 12.41 mmol) was added, followed by 500 mL of anhydrous dichloromethane and 2-methylpyrrole (2.092 mL, 24.82 mmol). After sparging the solution with N_2_ gas for 30 min, trifluoroacetic acid (500 *µ*L, 6.2 mmol) was added. The reaction mixture was left stirring for 2 h, followed by the addition of 2,3-dichloro-5,6-dicyano-1,4-benzoquinone (2.82 g, 12.41 mmol) after which the mixture was sparged with N_2_ gas for 10 min followed by 20 min of stirring. Finally, di-isopropylethylamine (15.1 mL, 86.7 mmol) and boron trifluoride diethyl etherate (15.3 mL, 124 mmol) were added, after which the mixture was left stirring for 24 h under N_2_. A 2:3 hexane:ethyl acetate was added to the reaction medium, after which dichloromethane was removed under reduced pressure. After purification on silica (2:3 hexane:ethyl acetate), the product was isolated as a red, crystalline solid (1.07 g, 25% yield). 1H NMR (400 mHz, CDCl_3_) δ 7.52 (d, J = 8.1 Hz, 2H), 7.43 (d, J = 8.1 Hz, 2H), 6.69 (d, J = 4.1 Hz, 2H), 6.27 (d, J = 4.1 Hz, 2H), 4.46 (s, 2H), 2.65 (s, 6H).

Step 3: Synthesis of 1,4,8,11-tetra(prop-2-yn-1-yl)-1,4,8,11-tetraazacyclotetradecane based on Counsell et al. (2016). To a solution of 1,4,8,11-tetraazacyclotetradecane (500 mg, 2.49 mmol) in 5 mL of acetonitrile, 5 mL of 1 m NaOH was added followed by 1.1 mL 80% propargyl bromide solution (in toluene, 10.2 mmol). The mixture was left overnight with very gentle stirring. The precipitate was collected, washed with hexane, and dried under vacuum to yield the desired product (236 mg, 27% yield). 1H NMR (400 mHz, CDCl_3_) δ 3.44 (s, 8H), 2.62 (s, 16H), 2.17 (s, 4H), 1.77 (s, 4H), 1.61 (s, 4H).

Step 4: Synthesis of (2-azidoethyl)trimethylammonium bromide (based on Francavilla et al. 2009). To 30 mL of DMF, (2-bromoethyl)trimethylammonium bromide (1 g, 4.05 mmol) and NaN_3_ (0.65 g, 10.1 mmol) were added. After stirring overnight, the mixture was concentrated and diluted with tetrahydrofuran, after which the resulting precipitate was collected and dried to yield (2-azidoethyl)trimethylammonium bromide (480 mg, 57% yield). 1H NMR (400 MHz, D_2_O) δ 4.01 (s, 2H), 3.69–3.60 (m, 2H), 3.25 (s, 9H).

Step 5: Synthesis of the cell wall mechano-probe (based on Lipshutz and Taft 2006). To a 0.5 mL microwave vial N3-BODIPY (60 mg, 0.17 mmol), 1,4,8,11-tetra(prop-2-yn-1-yl)-1,4,8,11-tetraazacyclotetradecane (60 and 0.17 mg) and Cu-impregnated activated charcoal (17 mg) were combined. Then, 0.4 mL 1,4-dioxane was added and the mixture was heated to 150°C for 20 min under continuous stirring in a Biotage initiator + system (Biotage initiator + fourth generation). After filtration (Celite), the reaction mixture was dried. From the resulting solid, 39 mg was added to a fresh 0.5 mL microwave vial, after which (2-azidoethyl)trimethylammonium bromide (87 mg, 0.42 mmol) and Cu-impregnated activated charcoal (17 mg) were added. 0.25 mL Milli-Q water and 0.25 mL 1,4-dioxane were added, and the mixture was heated to 150°C for 20 min under continuous stirring in a Biotage initiator + system. The resulting mixture was filtered through a 0.5 C18 SPE column (Screening Devices, Amersfoort, the Netherlands). A 6.6 mg product was isolated as a red crystalline solid and was used without any additional purification.

### FLIM imaging and analysis

The 5-d-old Col-0, *exad1-1*, and *exad1-3* seedlings were treated with 100 mm NaCl for 48 h. Analyses were performed using a Leica TCS SP8 inverted scanning confocal microscope coupled with a Becker-Hickl SPC830 time-correlated single photon counting module for FLIM image acquisition. A Leica TCS SP5 X pulsed white light laser with a repetition rate of 40 MHz and an excitation wavelength of 488 nm was used as a laser source. Imaging was performed with a 63× 1.2 NA water immersion objective with a 256 × 256 pixel resolution. A line scanning speed of 400 Hz was used, and the emission was collected between 500 and 550 nm onto a Leica HyD SMD hybrid photodetector.

### Statistical analysis and R scripts

Statistical analyses in this study were performed in R. The normal distributions of phenotypic data were fitted in a linear model and 2-way ANOVA was performed followed with contrasts post hoc test. Treatments and genotypes were selected as the factors, and post hoc contrasts comparison was performed with the nlme (multcomp) package in R. For comparison between 2 groups, a Student’s *t*-test was performed. Details of the statistical analyses for all figures are listed in Supplementary Data Set 3. The scripts for analyzing the data in this study are available at https://github.com/YutaoYutao/S-root.

## Accession numbers

Sequence data from this article can be found in the GenBank/EMBL data libraries under accession numbers: *ExAD* (AT3G57630): *exad1-1* (SAIL_843_G12; N862789) and *exad1-3* (SALK_204414C; N693167) and *Xeg113* (AT2G35610): *xeg113-1* (SALK_151754), *xeg113-2* (SALK_066991).

## Supplementary Material

koae135_Supplementary_Data

## Data Availability

The data underlying this article are available in the article and in its online supplementary material.

## Supplementary data

The following materials are available in the online version of this article.

**Supplementary Figure S1.** RTD in time-lapse SITA is modulated by NaCl treatment.

**Supplementary Figure S2.** Natural variation observed in RTD of 20 Arabidopsis accessions analyzed with the SITA time-lapse system.

**Supplementary Figure S3.** Candidate loci for different traits in SITA mapped by GWAS using 345 Arabidopsis accessions.

**Supplementary Figure S4.** QQ-plots for traits in SITA mapped by GWAS using 345 Arabidopsis accessions.

**Supplementary Figure S5.** Natural variation analysis of ExAD gene.

**Supplementary Figure S6.** Phenotypical characterization of *exad* and *xeg113* mutant lines.

**Supplementary Figure S7.** Heatmap of CoMPP spot signals.

**Supplementary Figure S8.** JIM1 signals and cell wall composition analysis in control vs. salt-treated Col-0, *exad*, and *xeg113* mutant seedlings.

**Supplementary Figure S9.** Cell wall thickness and porosity analyzed in control vs. salt-treated Col-0 and *exad* mutant lines.

**Supplementary Table S1.** List of accessions used in this study.

**Supplementary Table S2.** List of candidate loci identified with GWAS via GWAPP web tool.

**Supplementary Table S3.** List of candidate loci identified with GWAS via R package.

**Supplementary Table S4.** Primers used in this study.

**Supplementary Table S5.** Antibodies used in the CoMPP analysis.

**Supplementary Table S6.** Antibodies concentrations and signals in the mutants and conditions used in this study.

**Supplementary Data Set 1.** List of root vector angle (RVA^N-C^) at 23 h in two haplotypes of ExAD.

**Supplementary Data Set 2.** Alignment of sequence comparisons among accessions (FASTA).

**Supplementary Data Set 3.** Individual data points and statistics of data presented in the figures.

**Supplementary Movie S1.** Root response of Col-0 seedlings in time-lapse SITA is modulated specifically by NaCl treatment.

## Dive Curated Terms

The following phenotypic, genotypic, and functional terms are of significance to the work described in this paper:

LRX1 Gramene: AT1G12040
LRX1 Araport: AT1G12040

LRX2 Gramene: AT1G62440
LRX2 Araport: AT1G62440

AT2G43770 Gramene: AT2G43770
AT2G43770 Araport: AT2G43770

AT2G28390 Gramene: AT2G28390
AT2G28390 Araport: AT2G28390

AT3G57630 Gramene: AT3G57630
AT3G57630 Araport: AT3G57630
