# Supplementary material for: Arabinosylation of cell wall extensin is required for the directional response to salinity in roots
Source: Plant Cell. 2024 May 1;36(9):3328–43. doi: 10.1093/plcell/koae135 (PMC11371136; doi:10.1093/plcell/koae135)
Supplement: koae135_Supplementary_Data [file koae135_supplementary_data.zip › TPC2023BR01176DR1_Supplementary_Data.pdf]

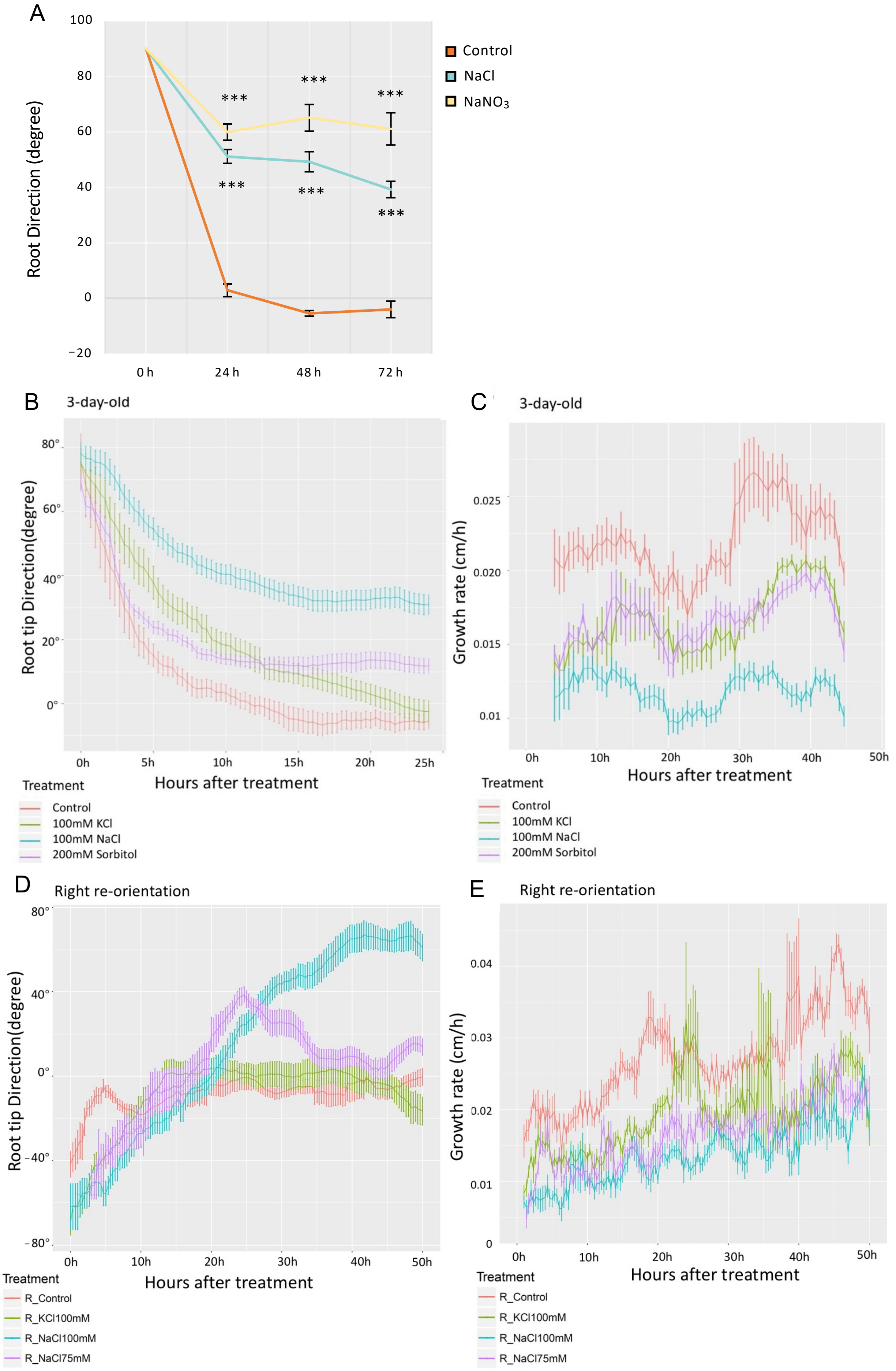

Supplementary Figure S1. **Root Tip direction (RTD) in time-lapse SITA is modulated by NaCl treatment (Supports Figure 1).** **A** Root angle was analyzed in SITA in Col-0 seedlings treated with (control, 100 mM NaCl, 100 mM $NaNO_3$) at 24, 48 and 72 h. Comparisons between control and NaCl or control and $NaNO_3$ have been performed by using multiple paired T-tests coupled with Independent Sample Kruskal-Wallis test. ***, $P < 0.001$. **B** and **C**, quantification of RTD **(B)** and growth rate **(C)** of 3-day-old Col-0 seedlings under different treatments (control, 100 mM NaCl, 100 mM KCl and 200 mM sorbitol) over 24 h. Values represent means ± SE of 40 seedlings from 2 plates. **D** and **E**, quantification of RTD **(E)** and growth rate **(E)** of 3-day-old Col-0 seedlings under different treatments (control, 100 mM NaCl, 100 mM KCl and 200 mM Sorbitol) over 24 h. Values represent means ± SE of 35 seedlings from 1 plate. C and D are from 2 independent experiments.

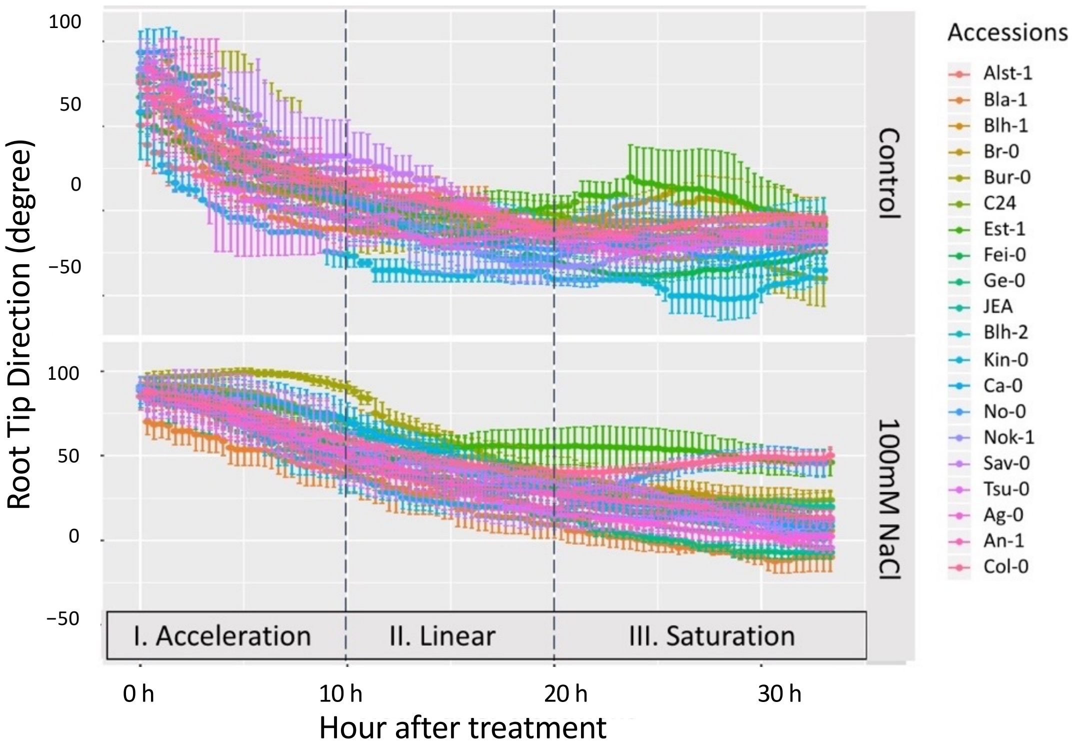

SupplementalSu Figure S2. Natural variation observed in root tip direction (RTD) of 20 Arabidopsis accessions analyzed with the SITA time-lapse system (Supports Figure 2). Four-day-old seedlings of 20 Arabidopsis accessions were transferred to agar plates with or without 100 mM NaCl. The different colors represent the analyzed accessions. Dynamic RTD responses were followed in three phases that are indicated with dash lines: I) Acceleration, II) Linear and III) Saturation. Values represent means ± SE of 5 seedlings. Data are representative of two independent experiments.

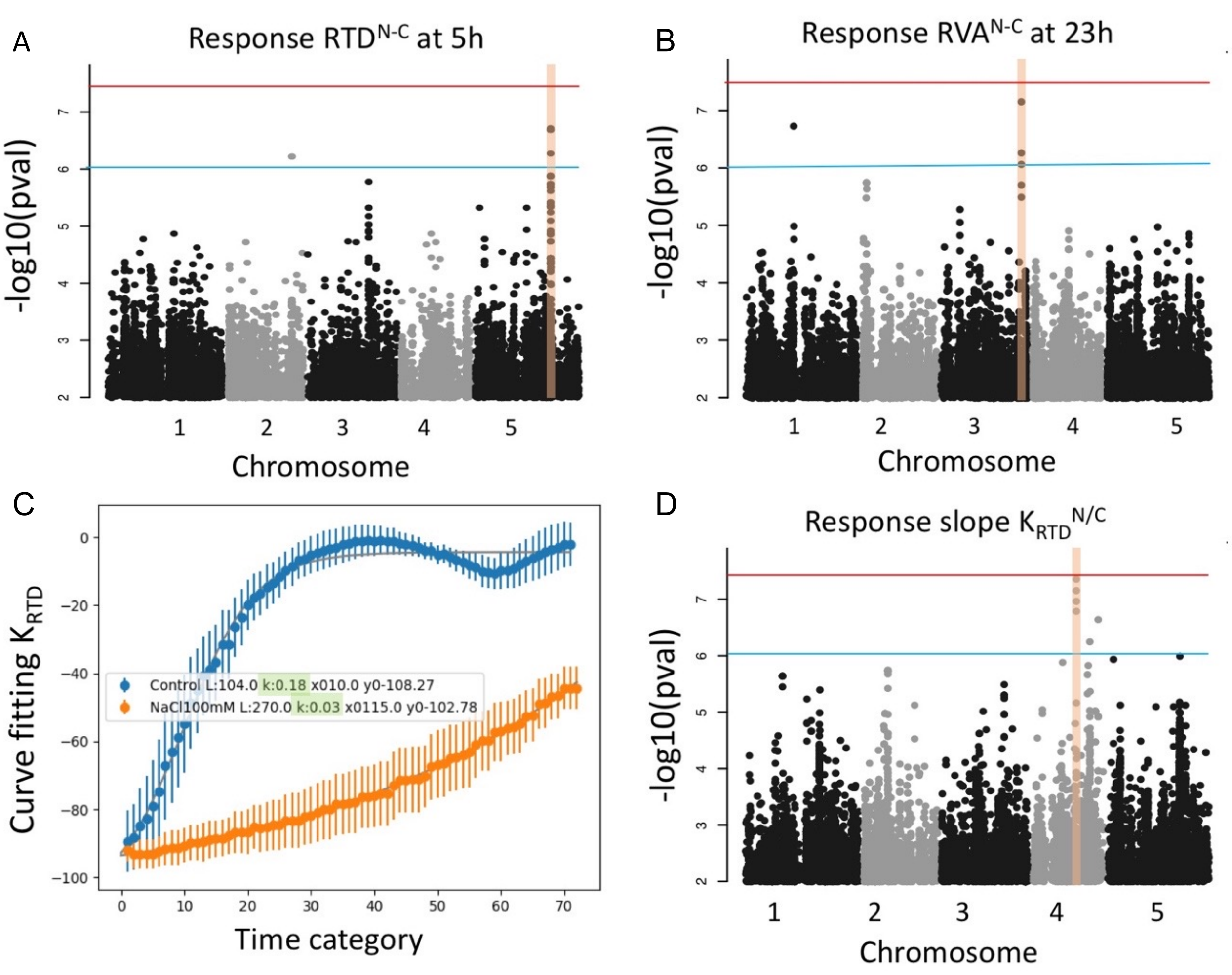

Supplementary Figure S3. Candidate loci for different traits in SITA mapped by GWAS using 345 Arabidopsis accessions (Supports Figure 2). Manhattan plots illustrating the association of single nucleotide polymorphisms (SNPs) with two root response traits, namely, Response Root Tip Direction at 5 hours ($RTD^{N\text{-}C}$) in panel **A** and Response Root Vector Angle at 23 hours ($RAV^{N\text{-}C}$) in panel **B,** $RTD^{N\text{-}C}$ and $RAV^{N\text{-}C}$ denote the differences in Root Tip Directions (RTDs) and Root Vector Angles (RAVs), respectively, between salt-stressed and control conditions. **C,** depicts the calculated K_{RTD} (Kinetic Relative Root Tip Direction) for the Col-0 accession, derived from a 4-parameter logistic regression (4PL) model fit to relative RTD data across various accessions, describing the rate of relative root response under salt stress. Python was employed for parameter calculation through curve fitting (script available upon request). The black boxes highlight the 'k' values, representing the KRTD parameter, for both control and NaCl treatments as determined by the model. **D,** Manhattan plot for the SNPs associated with $K_{RTD}{}^{N/C}$. $K^{N/C}$ is the response in fitted rate of exponential decay of RTDs, $K_{RTD}{}^{N/C}$ represents K_{RTD} under salt condition divided by that under control condition. In each Manhattan plot, different chromosomes are indicated by gray or black color as X-axis; the Y-axis shows LOD score. All *loci* above the arbitrary threshold of LOD=6.0 (bule line) were highlighted in orange and listed in Table S2. Bonferroni threshold = 7.55 (red line) was determined by the $-\log_{10}$(p-value / # SNPs) for SNPs with minor allele frequency > 0.05.

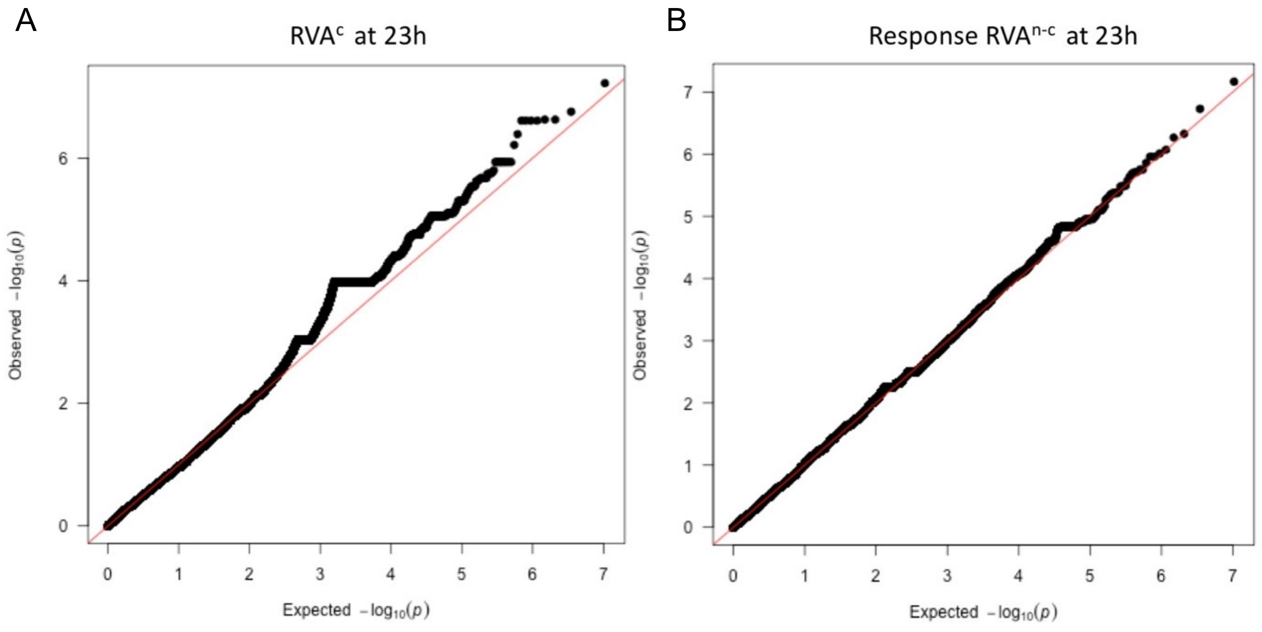

Supplementary Figure S4. QQ-plots for traits in SITA mapped by GWAS using 345 Arabidopsis accessions (Supports Figure 2). QQ-plots for root vector angle under control condition (RAV^{C}) **(A)** and root response root vector angle ($RAV^{N\text{-}C}$) at 23 h **(B)**.

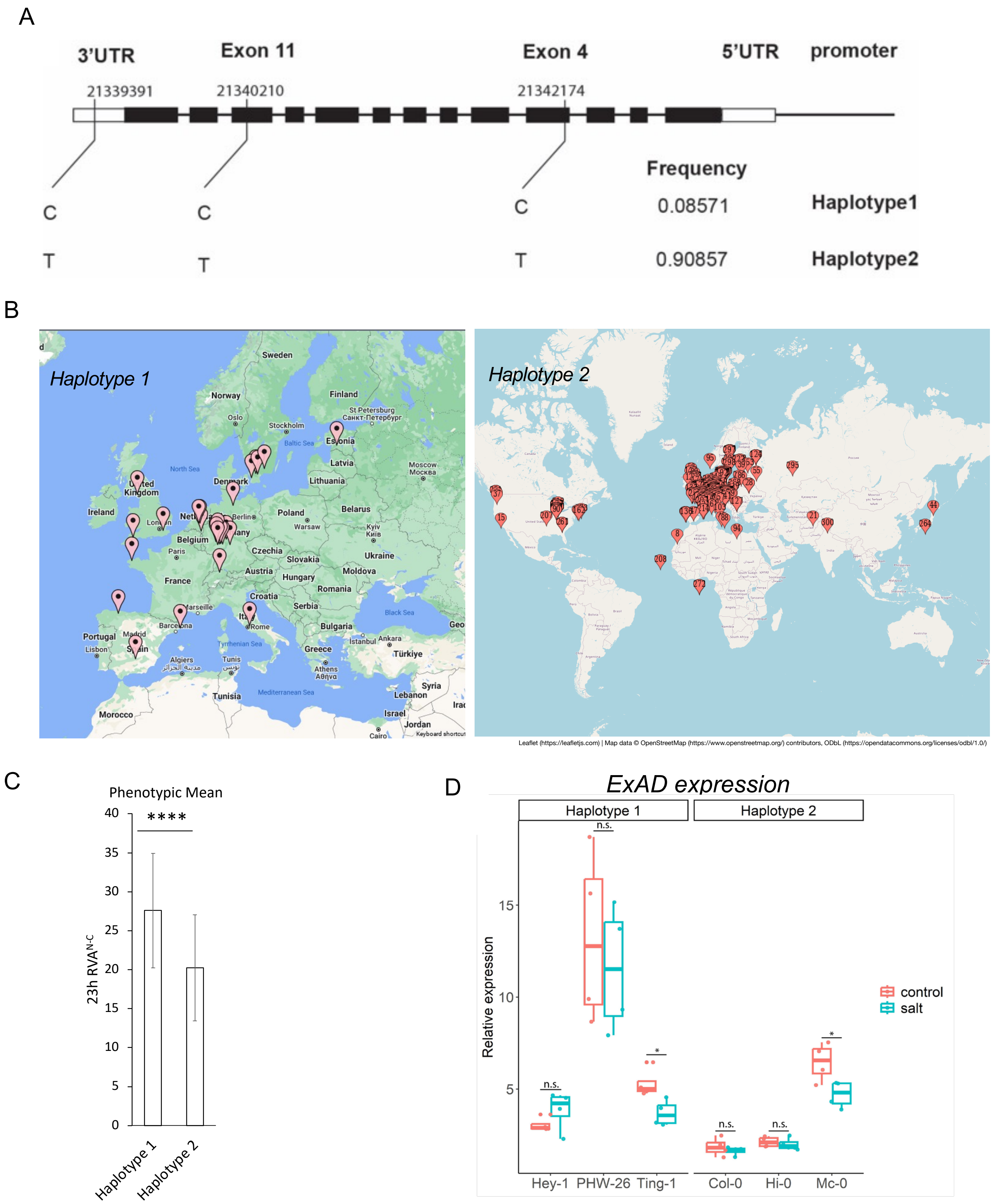

Supplementary Figure S5. Natural variation analysis of ExAD gene (Supports Figure 2). A, Two haplotypes were identified for ExAD (*AT3G57630*) using 3 significant SNPs found in GWAS (SNP at position 21339391, 21340210 and 21342174) based on genomic variations and haplotype frequency in Arabidopsis Hapmap accessions. **B,** The geographic distribution of the accessions used in this study, divided into two haplotypes, has been depicted on maps generated with https://maps.co/gis/ (haplotype 1) or www.mapcustomizer.com (haplotype 2) using the original coordinates from https://arapheno.1001genomes.org/accessions/. **C**, Average of root vector angle values (RVA^{N-C}) at 23 h under salt condition subtracted by RVA under control condition in two haplotypes of ExAD. Data represent means ± SD. Five biological replicates (each seedling as 1 replicate) were used for calculation. **D**, Expression of ExAD under salt (100 mM NaCl for 48 h) and control conditions in tested accessions from two haplotypes relative to housekeeping gene (*AT2G43770*). Data represent means ± SE. Five biological replicates were used (approximately 8 seedlings were pooled as 1 replicate). Statistical analyses in (**C**) and (**D**) were determined using Student's T-Test. F-test was performed to check equal variances of samples. Standard box plots elements are minimum, Q1 (first Quartile), median, Q3(third Quartile), and maximum. Significant differences were determined by Two-sample T-Test with equal variance otherwise Welch's t-test (*, p<0.05; ****,p<0.001; p>0.05, n.s.).

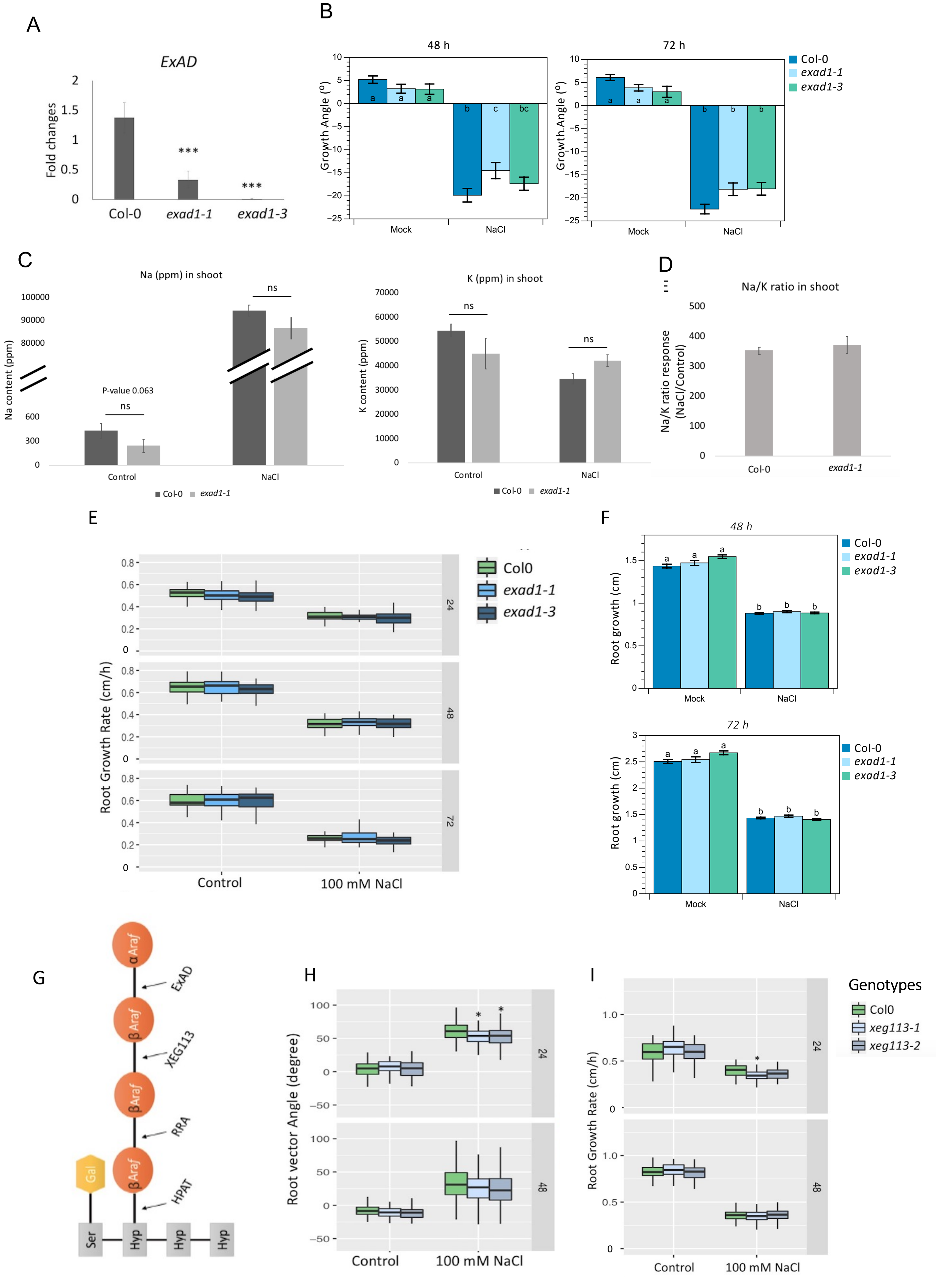
A
ExAD
Fold changes
2
1.5
1
0.5
0
***
***
Col-0
exad1-1
exad1-3
B
48 h
72 h
Growth Angle (º)
Mock
NaCl
Col-0
exad1-1
exad1-3
C
Na (ppm) in shoot
Na content (ppm)
100000
90000
80000
600
300
0
P-value 0.063
ns
Control
NaCl
Col-0
exad1-1
K (ppm) in shoot
K content (ppm)
70000
60000
50000
40000
30000
20000
10000
0
D
Na/K ratio in shoot
Na/K ratio response (NaCl/Control)
500
400
300
200
100
0
E
Root Growth Rate (cm/h)
Col0
exad1-1
exad1-3
24
48
72
Control
100 mM NaCl
F
48 h
72 h
Root growth (cm)
Mock
NaCl
G
αAraf
βAraf
Gal
ExAD
XEG113
RRA
HPAT
Ser
Hyp
H
Root vector Angle (degree)
100
50
0
−50
I
Root Growth Rate (cm/h)
Genotypes
Col0
xeg113-1
xeg113-2

Figure S6 (Supports Figure 3). A. Expression of *ExAD* in 8-day-old *Arabidopsis* seedlings of Col-0, *exad1-1* and *exad1-3* mutants measured through qRT-PCR. Primers used for the analysis are reported in Table S4. Values represent the average ± SE of nine biological replicates, each containing at least 5 seedlings. Statistical analysis was done with Student's T-test. **B**, Absolute root angle values of experiment in Figure 3C was performed as in (Galvan-Ampudia et al., 2013) in 5-day old Col-0, *exad1-1* and *exad1-3* seedlings. Seedlings were transferred to agar plates with/without NaCl gradient and root growth in cm was measured after 48 and 72 h from the transferring time point. Values represent means ± SE from 48 seedlings Seedlings were treated for 48 and 72 h and root bending analysed after plate scanning with Smartroot (Fiji Plugin). Non-parametric analysis (Shapiro-Wilk test, $p < 0.05$) was performed. Kruskal-Wallis test was used to detect group differences, and Dunn's test determined specific significant differences ($p < 0.05$). **C,** Quantification of Na^+ content (ppm) and K^+ content (ppm), and Na+/K+ ratio (NaCl treatment/Control treatment) (**D)** was performed in shoots of Col-0 and the *exad1-1* mutant. Three-week-old plants were grown hydroponically and transferred to liquid media containing either NaCl (150 mM) or control (0 mM) for 4 days. The shoots were harvested for ion measurement, and data were normalized by fresh weight. Values represent means ± SE from 4 biological replicates, each containing 2 shoots from independent plants. Statistical analysis was performed using Student's T-test. No significant differences (ns) were detected. **E,** Quantification of root growth rate of Col-0, *exad1-1* and *exad1-3* mutants in SITA. Four-day-old seedlings were transferred to agar plates with or without 100 mM NaCl for 24, 48 and 72 h. Values represent means ± SE from 50 seedlings from 10 plates, each plate containing 5 seedlings. Data are representative of two independent experiments. Statistical analysis was performed by using two-way ANOVA with contrasts post-hoc. **F,** Quantification of root growth rate of Col-0, *exad1-1* and *exad1-3* mutants, in halotropism (Figure 3C) is expressed in cm. Treatment and statistical analysis were performed as in (B). **G,** Schematic diagram of extensin repeat side-chain Hyp-$Araf_{1\text{-}4}$ and extensin arabinosylation enzymes. Serine is substituted with galactose and the hydroxyprolines (Hyps) are substituted with β- arabinofuranoses (β-Araf) and α-arabinofuranoses (α-Araf). HYDROXYPROLINE ARABINOSYLTRANSFERASES 1-3 (HPAT1-3) adds the first arabinose (Hyp-$Araf_1$) to Hydroxyprolines (Hyps) (Ogawa-Ohnishi et al., 2013). REDUCED RESIDUAL ARABINOSE 1-3 (RRA1-3) adds the second arabinose residue (Hyp-$Araf_2$) (Egelund et al., 2007; Velasquez et al., 2011). XYLOGLUCAN ENDOGLUCANASE 113 (XEG113) adds the third arabinose residue (Hyp-$Araf_3$) (Gille et al., 2009). EXTENSIN DEFICIENT ARABINOSE (ExAD), ExAD adds the fourth arabinose residue (Hyp-$Araf_4$) on the extensin repeat **H,** Quantification of root vector angle of Col0, *xeg113-1* and *xeg113-2* mutants in SITA. Four-day-old seedlings transferred to agar plate with or without 100 mM NaCl for 24 and 48 h. Values represent means ± SE from 7 biological replicates (plates) and 10 technical replicates (seedlings). Error bars represent SE of the mean. Statistical analysis was done using two-way ANOVA with contrasts post-hoc. Asterisks indicate statistically significant differences compared to Col-0 (p-values ***$P< 0.001$; **$P < 0.01$; *$P < 0.05$). **I,** Quantification of root growth rate of Col-0, *xeg113-1* and *xeg113-2* mutants in SITA. Four-day-old seedlings transferred to agar plate with or without 100 mM NaCl for 24 and 48 h. Values represent the means ± SE of 7 biological replicates (plates) and 10 technical replicates (seedlings). Statistical analysis was done using two-way ANOVA with contrasts post-hoc. Asterisks indicate statistically significant differences compared to Col-0 (***$P < 0.001$; **$P < 0.01$; *$P < 0.05$).

Extraction	Genotypes	Treatment	JIM5	JIM7	LM5	LM6	LM7	LM8	LM13	LM16	LM18	LM19	LM20	Inra RU1	Inra RU2	LM15	LM25	LM10	LM11	LM28	LM23	BS400-4	LM21	LM22	CBM3a	LM1	LM3	JIM11	JIM12	JIM19	JIM20	LM2	LM14	JIM4	JIM8	JIM13	JIM14	JIM15	JIM16	JIM17	MAC207	anti-rat	anti-mouse	anti-his
CDTA Fraction	Col-0	Control (0mM)	68	100	0	24	0	0	0	0	57	76	86	45	59	8	12	0	0	0	0	0	0	0	0	0	69	39	37	0	32	31	43	0	0	16	0	0	21	90	20	0	0	0
	Col-0	Control (0mM)	46	75	0	25	0	0	0	0	40	55	71	37	48	9	13	0	0	0	0	0	0	0	0	0	64	35	44	0	35	29	40	0	0	17	0	0	19	65	23	0	0	0
	Col-0	Control (0mM)	55	98	0	20	0	0	0	0	48	68	95	41	54	6	10	0	0	0	0	0	0	0	0	0	48	32	46	0	33	30	39	0	0	13	0	0	13	89	20	0	0	0
	exad1-1	Control (0mM)	29	25	0	14	0	0	0	0	49	67	0	32	37	11	9	0	0	0	0	0	0	0	0	0	37	0	34	0	27	20	26	0	0	9	0	0	9	29	13	0	0	0
	exad1-1	Control (0mM)	30	26	0	9	0	0	0	0	50	71	0	25	26	8	7	0	0	0	0	0	0	0	0	0	30	0	33	0	30	17	20	0	0	6	0	0	6	27	8	0	0	0
	exad1-1	Control (0mM)	30	30	0	13	0	0	0	0	52	71	0	34	36	9	10	0	0	0	0	0	0	0	0	0	34	0	36	0	28	18	19	0	0	7	0	0	10	30	9	0	0	0
	exad1-3	Control (0mM)	58	72	0	17	0	0	0	0	63	83	45	34	39	7	9	0	0	0	0	0	0	0	0	0	41	0	41	0	33	20	20	0	0	5	0	0	9	62	8	0	0	0
	exad1-3	Control (0mM)	56	73	0	28	0	0	0	0	53	72	52	38	48	9	14	0	0	0	0	0	0	0	0	0	83	0	52	0	42	32	36	0	0	13	0	0	22	66	20	0	0	0
	exad1-3	Control (0mM)	71	98	0	18	0	0	0	0	56	74	86	36	42	10	12	0	0	0	0	0	0	0	0	0	57	0	50	0	39	28	34	0	0	11	0	0	12	86	16	0	0	0
	Col-0	NaCl (100mM)	41	40	0	29	0	0	0	0	47	72	0	27	34	0	8	0	0	7	0	0	0	0	0	0	48	29	30	0	28	25	31	0	0	8	0	0	19	34	23	0	0	0
	Col-0	NaCl (100mM)	35	39	0	25	0	0	0	0	42	62	0	23	25	6	7	0	0	5	0	0	0	0	0	0	43	35	29	0	33	24	30	0	0	9	0	0	21	35	23	0	0	0
	Col-0	NaCl (100mM)	32	40	0	29	0	0	0	0	43	58	0	21	24	5	7	0	0	6	0	0	0	0	0	0	59	45	36	0	43	29	36	0	0	12	0	0	25	35	29	0	0	0
	exad1-1	NaCl (100mM)	41	57	0	25	0	0	0	0	43	64	29	18	15	6	6	0	0	0	0	0	0	0	0	0	58	0	37	0	36	24	25	0	0	0	0	0	13	53	19	0	0	0
	exad1-1	NaCl (100mM)	36	46	0	27	0	0	0	0	45	61	17	17	15	6	6	0	0	0	0	0	0	0	0	0	57	0	30	0	31	22	25	0	0	0	0	0	11	40	19	0	0	0
	exad1-1	NaCl (100mM)	49	65	0	29	0	0	0	0	56	76	40	24	23	5	8	0	0	0	0	0	0	0	0	0	61	0	33	0	32	23	28	0	0	0	0	0	12	50	22	0	0	0
	exad1-3	NaCl (100mM)	34	27	0	29	0	0	0	0	56	69	0	18	13	7	7	0	0	0	0	0	0	0	0	0	57	0	33	0	32	25	27	0	0	6	0	0	15	26	21	0	0	0
	exad1-3	NaCl (100mM)	34	28	0	31	0	0	0	0	55	69	0	12	9	6	6	0	0	0	0	0	0	0	0	0	62	0	35	0	37	28	28	0	0	6	0	0	15	24	22	0	0	0
	exad1-3	NaCl (100mM)	39	36	0	38	0	0	0	0	62	76	0	24	27	8	10	0	0	8	0	0	0	0	0	0	91	0	50	0	46	36	36	0	0	11	0	0	29	41	33	0	0	0
NaOH-Fraction	Col-0	Control (0mM)	0	0	0	0	0	0	0	0	0	16	0	8	10	44	36	0	0	19	0	0	9	0	0	0	60	35	35	0	28	12	21	0	0	0	0	0	5	0	6	0	0	0
	Col-0	Control (0mM)	0	0	0	0	0	0	0	0	0	15	0	8	9	28	30	0	0	16	0	0	0	0	0	0	67	33	37	0	34	15	19	0	0	0	0	0	9	0	5	0	0	0
	Col-0	Control (0mM)	0	0	0	0	0	0	0	0	0	16	0	8	8	31	37	0	0	16	0	0	0	0	0	0	66	30	36	0	29	14	11	0	0	0	0	0	8	0	0	0	0	0
	exad1-1	Control (0mM)	0	0	0	0	0	0	0	0	0	12	0	0	0	39	32	0	0	16	0	0	0	0	0	0	36	26	27	0	31	14	11	0	0	0	0	0	13	0	0	0	0	0
	exad1-1	Control (0mM)	0	0	0	0	0	0	0	0	0	11	0	0	0	45	33	0	0	14	0	0	0	0	0	0	21	0	19	0	17	6	7	0	0	0	0	0	0	0	0	0	0	0
	exad1-1	Control (0mM)	0	0	0	0	0	0	0	0	0	12	0	0	0	32	26	0	0	16	0	0	0	0	0	0	37	0	29	0	26	10	12	0	0	0	0	0	0	0	0	0	0	0
	exad1-3	Control (0mM)	0	0	0	0	0	0	0	0	0	10	0	0	0	34	29	0	0	12	0	0	0	0	0	0	25	0	24	0	22	9	10	0	0	0	0	0	0	0	0	0	0	0
	exad1-3	Control (0mM)	0	0	0	0	0	0	0	0	0	17	0	7	0	47	38	0	28	28	0	0	7	0	0	0	32	0	23	0	17	6	6	0	0	0	0	0	0	0	0	0	0	0
	exad1-3	Control (0mM)	0	0	0	0	0	0	0	0	0	14	0	0	0	52	45	0	0	14	0	0	0	0	0	0	38	0	29	0	26	10	8	0	0	0	0	0	0	0	0	0	0	0
	Col-0	NaCl (100mM)	0	0	0	6	0	0	0	0	0	20	0	6	5	30	37	0	6	26	0	0	0	0	0	0	51	11	30	0	0	0	0	0	0	0	0	0	0	0	0	0	0	0
	Col-0	NaCl (100mM)	0	0	0	5	0	0	0	0	0	19	0	6	0	33	36	0	7	26	0	0	0	0	0	0	55	14	31	0	0	0	0	0	0	0	0	0	0	0	0	0	0	0
	Col-0	NaCl (100mM)	0	0	0	5	0	0	0	0	0	18	0	6	6	35	36	0	0	26	0	0	0	0	0	0	54	11	32	0	0	0	0	0	0	0	0	0	0	0	0	0	0	0
	exad1-1	NaCl (100mM)	0	0	0	9	0	0	0	0	0	15	0	0	0	51	40	0	12	28	0	0	0	0	0	0	21	0	17	0	9	0	0	0	0	0	0	0	0	0	0	0	0	0
	exad1-1	NaCl (100mM)	0	0	0	9	0	0	0	0	0	15	0	0	0	51	42	0	6	26	0	0	0	0	0	0	21	0	18	0	8	0	0	0	0	0	0	0	0	0	0	0	0	0
	exad1-1	NaCl (100mM)	0	0	0	10	0	0	0	0	0	18	0	0	0	61	53	0	13	29	0	0	0	0	0	0	28	0	23	0	8	0	0	0	0	0	0	0	0	0	0	0	0	0
	exad1-3	NaCl (100mM)	0	0	0	9	0	0	0	0	0	18	0	0	0	59	54	0	13	31	0	0	0	0	0	0	32	0	27	0	9	0	0	0	0	0	0	0	0	0	0	0	0	0
	exad1-3	NaCl (100mM)	0	0	0	9	0	0	0	0	0	19	0	0	0	64	48	5	15	30	0	0	0	0	0	0	38	0	27	0	8	0	0	0	0	0	0	0	0	0	0	0	0	0
	exad1-3	NaCl (100mM)	0	0	0	10	0	0	0	0	0	17	0	0	0	40	34	6	18	30	0	0	0	0	0	0	30	0	22	0	9	0	0	0	0	0	0	0	0	0	0	0	0	0

Supplementary Figure S7. Heatmap of Comprehensive Microarray Polymer Profiling (CoMPP) spot signals (Supports Figure 4). 5-day-old seedlings of *exad1-1*, *exad1-3* and Col-0 were transferred to liquid medium containing NaCl (100 mM) or Control (0 mM) for 48 h treatment before harvesting. AIR samples were used extracted for CoMPP analysis. Genotypes, treatments and extraction conditions of sample are shown on the left. The corresponding monoclonal antibodies (mAbs) with specificity are shown on the top (Table S5). Signal values were correlated to color intensity and shown with 3 biological replicates (approximately 20 seedlings were pooled in a tube as 1 replicate).

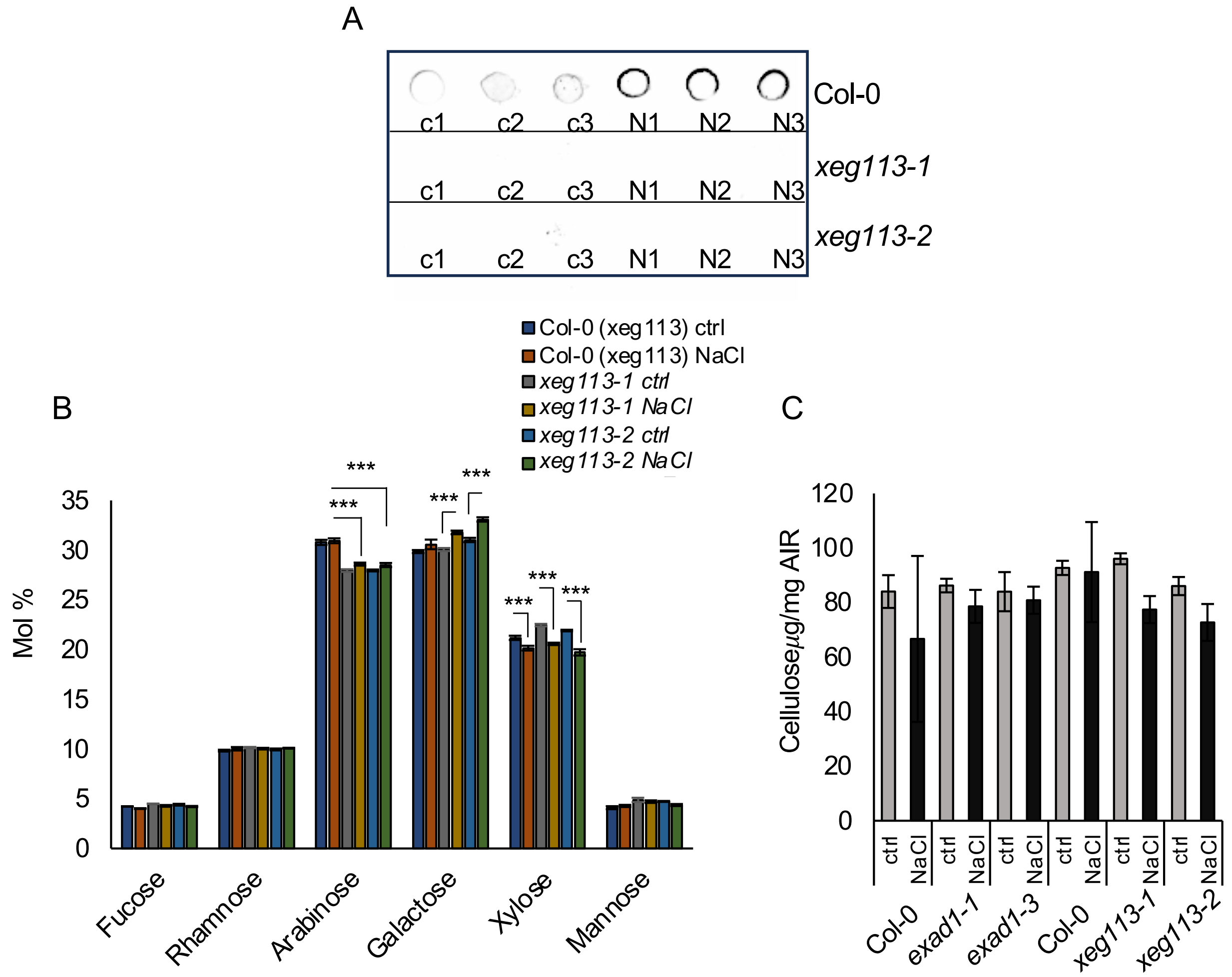

Supplementary Figure S8. JIM1 signals and cell wall composition analysis in control vs. salt-treated Col-0, *exad1*, and *xeg113* mutant seedlings. (Supports Figure 4). **A**, Five-day old seedlings of Col-0, *xeg113-1* and *xeg113-2* mutants were treated with or without 100 mM NaCl for 48 h before harvesting. Total protein were extracted and 25 ug of total protein spotted on nitrocellulose membrane. JIM11 antibody was used to detect the specific Hyp-Ara$_4$ signal. Dot-blot images are representative of 2 independent experiments performed each containing 3 biological replicates per treatment [Control (c1, c2, c3), or NaCl (N1, N2, N3)] per genotype. **B,** Five-day old seedlings of Col-0 *xeg113-1* and *xeg113-2* mutants were treated with 100 mM NaCl or without salt (Ctrl) for 48 h. Neutral cell wall matrix components were extracted from AIRs derived from 3 biological replicates per treatment/genotype and expressed as Molar Percentage (Mol%). Error bars represent SD of the mean values analyzed for each biological replicate (n= 3). Asterisks indicate statistically significant differences according to Student's t-test compared to Col-0 NaCl (for Arabinose) or compared to the corresponding controls (for Galactose and Xylose) (*, P< 0.05; **P< 0.01 ***P< 0.001). **C,** Cellulose content expressed as μg of Glucose/ mg AIR was analyzed in five-day old seedlings of Col-0, *exad1-1, exad1-3,* Col-0, *xeg113-1* and *xeg113-2* mutants seedlings treated as in C (n= 3). Statistical analysis performed by using two-way ANOVA with contrast post-hoc. No significant difference has been detected.

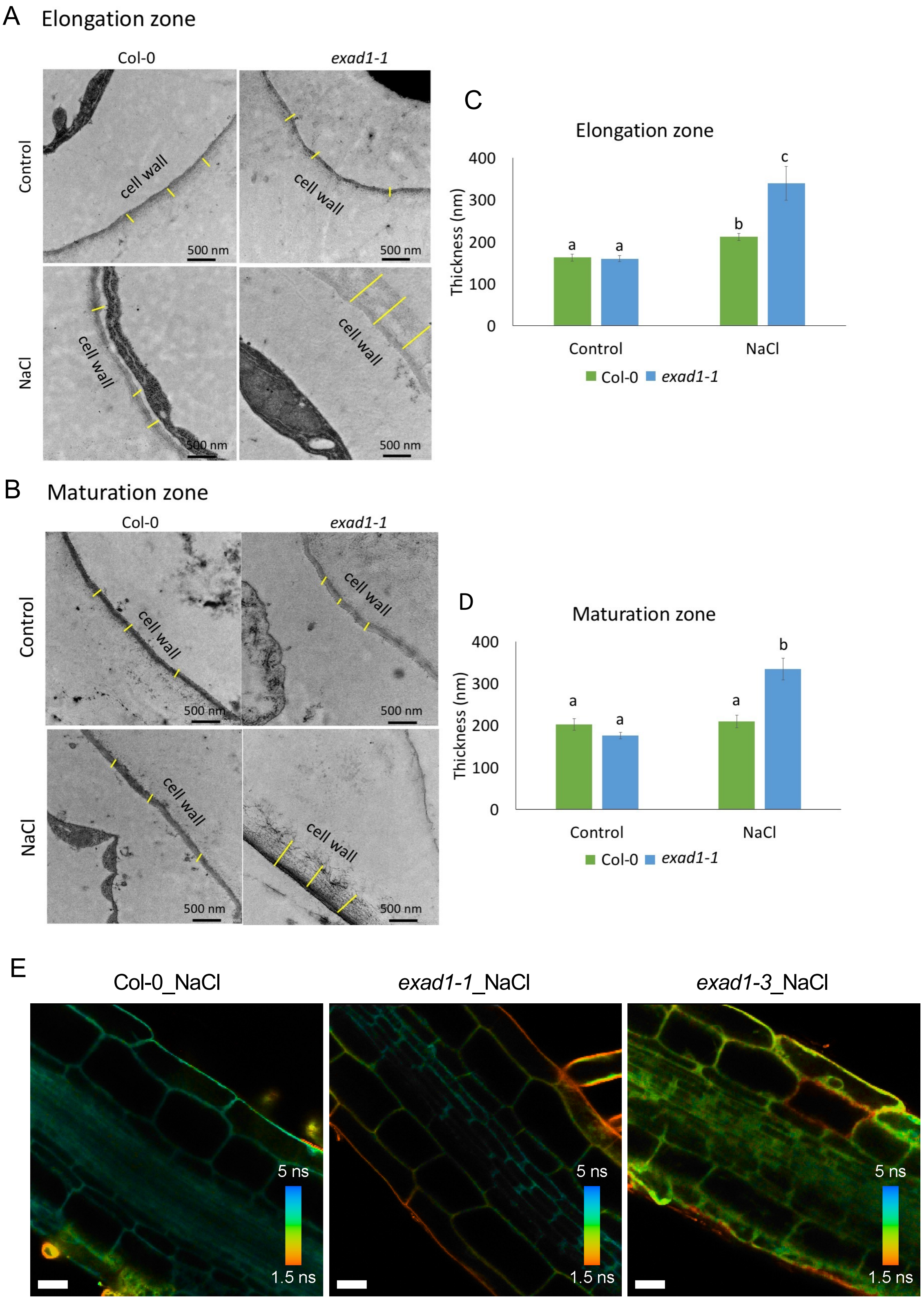

Supplementary Figure S9. Cell wall thickness and porosity analyzed in control vs. salt-treated Col-0 and *exad* mutant *lines*. (Supports Figure 5). TEM analysis showing the cell wall thickness in epidermal cells of both the elongation zone (EZ) (**A**) and maturation zone (MZ) (**B**) of seedlings roots treated with and without NaCl (100 mM) for 48 h. Four-day old seedlings of Col-0 and *exad1-1* were transferred onto new plates in the SITA system. Samples were collected after 48 h treatments for TEM imaging. Cell walls and scale bar (= 500 nm) are indicated in the images. Quantifications of cell wall thickness in the elongation zone (**C**) and maturation zone (**D**) were measured from randomized selected 3 positions of cell wall (indicated as yellow lines) for each image. 3 biological replicates were used (each seedling as 1 biological replicate), consisting of 18 independent cells. Cell walls were measured at 3 randomized positions on each of the analyzed cells per biological replicate. Statistical analysis was done using two-way ANOVA followed with contrasts post-hoc test, different letters indicate significant differences according to P-value < 0.05. **E,** Representative pictures of the seedlings analyzed in Figure 5C. Col-0, *exad1-1* and *exad1-3* mutant seedlings were treated with 100 mM NaCl for 48 h, stained with the CWP-BDP and then subjected to FLIM analysis to detect the intensity of the signal. The samples underwent excitation using a pulsed laser source at a wavelength of 514 nm (with a pulse duration of less than 1 ps) at a repetition rate of 40 MHz (scale bars represent 25 um) (n= 15).

Supplementary Table S1. List of accessions used in this study.

CS_code	*Name*	*Nordborg ID*	*CS_code*	*Name*	*Nordborg ID*	*CS_code*	*Name*	*Nordborg ID*
CS22689	RRS-10	1	CS28780	Tsu-0	1477	CS76186	MNF-Jac-32	2881
CS28007	Aa-0	13	CS28786	Ty-0	1489	CS76187	MNF-Pot-48	2893
CS28013	Alst-1	25	CS28787	Uk-1	1501	CS76188	MNF-Pot-68	2905
CS28014	Amel-1	37	CS28788	Uk-2	1513	CS76189	MOG-37	2917
CS28017	An-2	49	CS28795	Utrecht	1525	CS76190	Mr-0	2929
CS28018	Ang-0	61	CS28800	Ven-1	1537	CS76191	Mrk-0	2941
CS28049	Ann-1	73	CS28804	Wa-1	1549	CS76192	Mt-0	2953
CS28051	Arby-1	85	CS28808	Wag-3	1561	CS76193	Mz-0	2965
CS28053	Ba-1	97	CS28809	Wag-4	1573	CS76194	N13	3001
CS28054	Baa-1	109	CS28810	Wag-5	1585	CS76195	Na-1	3013
CS28063	Be-1	121	CS28812	WAR	1597	CS76196	NC-6	3025
CS28064	Benk-1	133	CS28814	Wc-2	1609	CS76197	Nd-1	3037
CS28090	Blh-2	145	CS28822	Wl-0	1621	CS76198	NFA-10	3047
CS28091	Boot-1	157	CS28823	Ws	1633	CS76199	NFA-8	3059
CS28097	Bs-2	169	CS28833	Wt-3	1645	CS76200	√ñm√∂2-1	3071
CS28099	Bsch-0	181	CS28847	Zu-1	1657	CS76201	√ñr-1	3083
CS28108	Bu-8	193	CS28848	Ors-1	1669	CS76202	Ost-0	3095
CS28128	Ca-0	205	CS28849	Ors-2	1681	CS76203	Oy-0	3107
CS28133	Cha-0	217	CS76083	11ME1.32	1693	CS76205	PAR-3	3119
CS28135	Chat-1	229	CS76084	11PNA4.101	1705	CS76206	PAR-4	3131
CS28140	CIBC-2	241	CS76085	328PNA054	1717	CS76207	PAR-5	3145
CS28141	CIBC-4	253	CS76086	627ME-4Y1	1729	CS76208	Paw-3	3157
CS28142	CIBC-5	265	CS76087	Ag-0	1741	CS76209	Pent-1	3169
CS28158	Cit-0	277	CS76088	Alc-0	1753	CS76210	Per-1	3181
CS28163	Co-2	289	CS76089	ALL1-2	1765	CS76211	Petergof	3193
CS28165	Co-4	301	CS76090	ALL1-3	1777	CS76212	PHW-34	3205
CS28181	CSHL-5	313	CS76091	An-1	1789	CS76213	Pna-17	3217
CS28193	Com-1	325	CS76092	App1-16	1801	CS76214	Pro-0	3229
CS28200	Da-0	337	CS76093	B√•1-2	1813	CS76215	Pu2-23	3241
CS28201	Da(1)-12	349	CS76094	Bay-0	1825	CS76216	Ra-0	3253
CS28202	Db-0	361	CS76095	Belmonte-4-94	1837	CS76217	Rak-2	3265
CS28208	Di-1	373	CS76096	Bg-2	1849	CS76218	Ren-1	3277
CS28210	Do-0	385	CS76097	Bla-1	1861	CS76219	Rev-2	3289
CS28214	Dra-2	397	CS76098	Blh-1	1873	CS76220	Rmx-A180	3301
CS28217	Ede-1	409	CS76099	Bor-1	1885	CS76221	ROM-1	3313
CS28236	Ep-0	421	CS76100	Bor-4	1897	CS76222	Rsch-4	3325
CS28241	Es-0	433	CS76101	Br-0	1909	CS76223	Sanna-2	3337
CS28243	Est-0	445	CS76102	Br√∂1-6	1921	CS76224	Sap-0	3349

CS28252	Fi-1	469	CS76103	Bu-0	1933	CS76225	Sav-0	3373
CS28268	Fr-4	481	CS76104	BUI	1945	CS76226	Se-0	3385
CS28274	Ga-2	493	CS76105	Bur-0	1957	CS76227	Sha	3397
CS28277	Ge-1	505	CS76106	C24	1969	CS76228	SLSP-30	3409
CS28279	Gel-1	517	CS76107	CAM-16	1981	CS76229	Sparta-1	3421
CS28280	Gie-0	529	CS76108	CAM-61	1993	CS76230	Sq-8	3433
CS28282	Go-0	541	CS76109	Can-0	2005	CS76231	St-0	3445
CS28326	Gr-5	553	CS76110	Cen-0	2017	CS76232	Ste-3	3457
CS28332	Gu-1	565	CS76111	CIBC-17	2029	CS76233	T1040	3469
CS28336	Ha-0	577	CS76112	CLE-6	2041	CS76234	T1060	3481
CS28343	Hau-0	589	CS76113	Col-0	2053	CS76235	T1080	3493
CS28344	Hey-1	601	CS76114	Ct-1	2065	CS76236	T1110	3505
CS28345	Hh-0	613	CS76115	CUR-3	2077	CS76238	T510	3517
CS28350	Hn-0	625	CS76116	Cvi-0	2089	CS76239	T540	3529
CS28364	Je-0	649	CS76119	DraIV1-14	2101	CS76240	T620	3541
CS28369	Jl-3	661	CS76120	DraIV1-5	2113	CS76242	Ta-0	3553
CS28373	Jm-1	673	CS76121	DraIV1-7	2125	CS76243	T√ÖD	3565
CS28382	Kelsterbach-2	685	CS76122	DraIV6-16	2137	CS76244	Tamm-2	3577
CS28394	Kl-5	697	CS76123	DraIV6-35	2149	CS76245	TDr-1	3589
CS28395	Kn-0	721	CS76124	Duk	2161	CS76247	TDr-18	3601
CS28407	KNO-11	733	CS76125	Eden-2	2173	CS76248	TDr-3	3613
CS28419	Kr-0	745	CS76126	Edi-0	2185	CS76249	TDr-8	3625
CS28420	Kro-0	757	CS76127	Est-1	2197	CS76250	Tomegap-2	3637
CS28423	Krot-2	769	CS76128	F√§b-4	2209	CS76251	Tottarp-2	3647
CS28454	Li-3	781	CS76129	Fei-0	2221	CS76252	TOU-A1-115	3657
CS28457	Li-5:2	793	CS76131	Fj√§1-2	2233	CS76253	TOU-A1-116	3669
CS28459	Li-6	805	CS76132	Fj√§1-5	2245	CS76254	TOU-A1-12	3681
CS28461	Li-7	817	CS76133	Ga-0	2257	CS76255	TOU-A1-43	3693
CS28490	Mc-0	829	CS76134	Gd-1	2269	CS76256	TOU-A1-62	3705
CS28492	Mh-0	841	CS76135	Ge-0	2281	CS76257	TOU-A1-67	3717
CS28495	Mnz-0	853	CS76136	Got-7	2293	CS76258	TOU-A1-96	3729
CS28510	N4	865	CS76137	Gr-1	2305	CS76259	TOU-C-3	3741
CS28513	N7	877	CS76138	Gul1-2	2317	CS76260	TOU-E-11	3753
CS28527	Nc-1	889	CS76139	Gy-0	2329	CS76261	TOU-H-12	3765
CS28550	NFC-20	901	CS76140	Hi-0	2341	CS76262	TOU-H-13	3777
CS28564	No-0	913	CS76141	Hod	2353	CS76263	TOU-I-17	3789
CS28568	Nok-1	937	CS76142	Hov4-1	2365	CS76264	TOU-I-2	3813
CS28573	Nw-0	949	CS76143	Hovdala-2	2377	CS76265	TOU-I-6	3825
CS28575	Nw-2	961	CS76144	HR-5	2389	CS76266	TOU-J-3	3837
CS28578	Nz1	973	CS76145	Hs-0	2401	CS76267	TOU-K-3	3849
CS28580	Ob-1	1009	CS76146	HSm	2413	CS76268	Ts-1	3861
CS28583	Old-1	1021	CS76147	In-0	2425	CS76269	UduI1-34	3873
CS28587	Or-0	1033	CS76148	JEA	2437	CS76270	UKID101	3885

CS28595	Pa-2	1057	CS76149	Ka-0	2449	CS76272	UKID37	3897
CS28610	PHW-10	1069	CS76150	Kas-1	2461	CS76273	UKID48	3909
CS28613	PHW-13	1081	CS76151	KBS-Mac-8	2473	CS76274	UKID80	3921
CS28614	PHW-14	1093	CS76152	Kelsterbach-4	2485	CS76275	UKNW06-059	3933
CS28620	PHW-20	1105	CS76153	Kin-0	2497	CS76276	UKNW06-060	3969
CS28622	PHW-22	1117	CS76154	Kno-18	2509	CS76277	UKNW06-386	3981
CS28626	PHW-26	1129	CS76155	PHW-3	2521	CS76278	UKNW06-436	3993
CS28628	PHW-28	1141	CS76156	Kulturen-1	2533	CS76279	UKNW06-460	4005
CS28631	PHW-31	1153	CS76157	LAC-3	2557	CS76280	UKSE06-062	4017
CS28633	PHW-33	1165	CS76158	LAC-5	2569	CS76281	UKSE06-192	4029
CS28635	PHW-35	1177	CS76159	Lc-0	2581	CS76282	UKSE06-272	4041
CS28636	PHW-36	1189	CS76160	LDV-14	2593	CS76283	UKSE06-278	4053
CS28637	PHW-37	1201	CS76161	LDV-25	2605	CS76284	UKSE06-349	4065
CS28640	Pla-0	1213	CS76162	LDV-34	2617	CS76285	UKSE06-351	4077
CS28645	Pn-0	1225	CS76163	LDV-58	2629	CS76286	UKSE06-414	4089
CS28650	Pog-0	1237	CS76164	Ler-1	2641	CS76287	UKSE06-429	4101
CS28651	Pr-0	1249	CS76165	LI-OF-095	2653	CS76288	UKSE06-466	4113
CS28663	Pu2-24	1261	CS76166	Liarum	2665	CS76289	UKSE06-482	4125
CS28685	Rhen-1	1273	CS76167	Lill√∂-1	2677	CS76290	UKSE06-520	4137
CS28692	Rou-0	1285	CS76168	Lip-0	2689	CS76291	UKSE06-628	4161
CS28713	RRS-7	1297	CS76169	Lis-1	2701	CS76292	UKSW06-202	4173
CS28720	S96	1309	CS76172	LL-0	2713	CS76293	Ull2-3	4185
CS28724	Sapporo-0	1321	CS76173	Lm-2	2725	CS76295	Ull3-4	4197
CS28725	Sav-0	1333	CS76174	Lom1-1	2737	CS76296	Uod-7	4209
CS28729	Sei-0	1345	CS76175	L√∂v-5	2749	CS76297	Van-0	4221
CS28732	Sg-1	1369	CS76176	Lp2-2	2761	CS76298	V√•r2-1	4233
CS28734	Sh-0	1381	CS76177	Lp2-6	2773	CS76299	VOU-1	4245
CS28739	Si-0	1393	CS76179	Lz-0	2785	CS76300	VOU-2	4257
CS28743	Sp-0	1405	CS76180	Map-42	2797	CS76301	Wei-0	4269
CS28750	Ste-0	1417	CS76181	MIB-15	2809	CS76302	Wil-1	4281
CS28758	Tha-1	1429	CS76182	MIB-22	2821	CS76303	Ws-0	4293
CS28759	Ting-1	1441	CS76183	MIB-28	2833	CS76304	Wt-5	4305
CS28760	Tiv-1	1453	CS76184	MIB-84	2857	CS76305	Yo-0	4317
CS28779	Tscha-1	1465	CS76185	MNF-Che-2	2869	CS76306	Zdr-6	4329
CS76307	ZdrI2-24	4353	CS76308	ZdrI2-25	4365			

Supplementary Table S2. List of candidate loci identified with GWAS via GWAPP web tool.

Trait	*chromosome*	*position*	*score*	*maf*	*mac*
$RTD^{N\text{-}C}$ at 5hrs	5	19655414	5.6065944	0.22492401	74
	5	19655217	5.55250178	0.20668693	68
	5	19654675	5.2946252	0.2431611	80
$RVA^{N\text{-}C}$ at 23hrs	3	21339391	7.20899264	0.09146342	30
	3	21337671	6.24919415	0.09146342	30
	3	21337695	6.16611667	0.08841463	29
	3	21342174	5.75853634	0.08841463	29
	3	21340210	5.51252194	0.08841463	29
$_{K}RTD^{N/C}$	4	11430063	7.38831601	0.12131148	37
	4	11431504	6.99860872	0.10163935	31

Supplementary Table S3. List of candidate loci identified with GWAS via R package.

traits	*chr*	*SNP-position*	*LOD score*	*MAC*	*MAF*	*variance_explained*	*heritability*	*AGI code*	*gene location*	*Gene description*
5h RTD^{N-C}	5	19652832	6.6904479	45	0.13677812	0.07958767	Control: 27.87 % NaCl: 32.56 %	AT5G48485	19646028 - 19646840	DIR1
		19652891	6.6904479	45	0.13677812	0.07958767		AT5G48490	19647784 - 19648362	DIR1-LIKE
		19652892	6.6904479	45	0.13677812	0.07958767		AT5G48500	19652980 - 19654355	NOI9
		19652911	6.72195301	46	0.13981763	0.07998411		AT5G48510	19658080 - 19658938	BTB/POZ domain-containing protein
		19652917	6.6904479	45	0.13677812	0.07958767		AT5G48515	19659857 - 19660201	defensin-like (DEFL) family protein
		19654815	6.28554304	93	0.28267477	0.07448346		AT5G48520	19661203 - 19666436	AUGMIN subunit3, AUG3
23h RVA^{N-C}	3	21337671	6.26610804	30	0.09146341	0.07445808	Control: 71.6 % NaCl: 66.08 %	AT3G57600	21332774 - 21333918	DREB subfamily
		21337695	6.07104489	29	0.08841463	0.07198593		AT3G57610	21334192 - 21336720	Adenylosuccinate Synthase, ADSS
		21339391	7.16497	30	0.09146341	0.08579915		AT3G57620	21337532 - 21339300	Galactose Oxidase-Like 4, GOXL4
		21340210	5.4948387	29	0.08841463	0.06466351		AT3G57630	21339274 - 21343964	Extensin Arabinose Deficient Transferase EXAD
		21342174	5.71024718	29	0.08841463	0.06740417		AT3G57640	21344708 - 21346236	Protein kinase superfamily protein, ZRK15
KRTD$^{N/C}$	4	11430063	7.27045042	42	0.13249211	0.09003916	NA	AT4G02590	1137487 – 1140540	UNE12
		11431504	7.46707674	34	0.10725552	0.09258381				
		11432016	8.07046146	34	0.10725552	0.10036067				
		11432671	8.32238141	35	0.11041009	0.10359291		AT4G02600	1143888 - 1147562	MLO1
		11430063	7.36648666	37	0.12131148	0.09473798				
		11431504	6.97792105	31	0.10163934	0.08951846				
		11432016	7.15900549	31	0.10163934	0.09195352				

Supplementary Table S4. Primers used in this study.

gene	***gene name***	***line***	***Forward Primer***	***Reverse Primer***	***SALK number***
AT3G57630	*ExAD*	*exad1-1*	AATGCATGAATCCAGAACAGG	AAACACCAGAGATGCCATACG	SAIL_843_G12, (Møller et al., 2017)
		exad1-3	AAATCCACATGACTCTGGCAC	TGTAAACCCATGACGTTTTCC	SALK_204414C, (Møller et al., 2017)
AT2G35610	*Xeg113*	*xeg113-1*	ATGCGAGGCATCAAATAACAC	CCACATTGCATTTTCCTTAGC	SALK_151754
		xeg113-2	AATCTTTCTTCTCGCTCCTGC	ACAATGCAGGAGGTTTCATTG	SALK_066991
qPCR primers					
gene	***gene name***	***Forward Primer***	***Reverse Primer***		
AT2G43770	HK1(housekeeping gene)	TATCATTGGATCTTGCAGTAGTG	ACATCGTCGATTCTAAAGACTTC		
At2G28390	HK2(housekeeping gene)	AACTCTATGCAGCATTTGATCCACT	TGATTGCATATCTTTATCGCCATC		
AT3G57630	ExAD_qPCR	GCGCAAAAATTACTGGGAAA	ATCACATCTTCTGCGTGCTG		

Supplementary Table S5. Antibodies used in the Comprehensive Micro Array Polymer Profiling (CoMPP) analysis.

Antibody/CBM	*Specificity*	*Reference*	*Dilution*	*Source*
JIM5	Binds to partially methyl esterified HG	Willats et al. (2000) Carbohydr. Res. 327, 309-320; VandenBosch et al. (1989) EMBO Journal 8, 335-342; Clausen et al. 2003	1:10	Plant Probes, Leeds university
JIM7	Binds to heavily methyl esterified HG	Willats et al. (2000) Carbohydr. Res. 327, 309-320; VandenBosch et al. (1989) EMBO Journal 8, 335-342; Clausen et al. 2003	1:10	Plant Probes, Leeds university
LM18	Binds to de-esterified HG	Verhertbruggen et al. (2009) Carbohydr. Res. 344, 1858	1:10	Plant Probes, Leeds university<br>Plant Probes, Leeds university
LM19	Binds to de-esterified HG	Verhertbruggen et al. (2009) Carbohydr. Res. 344, 1858	1:10	Plant Probes, Leeds university<br>Plant Probes, Leeds university
LM20	Binds to methyl-esterified HG	Verhertbruggen et al. (2009) Carbohydr. Res. 344, 1858	1:10	Plant Probes, Leeds university
LM7	Binds to partially methyl esterified HG	Willats *et al* . (2001) J. Biol. Chem. 276, 19404-19413 25. Laurenzi *et al* . (2001) Planta 214, 37-45	1:10	Plant Probes, Leeds university
INRA-RU2	Backbone of Rhamnogalacturonan I	Ralet et al. (2010) Planta 231:1373–1383	1:10	Donation from M.C. Ralet
INRA-RU1	Backbone of Rhamnogalacturonan I	Ralet et al. (2010) Planta 231:1373–1383	1:10	Donation from M.C. Ralet
LM5	Binds to (1–4)-ß-D-galactan	Jones et al. (1997) Plant Physiol 113: 1405-141	1:10	Plant Probes, Leeds university
LM6	Binds to (1,5)-α-L-arabinan	Willats et al. (1998) Carbohydr. Research 308, 149-152; Lee et al. (2005) Plant Cell 17, 3051-3065; Verhertbruggen et al. (2009)	1:10	Plant Probes, Leeds university
LM13	Binds to linear arabinan, highly sensitive to arabinanase	Moller et al. (2008) Glycoconjugate J. 25, 37-48	1:10	Plant Probes, Leeds university
LM16	Binds to galactan	Verhertbruggen et al. (2009) Plant Journal 59, 413-425	1:10	Plant Probes, Leeds university
LM8	Xylogalacturonan	Willats et al. (2004) Planta 218, 673-681	1:10	Plant Probes, Leeds university
LM22	(1,4)-β-D-mannan/galactomannan	Marcus et al. (2010) Plant Journal 64, 191-203	1:10	Plant Probes, Leeds university
LM21	(1,4)-β-D-mannan/galactomannan	Marcus et al. (2010) Plant Journal 64, 191-203	1:10	Plant Probes, Leeds university
LM15	Binds to xyloglucan,	Marcus *et al* . (2008) BMC Plant Biology 8, 60<br>Ruprecht et al. (2017) Plant Physiology 175, 1094-1104	1:10	Plant Probes, Leeds university
LM25	Binds to xyloglucan	Pedersen et al. (2012) J Biol Chem. 2012 Nov 16; 287(47): 39429–39438	1:10	Plant Probes, Leeds university
LM11	bind to unsubstituted xylans	McCartney et al. (2005) J. Histochem Cytochem 53, 543	1:10	Plant Probes, Leeds university
LM23	non-acetylated xylosyl in xylogalacturonan, xylan, fucoidan preps	Manabe *et al.* (2011) Plant Physiology 155, 1068-1078 38. Pedersen *et al* . (2012) J. Biol. Chem. 287, 39429-39438 39. Torode	1:10	Plant Probes, Leeds university
BS-400-4	(1®4)-b-mannan and galacto-(1®4)-b-mannan	Pettolino *et al.* (2001) *Planta* 214: 235-242	1:1000	BioSupplies Australia, cat no 400-4
JIM8	Arabinogalactan protein	McCabe et al. (1997) Plant Cell 9: 2225-2241	1:10	Plant Probes, Leeds university
JIM13	Arabinogalactan protein	Knox, et al. (1991) Plant Journal 1, 317-326; Yates et al. (1996) Glycobiology 6, 131-139	1:10	Plant Probes, Leeds university
JIM14	Arabinogalactan protein	Knox, et al. (1991) Plant Journal 1, 317-326; Yates et al. (1996) Glycobiology 6, 131-139	1:10	Plant Probes, Leeds university
JIM15	Arabinogalactan protein	Knox, *et al* . (1991) Plant Journal 1, 317-326<br>Yates *et al* . (1996) Glycobiology 6, 131-139	1:10<br>1:10	Plant Probes, Leeds university<br>Plant Probes, Leeds university
JIM16	Arabinogalactan protein	Knox, *et al* . (1991) Plant Journal 1, 317-326; Yates*et al* . (1996) Glycobiology 6, 131-139<br>Ruprecht et al. (2017) Plant Physiology 175, 1094-1104	1:10<br>1:10	Plant Probes, Leeds university<br>Plant Probes, Leeds university
JIM17	Arabinogalactan protein	Knox, et al. (1991) Plant Journal 1, 317-326; Yates et al. (1996) Glycobiology 6, 131-139	1:10	Plant Probes, Leeds university
JIM4	AGP glycan	Knox *et al* . (1989) Development 106, 47-56; Stacey*et al* . (1990) Planta 180, 285-292<br>Yates *et al* . (1996) Glycobiology 6, 131-139	1:10	Plant Probes, Leeds university
JIM20	Extensin	Smallwood et al. (1994) Plant Journal 5, 237-246; Knox et al. (1995) Planta 196, 266-270	1:10	Plant Probes, Leeds university
LM2	Arabinogalactan protein	Yates et al. (1996) Glycobiology 6(2):131-9.	1:10	Plant Probes, Leeds university
LM14	GlcA in AGP glycan	Moller et al. (2008) Glycoconjugate J. 25, 37-48,<br>Pedersen *et al* . (2012) J. Biol. Chem. 287, 39429-39438	1:10	Plant Probes, Leeds university
Mac207	Arabinogalactan protein	Pennell. et al. (1989) J. Cell Biology 108, 1967-1977; Yates et al. (1996) Glycobiology 6, 131-139	1:10	Plant Probes, Leeds university
CBM3a	Binds to crystalline cellulose with xyloglucan cross-reactivity	Blake et al. (2006) Journal of Biological Chemistry 281(39):29321-9	5:1000	Plant Probes, Leeds university
LM1	extensin	Smallwood *et al* . (1995) Planta 196, 510-522	1:10	Plant Probes, Leeds university
JIM11	extensin	Smallwood *et al* . (1994) Plant Journal 5, 237-246	1:10	Plant Probes, Leeds university
LM10	(1→4)-β-D-xylan	McCartney et al. (2005) J. Histochem Cytochem 53, 543	1:10	Plant Probes, Leeds university
LM28	Glucuronoxylan (glucuronosyl substituted xylans in several species and MeGlcA is not required for recognition)	Cornuault et al. (2015) Planta 242, 1321-1334.	1:10	Plant Probes, Leeds university
LM3	Extensin	Feng et al. (2014) Appl Microbiol Biotechnol 98:10077–10089	1:10	Plant Probes, Leeds university
JIM12	Extensin	Smallwood et al. (1994) Plant Journal 5, 237-246	1:10	Plant Probes, Leeds university
JIM19	Extensin	Smallwood et al. (1994) Plant Journal 5, 237-246; Knox et al. (1995) Planta 196, 266-270;<br>Wang et al. (1995) Planta 196, 271-276	1:10<br>1:10	Plant Probes, Leeds university<br>Plant Probes, Leeds university

Supplementary Table S6. Antibodies concentrations and signals in the mutants and conditions used in this study.

Extraction	*Geno types*	*Treatment*	*JIM5*	*JIM7*	*LM5*	*LM6*	*LM7*	*LM8*	*LM13*	*LM16*	*LM18*	*LM19*	*LM20*	*Inra RU1*	*Inra RU2*	*LM15*	*LM25*	*LM10*	*LM11*	*LM28*	*LM23*	*BS400-4*	*LM21*	*LM22*	*CBM3a*	*LM1*	*LM3*	*JIM11*	*JIM12*	*JIM19*	*JIM20*	*LM2*	*LM14*	*JIM4*	*JIM8*	*JIM13*	*JIM14*	*JIM15*	*JIM16*	*JIM17*	*MAC207*	*anti-rat*	*anti-mous*	*anti-his*
CDTA Fraction	Col-0	Control (0mM)	68	100	0	24	0	0	0	0	57	76	86	45	59	8	12	0	0	0	0	0	0	0	0	0	69	39	37	0	32	31	43	0	0	16	0	0	21	90	20	0	0	0
	Col-0	Control (0mM)	46	75	0	25	0	0	0	0	40	55	71	37	48	9	13	0	0	0	0	0	0	0	0	0	64	35	44	0	35	29	40	0	0	17	0	0	19	65	23	0	0	0
	Col-0	Control (0mM)	55	98	0	20	0	0	0	0	48	68	95	41	54	6	10	0	0	0	0	0	0	0	0	0	48	32	46	0	33	30	39	0	0	13	0	0	13	89	20	0	0	0
	exad1-1	Control (0mM)	29	25	0	14	0	0	0	0	49	67	0	32	37	11	9	0	0	0	0	0	0	0	0	0	37	0	34	0	27	20	26	0	0	9	0	0	9	29	13	0	0	0
	exad1-1	Control (0mM)	30	26	0	9	0	0	0	0	50	71	0	25	26	8	7	0	0	0	0	0	0	0	0	0	30	0	33	0	30	17	20	0	0	6	0	0	6	27	8	0	0	0
	exad1-1	Control (0mM)	30	30	0	13	0	0	0	0	52	71	0	34	36	9	10	0	0	0	0	0	0	0	0	0	34	0	36	0	28	18	19	0	0	7	0	0	10	30	9	0	0	0
	exad1-3	Control (0mM)	58	72	0	17	0	0	0	0	63	83	45	34	39	7	9	0	0	0	0	0	0	0	0	0	41	0	41	0	33	20	20	0	0	5	0	0	9	62	8	0	0	0
	exad1-3	Control (0mM)	56	73	0	28	0	0	0	0	53	72	52	38	48	9	14	0	0	0	0	0	0	0	0	0	83	0	52	0	42	32	36	0	0	13	0	0	22	66	20	0	0	0
	exad1-3	Control (0mM)	71	98	0	18	0	0	0	0	56	74	86	36	42	10	12	0	0	0	0	0	0	0	0	0	57	0	50	0	39	28	34	0	0	11	0	0	12	86	16	0	0	0
	Col-0	NaCl (100mM)	41	40	0	29	0	0	0	0	47	72	0	27	34	0	8	0	0	7	0	0	0	0	0	0	48	29	30	0	28	25	31	0	0	8	0	0	19	34	23	0	0	0
	Col-0	NaCl (100mM)	35	39	0	25	0	0	0	0	42	62	0	23	25	6	7	0	0	5	0	0	0	0	0	0	43	35	29	0	33	24	30	0	0	9	0	0	21	35	23	0	0	0
	Col-0	NaCl (100mM)	32	40	0	29	0	0	0	0	43	58	0	21	24	5	7	0	0	6	0	0	0	0	0	0	59	45	36	0	43	29	36	0	0	12	0	0	25	35	29	0	0	0
	exad1-1	NaCl (100mM)	41	57	0	25	0	0	0	0	43	64	29	18	15	6	6	0	0	0	0	0	0	0	0	0	58	0	37	0	36	24	25	0	0	0	0	0	13	53	19	0	0	0
	exad1-1	NaCl (100mM)	36	46	0	27	0	0	0	0	45	61	17	17	15	6	6	0	0	0	0	0	0	0	0	0	57	0	30	0	31	22	25	0	0	0	0	0	11	40	19	0	0	0
	exad1-1	NaCl (100mM)	49	65	0	29	0	0	0	0	56	76	40	24	23	5	8	0	0	0	0	0	0	0	0	0	61	0	33	0	32	23	28	0	0	0	0	0	12	50	22	0	0	0
	exad1-3	NaCl (100mM)	34	27	0	29	0	0	0	0	56	69	0	18	13	7	7	0	0	0	0	0	0	0	0	0	57	0	33	0	32	25	27	0	0	6	0	0	15	26	21	0	0	0
	exad1-3	NaCl (100mM)	34	28	0	31	0	0	0	0	55	69	0	12	9	6	6	0	0	0	0	0	0	0	0	0	62	0	35	0	37	28	28	0	0	6	0	0	15	24	22	0	0	0
	exad1-3	NaCl (100mM)	39	36	0	38	0	0	0	0	62	76	0	24	27	8	10	0	0	8	0	0	0	0	0	0	91	0	50	0	46	36	36	0	0	11	0	0	29	41	33	0	0	0
NaOH-Fraction	Col-0	Control (0mM)	0	0	0	0	0	0	0	0	0	16	0	8	10	44	36	0	0	19	0	0	9	0	0	0	60	35	35	0	28	12	21	0	0	0	0	0	5	0	6	0	0	0
	Col-0	Control (0mM)	0	0	0	0	0	0	0	0	0	15	0	8	9	28	30	0	0	16	0	0	0	0	0	0	67	33	37	0	34	15	19	0	0	0	0	0	9	0	5	0	0	0
	Col-0	Control (0mM)	0	0	0	0	0	0	0	0	0	16	0	8	8	31	37	0	0	16	0	0	0	0	0	0	66	30	36	0	29	14	11	0	0	0	0	0	8	0	0	0	0	0
	exad1-1	Control (0mM)	0	0	0	0	0	0	0	0	0	12	0	0	0	39	32	0	0	16	0	0	0	0	0	0	36	26	27	0	31	14	11	0	0	0	0	0	13	0	0	0	0	0
	exad1-1	Control (0mM)	0	0	0	0	0	0	0	0	0	11	0	0	0	45	33	0	0	14	0	0	0	0	0	0	21	0	19	0	17	6	7	0	0	0	0	0	0	0	0	0	0	0
	exad1-1	Control (0mM)	0	0	0	0	0	0	0	0	0	12	0	0	0	32	26	0	0	16	0	0	0	0	0	0	37	0	29	0	26	10	12	0	0	0	0	0	0	0	0	0	0	0
	exad1-3	Control (0mM)	0	0	0	0	0	0	0	0	0	10	0	0	0	34	29	0	0	12	0	0	0	0	0	0	25	0	24	0	22	9	10	0	0	0	0	0	0	0	0	0	0	0
	exad1-3	Control (0mM)	0	0	0	0	0	0	0	0	0	17	0	7	0	47	38	0	28	28	0	0	7	0	0	0	32	0	23	0	17	6	6	0	0	0	0	0	0	0	0	0	0	0
	exad1-3	Control (0mM)	0	0	0	0	0	0	0	0	0	14	0	0	0	52	45	0	0	14	0	0	0	0	0	0	38	0	29	0	26	10	8	0	0	0	0	0	0	0	0	0	0	0
	Col-0	NaCl (100mM)	0	0	0	6	0	0	0	0	0	20	0	6	5	30	37	0	6	26	0	0	0	0	0	0	51	11	30	0	0	0	0	0	0	0	0	0	0	0	0	0	0	0
	Col-0	NaCl (100mM)	0	0	0	5	0	0	0	0	0	19	0	6	0	33	36	0	7	26	0	0	0	0	0	0	55	14	31	0	0	0	0	0	0	0	0	0	0	0	0	0	0	0
	Col-0	NaCl (100mM)	0	0	0	5	0	0	0	0	0	18	0	6	6	35	36	0	0	26	0	0	0	0	0	0	54	11	32	0	0	0	0	0	0	0	0	0	0	0	0	0	0	0
	exad1-1	NaCl (100mM)	0	0	0	9	0	0	0	0	0	15	0	0	0	51	40	0	12	28	0	0	0	0	0	0	21	0	17	0	9	0	0	0	0	0	0	0	0	0	0	0	0	0
	exad1-1	NaCl (100mM)	0	0	0	9	0	0	0	0	0	15	0	0	0	51	42	0	6	26	0	0	0	0	0	0	21	0	18	0	8	0	0	0	0	0	0	0	0	0	0	0	0	0
	exad1-1	NaCl (100mM)	0	0	0	10	0	0	0	0	0	18	0	0	0	61	53	0	13	29	0	0	0	0	0	0	28	0	23	0	8	0	0	0	0	0	0	0	0	0	0	0	0	0
	exad1-3	NaCl (100mM)	0	0	0	9	0	0	0	0	0	18	0	0	0	59	54	0	13	31	0	0	0	0	0	0	32	0	27	0	9	0	0	0	0	0	0	0	0	0	0	0	0	0
	exad1-3	NaCl (100mM)	0	0	0	9	0	0	0	0	0	19	0	0	0	64	48	5	15	30	0	0	0	0	0	0	38	0	27	0	8	0	0	0	0	0	0	0	0	0	0	0	0	0
	exad1-3	NaCl (100mM)	0	0	0	10	0	0	0	0	0	17	0	0	0	40	34	6	18	30	0	0	0	0	0	0	30	0	22	0	9	0	0	0	0	0	0	0	0	0	0	0	0	0

Pectin	2F4		Ca cross linked homogala	Mouse	1/250	PlantProbes		Liners et al., 1989
	JIM5		Homogalactauronan wit	Rat	1/10	PlantProbes		Willats et al. (2000) Carbohydr. Res. 327, 309-320; VandenBosch et al. (1989) EMBO Journal 8, 335-342; Clausen et al. 2003
	JIM7		Homogalactauronan wit	Rat	1/10	PlantProbes		Willats et al. (2000) Carbohydr. Res. 327, 309-320; VandenBosch et al. (1989) EMBO Journal 8, 335-342; Clausen et al. 2003
	LM18		Partially methylesterifiec	Rat	1/10	PlantProbe	Old name: I	Verhertbruggen et al. (2009) Carbohydr. Res. 344, 1858
	LM19		Partially methylesterifiec	Rat	1/10	PlantProbe	Old name:)	Verhertbruggen et al. (2009) Carbohydr. Res. 344, 1858
	LM20		Partially methylesterifiec	Rat	1/10	PlantProbe	Old name: I	Verhertbruggen et al. (2009) Carbohydr. Res. 344, 1858
	LM7		Homogalactauronan wit	Rat	1/10	PlantProbes		Willats et al. (2001) J. Biol. Chem. 276, 19404-19413; Clausen et al. (2003) Carbohydr. Res. 338, 1797-1800
	INRA-RU2		Backbone of rhamnogala	Mouse	1/10	INRA	Old name: I	Ralet et al. 2010
	INRA-RU1		Backbone of rhamnogala	Mouse	1/10	INRA	Old name: :	Ralet et al. 2010
	LM5		(1→4)-β-D-galactan	Rat	1/10	PlantProbes		Jones et al. 1997
	LM6		(1→5)-α-L-arabinan	Rat	1/10	PlantProbes		Willats et al. (1998) Carbohydr. Research 308, 149-152; Lee et al. (2005) Plant Cell 17, 3051-3065; Verhertbruggen et al. (2009) Carbohydr. Res. 344, 1858
	LM13		Linearised (1→5)-α-L-ara	Rat	1/10	PlantProbes		Moller et al. (2008) Glycoconjugate J. 25, 37-48; Marcus et al. (2010) Plant Journal in press
	LM16		6'-β-D-galactosyl-β-(1→4	Rat	1/10	PlantProbes		Verhertbruggen et al. (2009) Plant Journal 59, 413-425
	LM8		Xylogalacturonan	Rat	1/10	PlantProbes		Willats et al. (2004) Planta 218, 673-681
	LM26		BRANCHED-GALACTAN ((	Rat		PlantProbes		Torode et al. (2018) Plant Physiol. 176, 1547-1558
Mannan	LM21		(1→4)-β-D-mannan/galac	Rat	1/10	PlantProbes		Marcus et al. (2010) Plant Journal in press
	LM22		(1→4)-β-D-	Rat	1/10			Marcus et al. (2010) Plant Journal in press
Xyloglucan	LM15		Xyloglucan (XXXG motif)	Rat	1/10	PlantProbes		Marcus et al. (2008) BMC Plant Biology 8, 60
	LM24		Xyloglucan	Rat	1/10	PlantProbe	Old name: XG1	
	LM25		Xyloglucan	Rat	1/10	PlantProbe	Old name: XG2	
Xylan	LM10		(1→4)-β-D-xylan	Rat	1/10	PlantProbes		McCartney et al. (2005) J. Histochem Cytochem 53, 543
	LM11		(1→4)-β-D-xylan/arabinc	Rat	1/10	PlantProbes		McCartney et al. (2005) J. Histochem Cytochem 53, 543
	LM23		(1→4)-β-D-xylan	Rat	1/10	PlantProbe	Old name: XGA4	
	LM27		Grass xylan/glucoronoxy	Rat		Plant probes		Cornuault et al. (2015) Planta 242, 1321-1334.
	LM28		Glucuronoxylan (glucurc	Rat				Cornuault et al. (2015) Planta 242, 1321-1334.
Glucan	JIM6		No defined epitope (Anti	Rat	1/10	PlantProbe	Anti callose like binding	
Arabinogal	JIM8		AGP	Rat	1/10	PlantProbes		McCabe et al., 1997
	JIM13		AGP	Rat	1/10	PlantProbes		Knox, et al. (1991) Plant Journal 1, 317-326; Yates et al. (1996) Glycobiology 6, 131-139
	JIM14		AGP, aldouronic acid	Rat	1/10	PlantProbes		Knox, et al. (1991) Plant Journal 1, 317-326; Yates et al. (1996) Glycobiology 6, 131-139
	JIM15		AGP	Rat	1/10	PlantProbes		Knox, et al. (1991) Plant Journal 1, 317-326; Yates et al. (1996) Glycobiology 6, 131-139
	JIM16		AGP	Rat	1/10	PlantProbes		Knox, et al. (1991) Plant Journal 1, 317-326; Yates et al. (1996) Glycobiology 6, 131-139
	JIM17		AGP	Rat	1/10	PlantProbes		Knox, et al. (1991) Plant Journal 1, 317-326; Yates et al. (1996) Glycobiology 6, 131-139
	JIM4		AGP	Rat	1/10	PlantProbes		Knox et al.1989; Yates et al.,1996
	LM2		AGP, β-linked GlcA	Rat	1/10	PlantProbes		Smallwood et al. 1996; Yates et al. 1996
	LM14		AGP, aldouronic acid	Rat	1/10	PlantProbe	Aldouronic	Moller et al. (2008) Glycoconjugate J. 25, 37-48
	MAC207		AGP, β-linked GlcA	Rat	1/10	PlantProbes		Pennell. et al. (1989) J. Cell Biology 108, 1967-1977; Yates et al. (1996) Glycobiology 6, 131-139
	LM30		AGP	Rat				Wilkinson et al. (2017) Journal of Cereal Science 74, 155-164
Extensin	LM1		Extensin	Rat	1/10	PlantProbes		Smallwood et al. (1995) Planta 196, 510-522
	LM3		Extensin	Rat	1/10	PlantProbes		
	JIM11		Extensin	Rat	1/10	PlantProbes		Smallwood et al. (1994) Plant Journal 5, 237-246
	JIM12		Extensin	Rat	1/10	PlantProbes		Smallwood et al. (1994) Plant Journal 5, 237-246
	JIM19		Extensin	Rat	1/10	PlantProbes		Smallwood et al. (1994) Plant Journal 5, 237-246; Knox et al. (1995) Planta 196, 266-270; Wang et al. (1995) Planta 196, 271-276
	JIM20		Extensin	Rat	1/10	PlantProbes		Smallwood et al. (1994) Plant Journal 5, 237-246; Knox et al. (1995) Planta 196, 266-270
Ferulate	LM9		Feruloylated (1→4)-β-D-	Rat	1/10	PlantProbes		Clausen et al. (2004) Planta 219, 1036-1041
	LM12		Feruloylate on any polym	Rat	1/10	PlantProbes		
Misc.	JIM84		Golgi	Rat	1/10	PlantProbes		
	LM4		Pea amine oxidase	Rat	1/10	PlantProbes		Wisniewski et al. (2000) Mol. Plant-Micro. Int. 13, 413; Willats et al. (2001) J. Biol. Chem. 276, 19404-19413
	JIM1		β-linked galactose, plasma membrane					
