# Supplementary material for: Arabinosylation of cell wall extensin is required for the directional response to salinity in roots
Source: Plant Cell. 2024 May 1;36(9):3328–43. doi: 10.1093/plcell/koae135 (PMC11371136; doi:10.1093/plcell/koae135)
Supplement: koae135_Supplementary_Data [file koae135_supplementary_data.zip › TPC2023BR01176DR1_Supplementary_Movie_legend.docx]

**Supplementary Data. Zou, Gigli-Bisceglia, et al. (2024). Salt modifies cell walls to change root direction. Plant Cell.**

**Supplementary Movie Legend**

**Supplementary Movie S1. Root response of Col-0 seedlings in time-lapse SITA is modulated specifically by NaCl treatment.** Four-day-old Arabidopsis Col-0 seedlings germinated on ½x MS medium were transferred to ½x MS agar plates containing different amounts of solutes (control, 100 mM NaCl, 100 mM KCl, or 200 mM sorbitol). At the same time, plates were rotated 90 degrees clockwise. Videos to record the dynamic growth response of roots in time-lapse analyses were produced by taking one image per 20 mins for each plate.
