# Supplementary material for: Arabinosylation of cell wall extensin is required for the directional response to salinity in roots
Source: Plant Cell. 2024 May 1;36(9):3328–43. doi: 10.1093/plcell/koae135 (PMC11371136; doi:10.1093/plcell/koae135)
Supplement: koae135_Supplementary_Data [file tpc.01176.2023-s09.pdf › tpc.01176.2023-s09.pdf]

THE PLANT CELL

Cell wall extensin arabinosylation is required for root directional response to salinity

Yutao Zou, Nora Gigli-Bisceglia, Eva van Zelm, Pinelopi Kokkinopoulou, Magdalena Julkowska, Maarten Besten, Thu-Phuong Nguyen, Hongfei Li, Jasper Lamers, Thijs De Zeeuw, Joram Dongus, Yuxiao Zeng, Yu Cheng, Iko Koevoets, Bodil Jorgensen, Marcel Giesbers, Jelmer Vroom, Tijs Ketelaar, Bent Petersen, Timo Engelsdorf, Joris Sprakel, Yanxia Zhang, and Christa Testerink

Review Timeline:

Submission Date:	05-Dec-2022
Editorial Decision:	25-Jan-2023
Resubmission Received:	14-Dec-2023
Editorial Decision:	22-Jan-2024
Revision Received:	29-Feb-2024
Accepted:	07-Mar-2024

Prof. Dr. Christa Testerink
Wageningen University
Wageningen 6708 PB
Netherlands

Dear Dr. Testerink:

Thank you for choosing to send your revised manuscript entitled "Cell wall extensin arabinosylation is required for root directional response to salinity" for consideration at The Plant Cell. We recognize that there has been a lot of discussion and communication about this manuscript over a number of months. Your submission has been evaluated by members of the editorial board as well as expert reviewers in your field, and we regret to inform you that we are not able to recommend the publication of this manuscript in its current form. We have had input from multiple scientists and we have solicited post-review comments.

During the post-review consultation session, we also agreed that if you could address the major points raised by the reviewers by new experiments, we would welcome a resubmission. This may be treated as a new submission, but we would attempt to use at least some of the same reviewers. Nevertheless, reviewers will be asked to assess as a new manuscript (i.e. are the claims fully supported by the data and do the results presented move the field forward?), and not only whether previous reviewer comments have been addressed.

The most important critiques in guiding our decision included: 1) A lack of structural characterization of the cell wall in the ExAD mutant via linkage analysis, additional characterization of the JIM11 binding epitope, and/or mass spectrometry analysis of extensin structure, which would be required to forge a reasonable mechanistic connection between ExAD mutation and the wall phenotypes observed here; 2) No clear explanation of how ExAD expression relates to salt-induced wall thickening, including missing wall thickness data for a complementation line (with a measured ExAD expression level); 3) A lack of clear definitions for Root Vector Angle and Tip Direction throughout the manuscript; 4) No convincing model for how ExAD activity might contribute to NaCl toxicity response.

It will be important to convince the editors and reviewers that the study adds significant new understanding(s) of mechanisms or processes, or otherwise represents a significant advance in the field, and that the major claims made are fully justified by the data presented. This includes careful consideration and explanation of the various controls used in experiments, the extent and manner of replication, and the statistical analyses used. Sampling methods and the nature of "biological replicates" should be described precisely (i.e. different plants, parts of plants, pooled tissue, independent pools of tissue, sampled at different times, etc.), along with a clear description of and rationale for any statistical analyses conducted. The reader should know exactly what was sampled; what forms the basis of the calculation of any means and other statistical variables and parameters reported. This is also necessary to ensure that proper statistical analysis was conducted.

The Plant Cell now requires authors to complete and submit an author revisions checklist upon submission of a revised manuscript. The aim of the checklist is to aid authors in preparing a high-quality manuscript, facilitate the review and assessment of revised manuscripts, and help to ensure that journal standards are maintained across the board. If your manuscript is accepted, the completed checklist will be published as supplemental material attached to the article online. Please download a copy of the checklist (a fillable PDF form) at this link, for submission with your revised manuscript: https://tpc.msubmit.net/html/Author_Revisions_Checklist.pdf.

Note also that supplemental materials should be restricted to large datasets and tables, presentation of replicates, and validation of reagents, methods, or genotypes. Any data that are used to support the main claims must be in the main manuscript. Supplemental figure legends must indicate what figure in the main manuscript is supported by the supplemental data presented.

We thank you for your interest in and support of The Plant Cell. We hope we will be able to render a more positive decision on future work.

Sincerely,

The Plant Cell Editorial Board

--- Reviewer comments:

Reviewer #1 (Comments for the Author):

The manuscript by Zou et al. developed a real-time salt-induced tilting assay (SITA) that is specific to Na+ stress. Using SITA, the authors isolated a novel root tip growth angle-related locus, encoding for the cell-wall modifying enzyme, ExAD in a GWAS analysis. The authors further confirmed ExAD controls tip growth angles using two independent mutant lines, as well as complementation. Finally, they found ExAD-dependent Hyp-arabinosylation, affects root bending responses and cell wall thickness. While the findings are novel, there are several questions that they may address to connect their study in the context of Na+ stress.

Majors:
#1, line 88-95, the authors quantified Root Vector Angle (RVA) and Root Tip Direction (RTD) and they found the root tip angle is related to NaCl. However, it is quite confusing when they also used "root growth angle" in most of the text. Does "root growth angle" equals RVA or RTD? For example, for line197 "required for regulating root angle in SITA.", should be "required for regulating root tip angle in SITA."?

#2, To demonstrate the root tip growth angle is specifically related to NaCl, they compare the response to KCl and sorbitol. To me, they should also include other Na+ salt, such as NaCO3.

#3, The authors showed that the effect of NaCl on root tip growth angle is due to toxicity but not to osmolarity. How ExAD, as a cell wall modification enzyme, contributes to the toxicity response of NaCl in terms of root growth angle? Is there any difference in accumulation of intracellular levels of Na+?

#4, line200, the author also found xeg113-1 and xeg113-2 seedlings mutants showed similar phenotype as ExAD mutants. Are xeg related loci associated with root growth angle in their GWAS analysis? They should discuss why xeg and other related cell wall modification loci are not discovered in the GWAS analysis in terms of significance like p-value.

Reviewer #2 (Comments for the Author):

Reviewer's report
Title: Cell wall extensin arabinosylation is required for root directional response to salinity

Version: 1
Reviewer's report
This manuscript describes the discovery of the cell wall-modifying enzyme Extensin Arabinose-Deficient Arabinosyltransferase (ExAD) leading to the salt-specific orientation of roots through a GWAS panel of 345 natural Arabidopsis accessions. Collectively, these results demonstrate that ExAD regulates root orientation upon salinity stress in Arabidopsis. Although the authors have collected a lot of data and information on these knock-out mutants, I have several points to make.
1. It seems to me that the authors genotyped the T-DNA insertion of the same exad mutants previously described by Møller, S.R. et al. (2017, Sci. Rep. 7: 1- 640). If so, did the authors recover the same original hair phenotype that Müller et al. discovered? Are there any changes to the root hair phenotype of the exad mutant in the presence of 100 mM NaCl? Another note is why authors spend time to do phenotyping this exad knock-out lines instead of requesting homozygous seeds from Petersen's group.
2. The scale bars for Figure 3A are missing. Is root length greater in exad-1 and exad-2 than in Col-0 at 0 h control and 100mM NaCl (Figure 3A)?
3. Did the 3 accession studies from GWAS show the same root vector angle as seen in exad-1 and exad-2? Have the authors examined any glycan phenotypes from any GWAS plants i.e. mostly from 3 accession studies?
4. What are the glycan phenotypes of exad-1 and exad-2? Based on the monoclonal antibody data through JIM11 the authors suggest that both the knockout lines are missing the side chain of an alpha linkage of A α-linked arabinofuranoses (Araf4) to hydroxyprolines (Hyps). This is really confusing. There is no evidence that JIM11 binds to Hyp-Araf4 side branches. I would suggest doing the cell wall structural characterization of exad-1 and exad-2 first through monosaccharide composition, and linkage analysis. The other way authors could isolate extensin enriched extract and measure the Araf4 side branches through ESI-MS/MS to examine the structural changes in these mutnats.
5. The cell wall thickness of the exad mutant in the epidermal layer at root maturation and in the root elongation zone was significantly higher than that of Col0 (Fig. 5A-D). The question is why exad mutants accumulate so much polysaccharide in their walls or why exad mutants develop thicker walls. Is there any relationship between thick wall and salt-specific orientation in exad-1 and exad-2? The authors claim that ExAD regulates root orientation. However, no cell wall structural data support their conclusion. At the same time, they had to look at the cellulose content and microfibril orientation of these mutants, because cellulose mainly microfibril, plays an important role in root orientation, and at the same time cellulose microfibril orientation

controls which direction a cell will grow. A question that needs to be answered is whether these basic directional/orientational changes are a direct effect from the mutant or a secondary effect due to some structural change in the cellulose.
Minor comments:
Why is JIM11 less binding to the NaOH-fraction of 100mM NaCl Col0 than to 0mM NaCl (Figure S7)? Any explanation?

Reviewer #3 (Comments for the Author):

This manuscript presents a useful resource for investigating SNPs involved in root response to salinity in Arabidopsis. Using a time-lapse imaging platform to record root bending during salt-induced tilting, the authors analyzed traits associated with root growth direction in a collection of Arabidopsis natural accessions. GWAS analysis allowed them to narrow down to ExAD gene, encoding an arabinosyltransferase that generates Hyp-Araf4 modification on extensins. The authors went further to characterize its gene functions, finding that it is required for regulating root bending in response to salinity and root cell wall thickness. However, there are a few major points that need to be addressed before this manuscript is accepted.

Major comments:

• The flow between section 1 and section 2 in the Results section needs improvement. Section 2 talks about the 3 traits used as input for GWAS: KRTDN/C, RTDN-C, and RVAN-C. RVAN-C is the trait that led to the identification of a locus on Chr3, and subsequently, the ExAD gene. However, I didn't find any detailed characterizations of the RVA trait in section 1. One way to address this is to replace the RTD data with RVA data in Figure 1B and 1D.

• 35S:ExAD in the exad-1 mutant background was used as a complementation line. This is problematic because 35S promoter was used. Ideally, the ExAD native promoter should be used instead. I understand that generating transgenic lines takes time, but it would be worthwhile at least to show ExAD gene expression level in this 35S-driven line. I didn't see this data in any supplemental figures.

• With regards to Fig. 5, I have two concerns. 1) Root epidermal cell wall was thicker than that in Col under NaCl treatment. Is this phenotype rescued in the 35S:ExAD/exad-1 complementation line? Without this data, I am not convinced that ExAD is required for maintaining wall thickness under salinity. 2) Are the cell wall thickness data presented from the concave or convex side of the bending roots? Would those two sides show any difference in cell wall thickness at all?

• How exactly does ExAD modification on extensins affect cell wall thickness? The authors need to provide some scenarios in the Discussion section.

Minor comments:

• Fig. 1A and Fig. 3A should include scale bars.
• Line 65: should be "loss-of-function"
• Line 66: remove "-" between "enzymes" and "share"
• Line 76: "that" should be "which"
• Line 125: should be "underlie"
• Line 175: remove one "under"
• Table S4 title, last sentence: "Figure S4" should be "Figures S5 and S6"?

Zou, Gigli-Bisceglia et al; Response to editorial and reviewers' comments:
Cell wall extensin arabinosylation is required for root directional response to salinity

Editorial comments:

The most important critiques in guiding our decision included:
1) A lack of structural characterization of the cell wall in the ExAD mutant via linkage analysis,
2) additional characterization of the JIM11 binding epitope, and/or mass spectrometry analysis of extensin structure, which would be required to forge a reasonable mechanistic connection between ExAD mutation and the wall phenotypes observed here;
3) No clear explanation of how ExAD expression relates to salt-induced wall thickening, including missing wall thickness data for a complementation line (with a measured ExAD expression level);
4) A lack of clear definitions for Root Vector Angle and Tip Direction throughout the manuscript;
5) No convincing model for how ExAD activity might contribute to NaCl toxicity response.

Response:
Thank you for this clear summary.
We are confident that we have now been able to strengthen the link between the molecular and cellular cell wall defects of the exad mutants and our phenotypic observations of these mutants, first of all by including two independent T-DNA alleles for all assays. We have also added additional comprehensive analyses of cell wall composition including cellulose content. Importantly, by using a novel cell wall probe that reports mechanical properties of the cell wall, we have been able to add a so far missing link that shows that molecular defects in extensin arabinosylation due to loss of ExAD function result in more porous cell wall structure, likely explaining the cell wall thickness phenotype observed using TEM (together addressing editorial point 3 and partially point 1).

We have also improved the readability based on your comments and have streamlined and simplified the descriptions of RVA and RTD (point 4) as well as explained better how we build on the previous molecular characterization of the exad mutants in control conditions (ed point 1 and 2). Together, these new results help forge the mechanical connection between ExAD mutation and the cell wall phenotypes observed (point 2), even in the absence of data on the JIM11 binding specificity. We have rephrased the JIM11 conclusions to avoid making unsupported claims about this antibody, which is not the major focus of our study.

We are enthusiastic about the new molecular genetic evidence we provide, which together with the earlier characterization by Møller et al. 2017, shows a clear link between ExAD dependent Hyp4 arabinosylation and the observed cell wall and root direction phenotypes, in our opinion. Regarding point 5 we have added data on $NaNO_3$ as requested by the reviewers, as well as ion accumulation data and halotropism assays. Together, this shows the SITA response, similar to halotropism, is highly specific to Na^+ ions, and that exad mutants are not only showing a different SITA response, but also an impaired halotropic response and increased Na and K levels, all consistent with ExAD being involved in the ion specific component of the salinity response of roots.

Finally, we have also improved the description of methods, sampling and statistics.

Reviewer #1 (Comments for the Author):

The manuscript by Zou et al. developed a real-time salt-induced tilting assay (SITA) that is specific to Na+ stress. Using SITA, the authors isolated a novel root tip growth angle-related locus, encoding for the cell-wall modifying enzyme, ExAD in a GWAS analysis. The authors further confirmed ExAD

controls tip growth angles using two independent mutant lines, as well as complementation. Finally, they found ExAD-dependent Hyp-arabinosylation, affects root bending responses and cell wall thickness. While the findings are novel, there are several questions that they may address to connect their study in the context of Na+ stress.

Majors:
#1, line 88-95, the authors quantified Root Vector Angle (RVA) and Root Tip Direction (RTD) and they found the root tip angle is related to NaCl. However, it is quite confusing when they also used "root growth angle" in most of the text. Does "root growth angle" equals RVA or RTD? For example, for line197 "required for regulating root angle in SITA.", should be "required for regulating root tip angle in SITA."?

R1. *We have carefully reviewed the relevant sections of our manuscript in response to your query regarding the terms "Root Vector Angle (RVA)," "Root Tip Direction (RTD)," and "root growth angle." We changed the text and figures, simplified the text, and explained the differences better. We thank the reviewer for this suggestion, we think the readability has greatly increased by these changes.*

#2, To demonstrate the root tip growth angle is specifically related to NaCl, they compare the response to KCl and sorbitol. To me, they should also include other Na+ salt, such as NaCO3.

R2. *We have presented evidence indicating that changes in root bending in response to NaCl can be attributed to specifically the Na^+ ions, and not Cl-. This was demonstrated through our initial experiments with KCl, highlighting the specificity of the bending response to Na^+ rather than K^+ or osmotic treatments.*
As requested by the reviewer, we have now also included a $NaNO_3$ (we chose $NaNO_3$ rather than $NaCO_3$ not to affect pH) treatment in SITA and analyzed the difference in root bending at 24, 48, and 72 hours. The results confirm that the SITA response is Na^+ ion specific and results are included in Figure S1A and incorporated in the main text. We thank the reviewer for the suggestion.

#3, The authors showed that the effect of NaCl on root tip growth angle is due to toxicity but not to osmolarity. How ExAD, as a cell wall modification enzyme, contributes to the toxicity response of NaCl in terms of root growth angle? Is there any difference in accumulation of intracellular levels of Na+?

R3. *It is not known currently how plants respond to Na^+ ions specifically. Therefore, we cannot definitely answer the question why NaCl but not osmotic stress or even KCl have a specific effect on root growth angle. We did measure Na and K level in the shoots of exad adult plants and found that they accumulate more Na and K when exposed to NaCl. The results are now included in the manuscript in the main text and in Figure 3 and S6.*

#4, line200, the author also found xeg113-1 and xeg113-2 seedlings mutants showed similar phenotype as ExAD mutants. Are xeg related loci associated with root growth angle in their GWAS analysis? They should discuss why xeg and other related cell wall modification loci are not discovered in the GWAS analysis in terms of significance like p-value.

R4. *We found that xeg113 mutants had a much less severe phenotype regarding root growth angle, and only a very minor (though statistically significant) phenotype compared to wt, and no detectable molecular cell wall defects in response to salt. In addition, as xeg113 mutant root growth was also*

affected under control conditions, this phenotype would not meet our criteria of salt-specificity that we set for identification of loci in our GWAS. Moreover, even if xeg113 would be uniquely required for SITA, this does not mean that natural variation would exist in this locus. Thus, we are not surprised that it does not show up in our SITA GWAS analysis.

Reviewer #2 (Comments for the Author1.

Reviewer's report
This manuscript describes the discovery of the cell wall-modifying enzyme Extensin Arabinose-Deficient Arabinosyltransferase (ExAD) leading to the salt-specific orientation of roots through a GWAS panel of 345 natural Arabidopsis accessions. Collectively, these results demonstrate that ExAD regulates root orientation upon salinity stress in Arabidopsis. Although the authors have collected a lot of data and information on these knock-out mutants, I have several points to make.

It seems to me that the authors genotyped the T-DNA insertion of the same exad mutants previously described by Møller, S.R. et al. (2017, Sci. Rep. 7: 1- 640).

If so, did the authors recover the same original hair phenotype that Müller et al. discovered?

Are there any changes to the root hair phenotype of the exad mutant in the presence of 100 mM NaCl?

Another note is why authors spend time to do phenotyping this exad knock-out lines instead of requesting homozygous seeds from Petersen's group.

R1. *We appreciate the feedback from the reviewer, and we sincerely apologize for any confusion caused. The exad mutant lines we used in our study were previously characterized in Møller et al. 2017. These seeds were obtained from NASC long ago, and we genotyped them ourselves, but we should have named them according to their previously given names Møller et al., 2017 . To align with the nomenclature established in Møller et al., 2017, we have now revised the names of the lines: what was previously labeled as exad1 is now exad1-1, and exad2 is now exad1-3. Additionally, we conducted a detailed characterization of the mutants at the cell wall level, confirming the defects in arabinose content as reported in Møller et al.. We did not further characterize the root hair phenotypes of these mutants, but added a description of these previous findings to the discussion.*

2. The scale bars for Figure 3A are missing. Is root length greater in exad-1 and exad-2 than in Col-0 at 0 h control and 100mM NaCl (Figure 3A)?

R2. *Thanks for noticing this and the scale bars have been added. FigS6E shows that root length is similar for wt and exad mutants under both control and salt conditions.*

3. Did the 3 accession studies from GWAS show the same root vector angle as seen in exad-1 and exad-2? Have the authors examined any glycan phenotypes from any GWAS plants i.e. mostly from 3 accession studies?

R3. *We incorporated the root bending analysis mentioned into Figure S5. Additionally, we conducted the JIM 11 analysis on these lines; however, we could not identify any conclusive findings/difference. Consequently, we opted not to include this analysis in the final manuscript figures. Considering the*

outcomes from S5C, we decided not to explore natural variation any further. Instead, we included another independent allele for ExAD.

4. What are the glycan phenotypes of exad-1 and exad-2? Based on the monoclonal antibody data through JIM11 the authors suggest that both the knockout lines are missing the side chain of an alpha linkage of A α-linked arabinofuranoses (Araf4) to hydroxyprolines (Hyps). This is really confusing. There is no evidence that JIM11 binds to Hyp-Araf4 side branches. I would suggest doing the cell wall structural characterization of exad-1 and exad-2 first through monosaccharide composition, and linkage analysis. The other way authors could isolate extensin enriched extract and measure the Araf4 side branches through ESI-MS/MS to examine the structural changes in these mutnats.

R4. *In the Møller et al.,2017 paper, both cell wall analysis and linkage analysis were conducted, revealing the presence of Hyp-Araf1, Hyp-Araf2, and Hyp-Araf3, with a complete absence of Hyp-Araf4 in the exad1-1 mutant. In our COMPP analysis for this manuscript, we employed various Hyp-Ara recognizing antibodies known to identify extensin epitopes including JIM11, LM3, JIM12, and JIM20 (Smallwood et al., 1994; Smallwood et al., 1995; Castilleux et al., 2020). Our results indicate that most of these antibodies exhibited a typical recognition pattern. However, only the JIM11 signal appeared to be absent in the exad1 mutants, suggesting that the epitope recognized by JIM11 specifically arises from the function of EXAD. Additional experiments revealed that plants with altered XEG113 function exhibited a similar JIM11 recognition pattern as the exad1 mutants (Figure S8A). This suggests that the addition of the third β 1-2 arabinofuranose might be necessary for the incorporation of the α 1-3 arabinofuranose in these plants. In the exad mutants, the second and third β-arabinose residues remained intact, as demonstrated by JIM20 recognition. This is consistent with Castilleux et al., 2020, where JIM20, likely identifying the second and third β-arabinose residues of extensins, failed to detect signal in cell wall extracts of rra2 rrra3 and xeg113-3 mutants. However, the signal persisted in the exad1 mutants, indicating the presence of intact second and third 1→2-linked β-arabinofuranoses. Our cell wall analysis also demonstrated that the arabinosyl transferase responsible for adding the earlier arabinofuranoses exhibited significant reductions in arabinose, while the exad mutants showed a mild reduction, likely linked to the absence of the fourth arabinofuranose only (Figure S4C and S8B). Despite these findings, we have refrained from making definitive claims about JIM11 labeling and now we state in the text that the specific target of JIM11 remains unknown. The idea that LM1, JIM11, and JIM20 recognize the third to fourth arabinofuranose was previously suggested by Castilleux et al. in 2020. Although it could contribute to a more detailed definition of specific extensin epitopes, this level of analysis exceeds the focus of the current study.*

5. The cell wall thickness of the exad mutant in the epidermal layer at root maturation and in the root elongation zone was significantly higher than that of Col0 (Fig. 5A-D). The question is why exad mutants accumulate so much polysaccharide in their walls or why exad mutants develop thicker walls. Is there any relationship between thick wall and salt-specific orientation in exad-1 and exad-2? The authors claim that ExAD regulates root orientation. However, no cell wall structural data support their conclusion. At the same time, they had to look at the cellulose content and microfibril orientation of these mutants, because cellulose mainly microfibril, plays an important role in root orientation, and at the same time cellulose microfibril orientation controls which direction a cell will grow. A question that needs to be answered is whether these basic directional/orientational changes are a direct effect from the mutant or a secondary effect due to some structural change in the cellulose.

R5. *Thank you for these comments and questions. We have removed the claim about orientation in our study, and instead have added data on root bending in halotropism (response away from high salt on a gradient) for the mutants, as depicted in Figure 3C. Interestingly, we observed a reduced ability to bend in response to salt stress gradients in both exad1 mutants compared to wildtype in the halotropism assay, corroborating the SITA results. To understand the underlying factors, we quantified cellulose content in the same samples used for the analysis of cell wall matrix monosaccharide composition. Under our experimental conditions, we did not detect any significant difference in cellulose content that could account for the drastic changes in root bending observed in these plants under salt stress (Figure S8C). To better understand cell wall alterations, we introduced an analysis of cell wall porosity/density using a probe previously described in Michels et al. 2020 (Figure 5C). This approach allowed us to link the cell wall defects observed in exad1 mutants to changes in cell wall porosity in response to salt stress. It is plausible that glycosylation malfunctions in extensins, which serve as crosslinks for pectins, could impact cell wall porosity and thickness. In our specific case, we hypothesize that the "under-crosslinking" situation in exad1 mutants might lead to reduced cross-connections between cell wall components, potentially affecting porosity without significantly* altering cellulose content.

Minor comments:
Why is JIM11 less binding to the NaOH-fraction of 100mM NaCl Col0 than to 0mM NaCl (Figure S7)? Any explanation?

R6. *We do not have a definitive answer to this question. The possibility of NaCl altering the total content of extensins in strongly crosslinked cell wall components is plausible, but we did not delve into the details of this assay. In our dot blot analysis, we processed freshly harvested salt-treated seedlings by boiling them in a buffer. This method aimed to solubilize the extensin epitopes, which we then detected via dot blot. Using this approach, we observed similar patterns to those in the COMPP analysis. However, in the COMPP analysis, we did not detect a significant increase in the JIM11 signal either, even though we observed this in all experiments conducted with wild-type salt-challenged seedlings. It is possible that some extensin epitopes are lost during the AIR (Alcohol Insoluble Residue) isolation and fractionation of cell wall components. However, this is speculative, and we did not explore this aspect further.*

Reviewer #3 (Comments for the Author):

This manuscript presents a useful resource for investigating SNPs involved in root response to salinity in Arabidopsis. Using a time-lapse imaging platform to record root bending during salt-induced tilting, the authors analyzed traits associated with root growth direction in a collection of Arabidopsis natural accessions. GWAS analysis allowed them to narrow down to ExAD gene, encoding an arabinosyltransferase that generates Hyp-Araf4 modification on extensins. The authors went further to characterize its gene functions, finding that it is required for regulating root bending in response to salinity and root cell wall thickness. However, there are a few major points that need to be addressed before this manuscript is accepted.

Major comments:

1. The flow between section 1 and section 2 in the Results section needs improvement. Section 2 talks about the 3 traits used as input for GWAS: KRTDN/C, RTDN-C, and RVAN-C. RVAN-C is the trait that led to the identification of a locus on Chr3, and subsequently, the ExAD gene. However, I didn't find any detailed characterizations of the RVA trait in section 1. One way to address this is to replace the RTD data with RVA data in Figure 1B and 1D.

R1. *We appreciate the reviewer's comment. In response, we have made significant revisions to both the figures and the text to enhance the comprehensive understanding of the variables analyzed and the methods employed. To provide a clearer presentation, we have relocated certain aspects to the supplemental figures.*

2. 35S:ExAD in the exad-1 mutant background was used as a complementation line. This is problematic because 35S promoter was used. Ideally, the ExAD native promoter should be used instead. I understand that generating transgenic lines takes time, but it would be worthwhile at least to show ExAD gene expression level in this 35S-driven line. I didn't see this data in any supplemental figures.

R2. *We agree with the reviewer that the 35S line is far from ideal to draw conclusions about the function of EXAD. We acknowledge the reviewer's point that including a line where EXAD is driven by its own promoter would be desirable, yet despite major efforts, we have not been able to clone the full length promoter. Additionally, considering that the 35s line only partially complemented the SITA phenotype and moreover, failed to complement the 4th arabinofuranose addition to the extensin epitope, as previously reported by Møller et al., 2017, making it hard to draw direct conclusions from this line, we made the decision to omit the 35S data from the manuscript. Instead we now included the independent exad1-3 mutant in all experiments (not just the SITA phenotyping as in the previous version). Using the two independent alleles exad1-1 and exad1-3, our claims are now supported by additional experiments, including cell wall analysis, thickness, and porosity assessments conducted on both mutant alleles.*

3. With regards to Fig. 5, I have two concerns. 1) Root epidermal cell wall was thicker than that in Col under NaCl treatment. Is this phenotype rescued in the 35S:ExAD/exad-1 complementation line? Without this data, I am not convinced that ExAD is required for maintaining wall thickness under salinity. 2) Are the cell wall thickness data presented from the concave or convex side of the bending roots? Would those two sides show any difference in cell wall thickness at all?

R3. *As mentioned in response to the previous point, we made the decision to exclude the analysis of the 35s line, and instead performed additional TEM experiments on another exad allele, which confirmed our earlier data (Figure 5A). In addition, we included two xeg113 mutant lines and found no difference in cell wall thickness in these mutants compared to wt.*
Unfortunately, maintaining the root orientation posed challenges, making it impossible to analyze thickness uniformly. To address this limitation, we conducted a comprehensive analysis by examining cell thickness in specific root zones (elongation or maturation) around the stele. The observed variations in thickness are likely influenced by different areas being in contact with salt. To prevent any confusion we removed the claim about orientation from our study.

4. How exactly does ExAD modification on extensins affect cell wall thickness? The authors need to provide some scenarios in the Discussion section.

R4. *As requested by this reviewer we have proposed a mechanism, which is now detailed in the discussion section. We thank the reviewer for their critical questions and suggestions for additional experiments, which allowed us to strengthen our conclusions. Based on new experiments using a cell wall probe that reports porosity of the cell wall, which was found to be higher in both exad1 mutant alleles compared to wt, and more robust genetic data using both exad1 alleles for all experiments, we propose a possible scenario in the discussion section. In summary, our findings suggest that plants respond to cell wall modifications caused by salt stress by enhancing crosslinks mediated by arabinosyl transferases, specifically those mediated by the ExAD1. ExAD1 uniquely targets the 4th*

α1-2 arabinofuranose, a characteristic feature of extensins. This targeted response serves as a crucial mechanism for plants to counteract the alterations in the cell wall structure induced by salt stress. In control conditions and in the absence of ExAD1, we observed a wild-type-like cell wall thickness, which becomes significantly thicker (when compared to that of salt-treated wt seedlings) in the presence of salt. In fact even if NaCl increases cell wall thickness also in wild-type plants, likely due to changes in pectin methylation or cell wall loosening this effect is likely counteracted by the function of ExAD1, which elevates arabinosylation levels on extensins. In a situation where the function of the EXAD1 is impaired and the plant cannot reinforce the cell wall by increasing crosslinks, the cell wall swells and becomes more porous. This increased porosity might be linked to enhanced thickness and could be influenced by alterations in changes of cell wall strength. Consequently, these changes in thickness and porosity alter the plant's ability to bend, representing a mechanical consequence of the cell wall modifications induced by salt stress.

Minor comments:

- Fig. 1A and Fig. 3A should include scale bars.
- Line 65: should be "loss-of-function"
- Line 66: remove "-" between "enzymes" and "share"
- Line 76: "that" should be "which"
- Line 125: should be "underlie"
- Line 175: remove one "under"
- Table S4 title, last sentence: "Figure S4" should be "Figures S5 and S6"?

R5. Thank you. We have meticulously addressed all minor comments and extensively rewritten the manuscript, resulting in a new version.

1st Revision - Editorial Decision22-Jan-2024

Dear Dr. Christa Testerink:

We have received reviews of your manuscript entitled "Cell wall extensin arabinosylation is required for root directional response to salinity." On the basis of the advice received, the board of reviewing editors would like to accept your manuscript for publication in The Plant Cell. This acceptance is contingent on revision based on the comments of our reviewers. In particular, please consider addressing the minor points raised by the reviewers, as well as the following:

Point 7 in Reviewer 1's comments was also raised in the previous round of review. The authors tried to address it by making complementation lines, but they had some difficulty in cloning. Given that this is not indicated anywhere in the current version of the manuscript, perhaps the authors could at least describe their effort in the main text.

Please highlight all changes and include a detailed annotation of changes of the text, with line numbers, and noting your responses to the comments.

To submit your revised manuscript, click:

Link Not Available

If you have any questions about the revision submission procedures, please contact the Editorial Office Staff (tpc-submissions@aspb.org). If you cannot return the revised manuscript within 30 days, please let us know. Otherwise, we will assume that you have elected not to revise the manuscript and withdraw it.

Thank you very much for the privilege of reviewing this work. I look forward to receiving the next version.

Sincerely,

The Plant Cell Editorial Board

Reviewer #1 (Comments for the Author):

The manuscript by Zou et al., reported the isolation of genetic locus of Na+ -specific root directional gene ExAD, encoding cell wall extension arabinosylation using GWAS. They found that the exad mutants, which lack extensin Hyp-Araf4 modifications, display increased root epidermal cell wall thickness and porosity and altered gravitropic root bending in salt (halotropism response), as well as a reduced salt avoidance response. While the finding is interesting, there are several weaknesses that the authors need to address before publications.

Major:
1, I think, the title could be more specific to Na+, something like "Cell wall extensin arabinosylation is required for root directional response to Na+ salinity"

2. line 180-181. They measured in 345 natural Arabidopsis accessions where some of them showed variable responses. Accessions with Haplotype1 display significantly higher RVA values after 23 h salt treatment in SITA than Haplotype2 accessions. Are accessions with different Haplotype associated with the NaCl condition of the soil where they were collected? If the author have these information, they may discuss this.

3, line265-268. If I understand correctly, the exad mutants were insensitive to "salt-induced modulation of root gravitropic bending and halotropism response (Figure 3), reduced levels of Na and K in roots,
and a significantly thicker cell wall structure under salt conditions (Figure 5B, C)". Do the exad mutants grow better in salt in terms biomass of shoot under salt stress?

4, Line200 "Moreover, adult exad1-1 mutant shoots displayed enhanced Na and K" vs.
Line331 "exad mutant roots of plants exposed to salinity had higher levels of Na and K," vs.
line307. "reduced levels of Na and K in roots, and a significantly thicker cell wall structure under salt conditions (Figure 5B, C),". Which one is true, they should clarify this? They also should discuss why shoot and root showed difference.

5, It is nuclear how the angles were measured in Fig2A. The angles could be different dependent how far the green dots were selected. How far the green dot is from the tip?

6, Fig3A. To make a fair comparison, the author should grow col-0, exad1-1, exad1-3 under the same condition, ideally in a same agar plate. Is there any reason why the three plants were not imaged in a same field?

7, To my understanding, complementation experiment should be considered to prove the function of genes, although they have two independent T-DNA mutant lines. Another way to do this could be using some gene editing tools such as CRISPR to change specific nucleotides in the natural Arabidopsis accessions that are defect in ExaD (line177-180). If it is too difficult, they should discuss this so that readers can judge the work themselves.

Minors
"halotropism" should be "halotropic" for line288 and line307

"encoding for" should be "encoding" for line31 (Abstract), line70, line302

Reviewer #3 (Comments for the Author):

In this revised manuscript, I'm overall satisfied with how the authors addressed previous reviewers' comments. The flow of the manuscript is improved, and the conclusions are supported with newly added data, including monosaccharide composition, cellulose content, the use of two T-DNA alleles in every assay, and the use of a fluorescent dye that measures cell wall mesh size. These data, taken together, helped the authors come up with possible scenarios about how ExAD functions in the wall to mediate salt response in roots.

I have a few minor comments that need to be addressed:

1. Fig. 4B: one can still see faint dots in exad1-1, especially under the control condition. The authors need to tone down their argument in line 238.
2. Supplemental video: need to add a scale bar and time stamps.
3. Fig. S9: need to include cell wall thickness data in exad1-3 mutant under the control condition. Also, thickness in panels C and D should be presented in nm instead of pixels.
4. Fig. 5C: the authors should also include some FLIM images in Col, exad1-1, and exad1-3 in addition to the histogram.
5. I understand that there is a 5-figure limit for manuscripts submitted as breakthrough reports. But including a model (perhaps adding a panel in Fig. 5?) would be helpful for readers to understand the mechanistic link between ExAD-mediated processes in the wall and root response to salt.

Please also note the following:

-The Plant Cell now requires authors to complete and submit an author revisions checklist upon submission of a revised manuscript. The aim of the checklist is to aid authors in preparing a high-quality manuscript, facilitate the review and assessment of revised manuscripts, and help to ensure that journal standards are maintained across the board. If your manuscript is accepted, the completed checklist will be published as supplemental material attached to the article online. Please download a copy of the checklist (pdf fillable form) at this link, for submission with your revised manuscript: https://tpc.msubmit.net/html/Author_Revisions_Checklist.pdf.

-Supplemental materials should be restricted to large datasets and tables, presentation of replicates, and validation of reagents, methods, or genotypes. Any data that are used to support the major claims must be in the main manuscript. Supplemental figure legends must indicate what figure in the main manuscript is supported by the supplemental data presented. Please justify how each of the supplemental figures meet the criteria.

-Sampling methods and the nature of "biological replicates" should be described precisely (i.e. different plants, parts of plants, pooled tissue, independent pools of tissue, sampled at different times, etc.), along with a clear description of and rationale for any statistical analyses conducted. The reader should know exactly what was sampled; what forms the basis of the calculation of any means and statistical parameters reported. This is also necessary to ensure that proper statistical analysis was conducted.

Response to reviewers Zou et al revision 2024

Point 7 in Reviewer 1's comments was also raised in the previous round of review. The authors tried to address it by making complementation lines, but they had some difficulty in cloning. Given that this is not indicated anywhere in the current version of the manuscript, perhaps the authors could at least describe their effort in the main text.

E1: Thanks for this comment. We agree it is useful to add this information, but we are not sure how we could incorporate it in the main text. In alignment with the reviewer's suggestion to maintain transparency, we intend to present the reviewer's comments alongside the final version of the manuscript. This approach will allow readers to observe the exchanges between us and the reviewers, ensuring maximum transparency throughout the publication process.

Reviewer #1 (Comments for the Author):

The manuscript by Zou et al., reported the isolation of genetic locus of Na+ -specific root directional gene ExAD, encoding cell wall extension arabinosylation using GWAS. They found that the exad mutants, which lack extensin Hyp-Araf4 modifications, display increased root epidermal cell wall thickness and porosity and altered gravitropic root bending in salt (halotropism response), as well as a reduced salt avoidance response. While the finding is interesting, there are several weaknesses that the authors need to address before publications.

Major:
1, I think, the title could be more specific to Na+, something like "Cell wall extensin arabinosylation is required for root directional response to Na+ salinity"
R1. Thank you for your feedback. We would like to use the term "salinity" in the title while highlighting the specificity to sodium (Na+) in the abstract. While we have taken your advice into consideration, we believe it is also important to acknowledge that the treatment involves high salinity, and not only Na^+ ions.
.
2. line 180-181. They measured in 345 natural Arabidopsis accessions where some of them showed variable responses. Accessions with Haplotype1 display significantly higher RVA values after 23 h salt treatment in SITA than Haplotype2 accessions. Are accessions with different Haplotype associated with the NaCl condition of the soil where they were collected? If the author have these information, they may discuss this.
R2. Unfortunately, we do not have specific information regarding the composition of the soil. However, we have provided a map that illustrates the localization of the haplotype1 and 2 group distribution (added in Figure S5B). It's worth noting that these accessions have been purchased from NASC and it is likely that they have undergone multiple propagation events with soil composition potentially different from that of the original sampling locations.

3, line265-268. If I understand correctly, the exad mutants were insensitive to "salt-induced modulation of root gravitropic bending and halotropism response (Figure 3), reduced levels of Na and K in roots, and a significantly thicker cell wall structure under salt conditions (Figure 5B, C)". Do the exad mutants grow better in salt in terms biomass of shoot under salt stress?

R3. First of all, we apologize for the mistake regarding the statement on Na+ and K+ levels in roots (as mentioned in the next point). We did not observe a significant difference in biomass between wild-type and *exad1* mutants. However, it's important to note that these findings are preliminary. To

include them in the manuscript, we would need to conduct further experiments, which would require approximately 4 months to complete. Therefore, we would prefer not to add these data at this time.

4, Line200 "Moreover, adult exad1-1 mutant shoots displayed enhanced Na and K" vs.
Line331 "exad mutant roots of plants exposed to salinity had higher levels of Na and K," vs.
line307. "reduced levels of Na and K in roots, and a significantly thicker cell wall structure under salt conditions (Figure 5B, C),".
Which one is true, they should clarify this? They also should discuss why shoot and root showed difference.
We sincerely thank the reviewer for catching this erroneous statement in our manuscript. We acknowledge that we only measured and reported ion measurements in the shoots of plants grown in a hydroponic setup. Hence we have corrected the statement from '*exad1* mutant roots of plants exposed to salinity had higher levels of Na and K' to '*exad1* mutant <u>shoots</u> of plants exposed to salinity had higher levels of Na and K'. Additionally, we have removed the following part of the sentence (strikethrough): *'In our study, the exad1-1 and exad1-3 loss of function mutants showed reduced salt-induced modulation of root gravitropic bending and halotropic response (Figure 3~~), reduced levels of Na and K in roots,~~ and a significantly thicker cell wall structure under salt conditions (Figure 5B, C), which may be linked to possible extensin cross-linking defects in the exad1 mutants'.*
We are grateful to the reviewer for identifying and bringing this error to our attention.

5, It is unclear how the angles were measured in Fig2A. The angles could be different dependent how far the green dots were selected. How far the green dot is from the tip?
R5. The Root Vector Angle (RVA) defines the angle of the vector deviating from the gravity vector, aligning with the direction of the root tip from the moment of tilting (time 0/position 0) until the timepoint indicated. The RVA is then expressed as an average of directionality of this duration. Please note that the dots in Fig3A were just for reference to know the position of the root tip were transferred. Root length and angle were always measured by analysis of the path of the root itself.

6, Fig3A. To make a fair comparison, the author should grow col-0, exad1-1, exad1-3 under the same condition, ideally in a same agar plate. Is there any reason why the three plants were not imaged in a same field?
R6. In each experiment, we used multiple plates, with each plate containing multiple seedlings of the same genotype. Our aim was to maximize the number of replicates to ensure a robust and reliable outcome. It's worth noting that we used the same growth medium for all plates, prepared simultaneously to maintain consistency across experimental conditions. Given the extensive number of replicates analyzed, we are confident that any potential differences between genotypes are not caused by differences in the individual plates.

7, To my understanding, complementation experiment should be considered to prove the function of genes, although they have two independent T-DNA mutant lines. Another way to do this could be using some gene editing tools such as CRISPR to change specific nucleotides in the natural Arabidopsis accessions that are defect in ExaD (line177-180). If it is too difficult, they should discuss this so that readers can judge the work themselves.
R7. Thank you for your comment. We acknowledge your observation, and it's worth noting that in the initial version of our manuscript, we indeed included the 35S:ExAD line previously published by Møller et al. However, our experimental observations revealed that this line did not fully complement its arabinosylation phenotype, consistent with the findings reported by Møller in 2017. Our attempts to generate an ExAD construct driven by its native promoter for insertion into the t-DNA insertion line

were unsuccessful due to the inability to amplify the promoter sequence. This limitation resulted in the failure to produce the complementation line as initially intended. Considering these challenges, we opted to utilize two independent t-DNA lines to strengthen and reinforce our findings.

Minors
"halotropism" should be "halotropic" for line288 and line307
We thank this reviewer, we made the changes requested.
"encoding for" should be "encoding" for line31 (Abstract), line70, line302
We thank this reviewer, we made the changes requested.

Reviewer #3 (Comments for the Author):

In this revised manuscript, I'm overall satisfied with how the authors addressed previous reviewers' comments. The flow of the manuscript is improved, and the conclusions are supported with newly added data, including monosaccharide composition, cellulose content, the use of two T-DNA alleles in every assay, and the use of a fluorescent dye that measures cell wall mesh size. These data, taken together, helped the authors come up with possible scenarios about how ExAD functions in the wall to mediate salt response in roots.

I have a few minor comments that need to be addressed:

1. Fig. 4B: one can still see faint dots in exad1-1, especially under the control condition. The authors need to tone down their argument in line 238.
We thank this reviewer; we made the changes requested. The sentence has been replaced with the following: interestingly, almost no JIM11 signal was detected in the *exad1* mutant alleles.

2. Supplemental video: need to add a scale bar and time stamps.
We thank this reviewer; we replaced the video, now containing time stamps and scale bars.

3. Fig. S9: need to include cell wall thickness data in exad1-3 mutant under the control condition.
We appreciate the reviewer's suggestion to include cell wall thickness data for the *exad1-3* mutant under control conditions in Fig. S9. However, since our initial cell wall phenotyping with one allele only showed a phenotype in salt, we repeated the analysis with the additional allele (in the previous revision) exclusively under salt conditions, not under control conditions. This decision was also influenced by resource limitations. We hope the reviewer will understand and accept our rationale for this approach.

Also, thickness in panels C and D should be presented in nm instead of pixels.
We thank this reviewer; we made the changes requested. The new analyses are depicted in S9C and D.

4. Fig. 5C: the authors should also include some FLIM images in Col, exad1-1, and exad1-3 in addition to the histogram.
We thank this reviewer; we made the changes requested. The representative pictures can now be found in Figure S9E.

5. I understand that there is a 5-figure limit for manuscripts submitted as breakthrough reports. But including a model (perhaps adding a panel in Fig. 5?) would be helpful for readers to understand the mechanistic link between ExAD-mediated processes in the wall and root response to salt.

While we truly appreciate your suggestion and acknowledge that it would be useful to depict the role of ExADs in regulating cell wall thickness in response to salt, our understanding of the targets and the precise mechanisms through which this regulation occurs in both mutant and wild-type conditions remains incomplete. Considering the limitations in our current knowledge, we are inclined to avoid presenting a highly speculative model, which may not be appropriate at this time. We hope you understand our decision in this matter.

Please also note the following:

-The Plant Cell now requires authors to complete and submit an author revisions checklist upon submission of a revised manuscript. The aim of the checklist is to aid authors in preparing a high-quality manuscript, facilitate the review and assessment of revised manuscripts, and help to ensure that journal standards are maintained across the board. If your manuscript is accepted, the completed checklist will be published as supplemental material attached to the article online. Please download a copy of the checklist (pdf fillable form) at this link, for submission with your revised manuscript: https://tpc.msubmit.net/html/Author_Revisions_Checklist.pdf.

Confirmed

-Supplemental materials should be restricted to large datasets and tables, presentation of replicates, and validation of reagents, methods, or genotypes. Any data that are used to support the major claims must be in the main manuscript. Supplemental figure legends must indicate what figure in the main manuscript is supported by the supplemental data presented. Please justify how each of the supplemental figures meet the criteria.

Confirmed

-Sampling methods and the nature of "biological replicates" should be described precisely (i.e. different plants, parts of plants, pooled tissue, independent pools of tissue, sampled at different times, etc.), along with a clear description of and rationale for any statistical analyses conducted. The reader should know exactly what was sampled; what forms the basis of the calculation of any means and statistical parameters reported. This is also necessary to ensure that proper statistical analysis was conducted.

Confirmed

07-Mar-2024

Dear Dr. Christa Testerink:

We are pleased to inform you that your paper entitled "Cell wall extensin arabinosylation is required for root directional response to salinity" has been accepted for publication in The Plant Cell, pending a final editorial review by a science editor. At this stage, your manuscript will be evaluated by a science editor with respect to its presentation of scientific content, compliance with journal policies, and presentation for a broad readership. The Plant Cell has contracted with Plant Editors (planteditors.com) to provide this service to our authors, and you will soon receive additional information on this process.

Please note that each author needs to link their ORCID identifier to their account before your manuscript can be published. If any authors do not have an ORCID linked to their account, they will receive a message with a link to complete this task. Please follow up with your coauthors and ensure that they have completed this task as soon as possible.

The Plant Cell and The Arabidopsis Information Resource (TAIR) are collaborating to collect functional annotation data about Arabidopsis genes from authors. This includes information about the gene's molecular function (e.g., kinase activity, ATP synthetase activity), the biological process/es it is involved in (e.g., endosperm development, threonine biosynthesis), its subcellular location (e.g., nucleus, ER), anatomical or developmental expression pattern (e.g., leaf, ovule, flower stage 10, seedling stage), or its partner in a protein-protein interaction (e.g., AT1G01010 interacts with AT1G01020).

If your paper contains results falling into one or more of these categories for Arabidopsis genes, we request that you now submit these data for inclusion in TAIR by filling in the form provided at the following URL: https://www.arabidopsis.org/doc/submit/functional_annotation/123. If you need further clarification on what types of data can be submitted please contact curator@arabidopsis.org.

Finally, we encourage your submission of artwork for the journal cover. Monthly issues will have an online cover image and selected covers will be used for posters, other promotional items, and "wallpaper" for mobile devices. For more information, refer to Cover Submission in the Instructions for Authors https://tpc.msubmit.net/cgi-bin/main.plex?form_type=display_auth_instructions.

We look forward to seeing your paper published.

Sincerely,

The Plant Cell Editorial Board

----FOR ASPB OFFICE USE ONLY (DO NOT EDIT)----
MSID: 37387
Corresponding Author: Prof. Dr. Testerink
